# Synthesis and In Vitro Evaluation of Novel Dopamine Receptor D_2_ 3,4-dihydroquinolin-2(1*H*)-one Derivatives Related to Aripiprazole

**DOI:** 10.3390/biom11091262

**Published:** 2021-08-24

**Authors:** Radomir Juza, Kristyna Stefkova, Wim Dehaen, Alena Randakova, Tomas Petrasek, Iveta Vojtechova, Tereza Kobrlova, Lenka Pulkrabkova, Lubica Muckova, Marko Mecava, Lukas Prchal, Eva Mezeiova, Kamil Musilek, Ondrej Soukup, Jan Korabecny

**Affiliations:** 1 National Institute of Mental Health, Topolova 748, 250 67 Klecany, Czech Republic; juza25@seznam.cz (R.J.); Kristyna.Stefkova@nudz.cz (K.S.); tomas.petrasek@nudz.cz (T.P.); vojtechova.Iveta@seznam.cz (I.V.); eva.mezeiova@gmail.com (E.M.); 2 Department of Chemistry, University of Hradec Kralove, Rokitanskeho 62, 500 03 Hradec Kralove, Czech Republic; kamil.musilek@uhk.cz; 3 CZ-OPENSCREEN: National Infrastructure for Chemical Biology, Department of Informatics and Chemistry, Faculty of Chemical Technology, University of Chemistry and Technology Prague, Technicka 5, 166 28 Prague, Czech Republic; wimdehaen@gmail.com; 4 Institute of Physiology, Czech Academy of Sciences, Videnska 1083, 142 20 Prague, Czech Republic; Alena.Randakova@fgu.cas.cz; 5 Biomedical Research Centre, University Hospital Hradec Kralove, Sokolska 581, 500 05 Hradec Kralove, Czech Republic; kobrlova.tereza@gmail.com (T.K.); lenka.pulkrabkova@fnhk.cz (L.P.); lubica.muckova@unob.cz (L.M.); markomecava@centrum.cz (M.M.); lukas.prchal@fnhk.cz (L.P.)

**Keywords:** aripiprazole, dopamine receptor, blood–brain barrier, molecular modeling studies, schizophrenia

## Abstract

In this pilot study, a series of new 3,4-dihydroquinolin-2(1*H*)-one derivatives as potential dopamine receptor D_2_ (D_2_R) modulators were synthesized and evaluated in vitro. The preliminary structure–activity relationship disclosed that compound **5e** exhibited the highest D_2_R affinity among the newly synthesized compounds. In addition, **5e** showed a very low cytotoxic profile and a high probability to cross the blood–brain barrier, which is important considering the observed affinity. However, molecular modelling simulation revealed completely different binding mode of **5e** compared to USC-D301, which might be the culprit of the reduced affinity of **5e** toward D_2_R in comparison with USC-D301.

## 1. Introduction

Dopamine primarily mediates its effect through activation of dopamine receptors (DRs) [1]. There are a total of five DR subtypes that are members of the G-protein-coupled receptors (GPCRs) [2], and are further divided into two classes according to the structure. The D_1_-like family includes D_1_Rs and D_5_Rs, whereas D_2–4_Rs belong to the D_2_-like family [1,2,3]. The main difference between both families is that D_1_-like family activate adenylate cyclase (AC), which leads to production of cyclic adenosine monophosphate (cAMP), whereas D_2_-like stimulates AC activity.

Dopamine D_2_ type receptors (D_2_Rs) are integral membrane receptors coupled to G proteins with three extracellular loops, seven transmembrane domains, and three intracellular loops [4,5]. D_2_Rs are present in two isoforms, short D_2S_ and long D_2L_, which differ by the insertion of 29 amino acids in the third intracellular loop on D_2L_Rs [6]. The “short” version is exclusively expressed presynaptically as an autoreceptor, whereas, the “long” one is mainly found at the postsynaptic cells [7]. Furthermore, both isoforms can inhibit intracellular cyclic adenosine monophosphate via G_i_ [4]. The highest levels of D_2_Rs in the human brain are expressed within striatum, the olfactory tubercle, and the nucleus accumbens, but it can be also found in the ventral tegmental area, substantia nigra, septum, amygdala, cortical areas, and hippocampus. D_2_Rs are involved in working memory, reward motivation functions, and regulation of locomotion [2,8]. D_2_Rs are the main target in the treatment of schizophrenia [9]. Indeed, antagonists or partial agonists of D_2_Rs are the main representatives for schizophrenia. Besides, they can be applied in the therapy of depression and anxiety [9,10,11,12,13].

Schizophrenia is a serious mental disorder that affects up to 1% of the population worldwide [9]. It is believed, that environmental factors (i.e., viral infection, early childhood trauma, and obstetrical complications) increase the expression of certain genes to enhance risk factors for schizophrenia out-break [14,15]. The symptoms of schizophrenia include three groups, namely (i) positive (hallucinations, disorganized speech and delusions), (ii) negative (diminished expressiveness and reduced motivation), and (iii) cognitive (impaired memory, decreased speed of mental processing and executive functions) [14]. The pathophysiology of schizophrenia is poorly understood, however, it is assumed, that dysfunction of the dopamine mesolimbic pathway is responsible for positive symptoms and mesocortical pathway for negative symptoms [9,16]. Cognitive symptoms have been suggested to result from decreased levels of dopamine in cortical areas [17]. The current approaches for the management of schizophrenia involve three generations of antipsychotics. The first antipsychotics are mainly D_2_R antagonists, the second exhibit multi-target antagonism with significant antagonism at serotonin 5-HT_2A_ type receptor (5-HT_2A_R), and the third possess a multi-target profile with partial agonism at D_2_R [9]. All licensed neuroleptics exhibited D_2_R affinity at therapeutic doses crucial for their mechanism of action [18,19,20,21]. In addition to D_2_Rs affinity, antipsychotic drugs show activity toward other dopamine (D_1_, D_3_, and D_4_) and serotonin (especially 5-HT_1A_, 5-HT_2A_, and 5-HT_2C_) receptors. However, selective D_1_, D_3_, and D_4_ receptor antagonists are not so effective in providing antipsychotic response, and D_1_Rs agonists exhibited limited clinical efficacy [22,23,24,25,26,27]. The evidence of 5-HT_1A_Rs agonism/partial agonism is inconclusive and the role of the 5-HT_2A_Rs antagonism is neither necessary nor a major contributor to antipsychotic effect [28,29,30]. Furthermore, 5-HT_2C_Rs antagonism has been associated mainly with side-effects including weight gain, diabetes, and sexual disturbances [30,31]. On the other hand, pimavanserin (an inverse agonist at 5-HT_2A_Rs) has recently been approved for the treatment of psychosis associated with Parkinson’s disease [32]. According to various studies, there is no benefit even when choosing different combinations of antipsychotics in 13–50% of all patients [33]. In addition, the current medication reduces mainly positive symptoms and causes severe side-effects such as diabetes, sexual dysfunction, weight gain, confusion, blurred vision, sedation, or dizziness, which also typically occurs during the treatment of depressive or anxiety disorders [34,35,36,37,38]. Thus, there is an enormous need to develop new effective and safe neuroleptic drugs since CNS disorders are also considered among the most expensive medical conditions (the total cost of disorders of the brain was estimated 798 billion EUR in Europe in 2010) [39].

**Figure 1:**
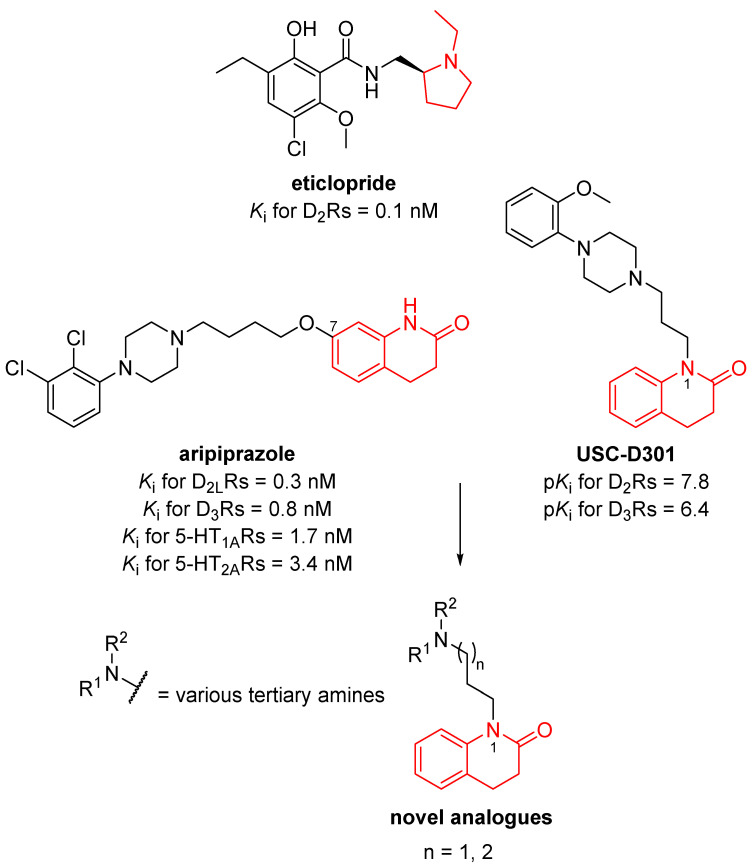
Design of novel 3,4-dihydroquinolin-2(1*H*)-one analogues from aripiprazole, eticlopride and USC-D301.

In this pilot study, we designed and synthesized a novel series of compounds based on the approved drug aripiprazole characterized by the effect via D_2_R [40], in combination with structural properties of USC-D301, highly selective D_2_R ligand [41]. Considering this effect, we have performed in vitro evaluation of novel 3,4-dihydroquinolin-2(1*H*)-one derivatives (Figure 1) for their affinity to D_2_Rs, predicted their CNS availability, and established their cytotoxicity profile in order to estimate the clinical relevance of our observations. The D_2_Rs affinity is also corroborated by the in silico simulation.

## 2. Materials and Methods

### 2.1. Chemistry

The chemicals were purchased from Sigma-Aldrich Co., LLC (Prague, Czech Republic) and were used without additional purification. Analytical thin-layer chromatography was carried out using plates coated with silica gel 60 with the fluorescent indicator F254 (Merck, Prague, Czech Republic). The thin-layer chromatography (TLC) plates were visualized by exposure to ultraviolet light (254 nm) or by the detection reagent ninhydrin. Column chromatography was performed using silica gel 100 at atmospheric pressure (70–230-mesh ASTM, Fluka, Prague, Czech Republic). The NMR spectra were all recorded on a Varian S500 spectrometer (500 MHz for ^1^H and 126 MHz for ^13^C). Chemical shifts are reported in *δ* ppm referenced to residual solvent signals (for ^1^H NMR and ^13^C NMR: chloroform-*d* (CDCl_3_; 7.26 (D) or 77.16 (C) ppm). A CEM Explorer SP 12 S was used for the MW-assisted reaction. The final compounds were analyzed by LC-MS with a Dionex Ultimate 3000 RS UHPLC system coupled with a Q Exactive Plus Orbitrap mass spectrometer (Thermo Fisher Scientific, Bremen, Germany) to obtain high-resolution mass spectra. Gradient LC analysis with UV detection (254 nm) confirmed >95% purity.

#### 2.1.1. Preparation of 1-(3-chloropropyl)-3,4-dihydroquinolin-2(1H)-one (**4a**)

To a stirred solution of 3,4-dihydroquinolin-2-(1*H*)-one (**1**) (2.7 mmoL) and 60% NaH (272 mg) in DMF (20 mL), 1-bromo-3-chloropropane (**2**) (3.0 mmoL) was added in drop-by-drop manner under ice-cooled condition. After the addition of **2**, the reaction mixture was stirred for 4 h at room temperature (r.t.) [42]. After the completion of the reaction (monitored by TLC), the mixture was diluted with toluene (30 mL) and concentrated under reduced pressure. This operation was done three times. To the resulting mixture, DCM (200 mL) and distilled water (100 mL) were added, and it was vigorously stirred at r.t. for 30 min. The organic phase was then separated, dried over Na_2_SO_4_, filtered and concentrated under reduced pressure. The crude residue was purified by silica gel chromatography (DCM:EtOAc = 4:1 *v*/*v*).

The product was isolated as yellowish oil in 89% yield (540 mg); ^1^H NMR of **4a** agrees with the literature-reported spectra [43]. ^1^H NMR (500 MHz, CDCl_3_) *δ* 7.28–7.24 (m, 1H), 7.17 (dq, *J* = 7.4, 1.2 Hz, 1H), 7.07 (dd, *J* = 8.2, 1.1 Hz, 1H), 7.01 (td, *J* = 7.4, 1.1 Hz, 1H), 4.12–4.07 (m, 2H), 3.62 (t, *J* = 6.3 Hz, 2H), 2.89 (dd, *J* = 8.7, 6.1 Hz, 2H), 2.69–2.60 (m, 2H), 2.19–2.10 (m, 2H).

#### 2.1.2. Preparation of 1-(4-chlorobutyl-3,4-dihydroquinolin-2(1H)-one (**4b**)

To a stirred solution of 3,4-dihydroquinolin-2-(1*H*)-one (**1**) (6.1 mmoL) and 60% NaH (624 mg) in DMF (18 mL), 1-bromo-4-chlorobutane (**3**) (12 mmoL) was added in a drop-by-drop manner under ice-cooled condition. After the addition of **3**, the reaction mixture was stirred at r.t. overnight [42,44,45]. After the completion of the reaction (monitored by TLC), the mixture was diluted with toluene (30 mL) and concentrated under reduced pressure. This operation was done three times. To the resulting mixture, EtOAc (300 mL) and distilled water (100 mL) were added, and it was vigorously stirred at r.t. for 30 min. The organic phase was then separated, dried over Na_2_SO_4_, filtered and concentrated under reduced pressure. The crude residue was purified by silica gel chromatography (DCM:EtOAc = 98:2 *v*/*v*).

The product was isolated as yellowish oil in 70% yield (1.0 g); ^1^H NMR (500 MHz, CDCl_3_) *δ* 7.27–7.23 (m, 1H), 7.20–7.14 (m, 1H), 7.01 (ddd, *J* = 8.5, 5.8, 1.2 Hz, 2H), 3.98 (t, *J* = 7.0 Hz, 2H), 3.58 (t, *J* = 6.1 Hz, 2H), 2.89 (dd, *J* = 8.7, 6.1 Hz, 2H), 2.67–2.58 (m, 2H), 1.82 (tdd, *J* = 9.0, 7.4, 4.9 Hz, 4H). ^13^C NMR (126 MHz, CDCl_3_) *δ* 170.4, 139.4, 128.2, 127.6, 126.7, 122.9, 114.8, 44.7, 41.2, 32.0, 29.9, 25.7, 24.7.

#### 2.1.3. General Procedure for the Preparation of Final Compounds **5a**-**g** and **6a**-**g**

To a stirred solution of appropriate analogue **4a**,**b** (0.5 mmoL) and amine **a**-**g** (1.5 mmoL) in MeCN (5 mL), K_2_CO_3_ (1.5 mmoL) was added and the reaction mixture was stirred for overnight at reflux [46]. After the completion of the reaction (monitored by TLC), the mixture was diluted with CHCl_3_ (30 mL), the solid was filtered off and the residue was concentrated under reduced pressure. The crude product was purified by silica gel chromatography (DCM:MeOH = 95:5 *v*/*v*). Final compounds (**5a**-**g**, **6a**-**g**) were prepared as hydrochlorides by mixing with small portion of hydrochloric acid (37% aq.) in MeOH at r.t.

*1-(3-(Pyrrolidin-1-yl)propyl)-3,4-dihydroquinolin-2(1H)-one* (**5a**), Colorless oil. Yield: 56% (72 mg); ^1^H NMR (500 MHz, CDCl_3_) *δ* 7.21 (td, *J* = 7.8, 1.6 Hz, 1H), 7.13 (dd, *J* = 7.5, 1.5 Hz, 1H), 7.05 (d, *J* = 8.1 Hz, 1H), 6.97 (t, *J* = 7.3 Hz, 1H), 4.01–3.95 (m, 2H), 2.86 (dd, *J* = 8.7, 6.1 Hz, 2H), 2.65–2.53 (m, 8H), 1.90 (p, *J* = 7.5 Hz, 2H), 1.79 (h, *J* = 3.1 Hz, 4H). ^13^C NMR (126 MHz, CDCl_3_) *δ* 170.3, 139.6, 128.1, 127.6, 126.6, 122.8, 115.0, 54.2, 53.7, 40.6, 32.0, 26.6, 25.6, 23.5. LC-MS: calc m/z = 259.180232 for C_16_H_23_N_2_O^+^; found [M+H]^+^ = 259.1802; 99% purity.

*1-(3-(Piperidin-1-yl)propyl)-3,4-dihydroquinolin-2(1H)-one* (**5b**), Colorless oil. Yield: 64% (87 mg); ^1^H NMR (500 MHz, CDCl_3_) *δ* 7.24–7.19 (m, 1H), 7.14 (d, *J* = 7.3 Hz, 1H), 7.08 (d, *J* = 8.2 Hz, 1H), 6.98 (t, *J* = 7.4 Hz, 1H), 3.96 (t, *J* = 7.5 Hz, 2H), 2.87 (dd, *J* = 8.7, 6.1 Hz, 2H), 2.62 (dd, *J* = 8.7, 6.1 Hz, 2H), 2.45–2.39 (m, 6H), 1.87 (p, *J* = 7.5 Hz, 2H), 1.61 (p, *J* = 5.7 Hz, 4H), 1.43 (p, *J* = 5.9 Hz, 2H). ^13^C NMR (126 MHz, CDCl_3_) *δ* 170.3, 139.7, 128.0, 127.6, 126.6, 122.8, 115.1, 56.4, 54.6, 40.6, 32.0, 25.8, 25.7, 24.6, 24.3. LC-MS: calc m/z = 273.1961 for C_17_H_25_N_2_O^+^; found [M+H]^+^ = 273.1945; 98% purity.

*1-(3-(4-Methylpiperazin-1-yl)propyl)-3,4-dihydroquinolin-2(1H)-one* (**5c**), Yellowish oil. Yield: 51% (72 mg); ^1^H NMR (500 MHz, CDCl_3_) *δ* 7.21 (td, *J* = 7.8, 1.6 Hz, 1H), 7.14 (dd, *J* = 7.4, 1.5 Hz, 1H), 7.06 (dd, *J* = 8.2, 1.0 Hz, 1H), 6.98 (td, *J* = 7.4, 1.1 Hz, 1H), 4.04–3.89 (m, 2H), 2.87 (dd, *J* = 8.7, 6.1 Hz, 2H), 2.66–2.57 (m, 2H), 2.50 (s, 8H), 2.42 (t, *J* = 7.2 Hz, 2H), 2.30 (s, 3H), 1.82 (p, *J* = 7.2 Hz, 2H). ^13^C NMR (126 MHz, CDCl_3_) *δ* 170.3, 139.7, 128.1, 127.5, 126.6, 122.8, 115.0, 55.6, 55.1, 53.0, 45.9, 40.5, 32.0, 25.7, 24.8. LC-MS: calc m/z = 288.2070 for C_17_H_26_N_3_O^+^; found [M+H]^+^ = 288.2065; 98% purity.

*1-(3-Morpholinopropyl)-3,4-dihydroquinolin-2(1H)-one* (**5d**), Yellowish oil. Yield: 45% (62 mg); ^1^H NMR (500 MHz, CDCl_3_) *δ* 7.21 (td, *J* = 7.8, 1.6 Hz, 1H), 7.14 (dd, *J* = 7.4, 1.5 Hz, 1H), 7.06 (d, *J* = 7.9 Hz, 1H), 6.98 (td, *J* = 7.4, 1.1 Hz, 1H), 4.01–3.95 (m, 2H), 3.70 (t, *J* = 4.7 Hz, 4H), 2.87 (dd, *J* = 8.7, 6.1 Hz, 2H), 2.67–2.59 (m, 2H), 2.48–2.36 (m, 6H), 1.83 (p, *J* = 7.3 Hz, 2H). ^13^C NMR (126 MHz, CDCl_3_) *δ* 170.3, 139.7, 128.1, 127.5, 126.6, 122.8, 114.9, 67.0, 56.2, 53.8, 40.5, 32.0, 25.7, 24.4. LC-MS: calc m/z = 275.1754 for C_16_H_23_N_2_O_2_^+^; found [M+H]^+^ = 275.1749; 95% purity.

*1-(3-Thiomorpholinopropyl)-3,4-dihydroquinolin-2(1H)-one* (**5e**), Colorless oil. Yield: 47% (68 mg); ^1^H NMR (500 MHz, CDCl_3_) *δ* 7.23 (td, *J* = 7.9, 1.6 Hz, 1H), 7.15 (dd, *J* = 7.5, 1.4 Hz, 1H), 7.05 (d, *J* = 8.1 Hz, 1H), 6.99 (t, *J* = 7.4 Hz, 1H), 3.99–3.93 (m, 2H), 2.88 (dd, *J* = 8.7, 6.1 Hz, 2H), 2.76–2.66 (m, 8H), 2.66–2.59 (m, 2H), 2.45 (t, *J* = 7.2 Hz, 2H), 1.83 (p, *J* = 7.2 Hz, 2H). ^13^C NMR (126 MHz, CDCl_3_) *δ* 170.3, 139.7, 128.1, 127.5, 126.6, 122.9, 114.9, 56.4, 55.1, 40.5, 32.0, 27.9, 25.7, 24.3. LC-MS: calc m/z = 291.1526 for C_16_H_23_N_2_OS^+^; found [M+H]^+^ = 291.1520; 95% purity.

*1-(3-(Diethylamino)propyl)-3,4-dihydroquinolin-2(1H)-one* (**5f**), Colorless oil. Yield: 45% (59 mg); ^1^H NMR (500 MHz, CDCl_3_) *δ* 7.23 (td, *J* = 7.8, 1.6 Hz, 1H), 7.17–7.12 (m, 1H), 7.06 (d, *J* = 8.1 Hz, 1H), 6.99 (t, *J* = 7.4 Hz, 1H), 4.00–3.93 (m, 2H), 2.87 (dd, *J* = 8.7, 6.1 Hz, 2H), 2.66–2.54 (m, 8H), 1.89–1.80 (m, 2H), 1.09–1.02 (m, 6H). ^13^C NMR (126 MHz, CDCl_3_) *δ* 170.4, 139.6, 128.1, 127.6, 126.6, 122.9, 115.0, 50.3, 46.9, 40.7, 32.0, 25.7, 24.6, 11.4. LC-MS: calc m/z = 261.1961 for C_16_H_25_N_2_O^+^; found [M+H]^+^ = 261.1958; 98% purity.

*1-(3-((2-Methoxyethyl)(methyl)amino)propyl)-3,4-dihydroquinolin-2(1H)-one* (**5g**), Yellowish oil. Yield: 52% (72 mg); ^1^H NMR (500 MHz, CDCl_3_) *δ* 7.25–7.20 (m, 1H), 7.17–7.12 (m, 1H), 7.06 (d, *J* = 8.1 Hz, 1H), 6.98 (tt, *J* = 7.4, 1.0 Hz, 1H), 3.99–3.93 (m, 2H), 3.49 (td, *J* = 5.7, 1.2 Hz, 2H), 3.34 (d, *J* = 0.7 Hz, 3H), 2.87 (dd, *J* = 8.7, 6.1 Hz, 2H), 2.65–2.57 (m, 4H), 2.55–2.48 (m, 2H), 2.30 (d, *J* = 1.4 Hz, 3H), 1.88–1.80 (m, 2H). ^13^C NMR (126 MHz, CDCl_3_) *δ* 170.3, 139.7, 128.1, 127.6, 126.6, 122.8, 115.0, 70.5, 59.0, 56.7, 55.5, 42.7, 40.6, 32.0, 25.7, 24.9. LC-MS: Calc m/z = 277.1911 for C_16_H_25_N_2_O_2_^+^; found [M+H]^+^ = 277.1910; 98% purity.

*1-(4-(Pyrrolidin-1-yl)butyl)-3,4-dihydroquinolin-2(1H)-one* (**6a**), Colorless oil. Yield: 59% (71 mg); ^1^H NMR (500 MHz, CDCl_3_) *δ* 7.25–7.18 (m, 1H), 7.14 (d, *J* = 7.3 Hz, 1H), 7.02–6.95 (m, 2H), 3.93 (d, *J* = 6.7 Hz, 2H), 2.90–2.83 (m, 2H), 2.74 (dt, *J* = 7.2, 3.6 Hz, 4H), 2.69–2.64 (m, 2H), 2.64–2.58 (m, 2H), 1.87 (h, *J* = 2.7 Hz, 4H), 1.69 (dq, *J* = 5.9, 3.4 Hz, 4H). ^13^C NMR (126 MHz, CDCl_3_) *δ* 170.4, 139.4, 128.1, 127.6, 126.6, 122.9, 114.9, 55.7, 54.0, 41.5, 32.0, 25.6, 25.1, 25.0, 23.5. LC-MS: Calc m/z = 273.1961 for C_17_H_25_N_2_O^+^; found [M+H]^+^ = 273.1957; 99% purity.

*1-(4-(Piperidin-1-yl)butyl)-3,4-dihydroquinolin-2(1H)-one* (**6b**), Colorless oil. Yield: 61% (87 mg); ^1^H NMR (500 MHz, CDCl_3_) *δ* 7.29–7.24 (m, 1H), 7.18 (d, *J* = 7.3 Hz, 1H), 7.08 (d, *J* = 8.1 Hz, 1H), 7.02 (t, *J* = 7.4 Hz, 1H), 4.00–3.94 (m, 2H), 2.90 (dd, *J* = 8.7, 6.2 Hz, 2H), 2.65 (dd, *J* = 8.8, 6.1 Hz, 2H), 2.44 (dd, *J* = 17.9, 10.2 Hz, 6H), 1.67 (qt, *J* = 12.0, 4.9 Hz, 8H), 1.48 (p, *J* = 5.9 Hz, 2H). ^13^C NMR (126 MHz, CDCl_3_) *δ* 170.3, 139.6, 128.1, 127.5, 126.6, 122.8, 115.0, 58.6, 54.5, 41.9, 32.0, 25.7, 25.1, 24.3, 23.8. LC-MS: Calc m/z = 287.2118 for C_18_H_27_N_2_O^+^; found [M+H]^+^ = 287.2118; 97% purity.

*1-(4-(4-Methylpiperazin-1-yl)butyl)-3,4-dihydroquinolin-2(1H)-one* (**6c**), Yellowish oil. Yield: 69% (104 mg); ^1^H NMR (500 MHz, CDCl_3_) *δ* 7.22 (td, *J* = 7.8, 1.6 Hz, 1H), 7.13 (dd, *J* = 7.4, 1.5 Hz, 1H), 7.05–7.00 (m, 1H), 6.97 (td, *J* = 7.4, 1.1 Hz, 1H), 3.92 (dd, *J* = 8.7, 6.5 Hz, 2H), 2.86 (dd, *J* = 8.7, 6.1 Hz, 2H), 2.64–2.57 (m, 2H), 2.53–2.46 (m, 8H), 2.42–2.35 (m, 2H), 2.29 (s, 3H), 1.65 (ddd, *J* = 15.1, 9.0, 7.0 Hz, 2H), 1.60–1.51 (m, 2H). ^13^C NMR (126 MHz, CDCl_3_) *δ* 170.2, 139.6, 128.1, 127.5, 126.6, 122.8, 115.0, 57.8, 55.0, 52.9, 45.9, 41.9, 32.0, 25.6, 25.0, 24.0. LC-MS: calc m/z = 302.2227 for C_18_H_28_N_3_O^+^; found [M+H]^+^ = 302.2228; 98% purity.

*1-(4-Morpholinobutyl)-3,4-dihydroquinolin-2(1H)-one* (**6d**), Yellowish oil. Yield: 64% (92 mg); ^1^H NMR (500 MHz, CDCl_3_) *δ* 7.23 (td, *J* = 7.8, 1.6 Hz, 1H), 7.15 (dd, *J* = 7.5, 1.5 Hz, 1H), 7.03 (d, *J* = 8.2 Hz, 1H), 6.99 (t, *J* = 7.4 Hz, 1H), 3.98–3.91 (m, 2H), 3.73 (t, *J* = 4.8 Hz, 4H), 2.87 (dd, *J* = 8.7, 6.1 Hz, 2H), 2.62 (dd, *J* = 8.7, 6.1 Hz, 2H), 2.49–2.44 (m, 4H), 2.41 (t, *J* = 7.4 Hz, 2H), 1.73–1.63 (m, 2H), 1.63–1.55 (m, 2H). ^13^C NMR (126 MHz, CDCl_3_) *δ* 170.3, 139.6, 128.1, 127.5, 126.7, 122.9, 115.0, 66.8, 58.3, 53.6, 41.8, 32.0, 25.7, 24.9, 23.5. LC-MS: calc m/z = 288.1838 for C_17_H_24_N_2_O_2_; found [M+H]^+^ = 289.1911; 96% purity.

*1-(4-Thiomorpholinobutyl)-3,4-dihydroquinolin-2(1H)-one* (**6e**), Colorless oil. Yield: 58% (88 mg); ^1^H NMR (500 MHz, CDCl_3_) *δ* 7.23 (td, *J* = 7.9, 1.6 Hz, 1H), 7.15 (dd, *J* = 7.5, 1.5 Hz, 1H), 7.03 (d, *J* = 8.2 Hz, 1H), 6.99 (t, *J* = 7.4 Hz, 1H), 3.97–3.90 (m, 2H), 2.87 (dd, *J* = 8.7, 6.1 Hz, 2H), 2.76–2.65 (m, 8H), 2.65–2.59 (m, 2H), 2.41 (t, *J* = 7.3 Hz, 2H), 1.65 (ddd, *J* = 15.1, 8.8, 6.8 Hz, 2H), 1.56 (ddd, *J* = 14.8, 8.3, 6.0 Hz, 2H). ^13^C NMR (126 MHz, CDCl_3_) *δ* 170.3, 139.6, 128.1, 127.5, 126.7, 122.8, 115.0, 58.6, 55.0, 41.9, 32.0, 28.0, 25.7, 25.0, 23.6. LC-MS: Calc m/z = 305.1682 for C_17_H_25_N_2_OS^+^; found [M+H]^+^ = 305.1683; 95% purity.

*1-(4-(Diethylamino)butyl)-3,4-dihydroquinolin-2(1H)-one* (**6f**), Yellowish oil. Yield: 49% (67 mg); ^1^H NMR (500 MHz, CDCl_3_) *δ* 7.23 (td, *J* = 7.8, 1.6 Hz, 1H), 7.15 (dd, *J* = 7.4, 1.5 Hz, 1H), 7.03 (d, *J* = 8.2 Hz, 1H), 6.99 (td, *J* = 7.4, 1.1 Hz, 1H), 3.97–3.91 (m, 2H), 2.87 (dd, *J* = 8.7, 6.1 Hz, 2H), 2.67–2.55 (m, 6H), 2.55–2.50 (m, 2H), 1.66 (ddd, *J* = 14.9, 8.9, 6.9 Hz, 2H), 1.61–1.54 (m, 2H), 1.06 (t, *J* = 7.2 Hz, 6H). ^13^C NMR (126 MHz, CDCl_3_) *δ* 170.3, 139.6, 128.1, 127.6, 126.7, 122.8, 115.0, 52.3, 46.8, 41.9, 32.0, 25.7, 25.2, 24.0, 11.3. LC-MS: Calc m/z = 275.2118 for C_17_H_27_N_2_O^+^; found [M+H]^+^ = 275.2119; 96% purity.

*1-(4-((2-Methoxyethyl)(methyl)amino)butyl)-3,4-dihydroquinolin-2(1H)-one* (**6g**), Yellowish oil. Yield: 59% (86 mg); ^1^H NMR (500 MHz, CDCl_3_) *δ* 7.23 (td, *J* = 7.8, 1.6 Hz, 1H), 7.14 (dd, *J* = 7.4, 1.5 Hz, 1H), 7.03–6.94 (m, 2H), 3.97–3.90 (m, 2H), 3.48 (td, *J* = 5.7, 1.0 Hz, 2H), 3.33 (s, 3H), 2.87 (dd, *J* = 8.7, 6.1 Hz, 2H), 2.65–2.55 (m, 4H), 2.48–2.41 (m, 2H), 2.28 (d, *J* = 1.1 Hz, 3H), 1.73–1.61 (m, 2H), 1.56 (ddd, *J* = 15.3, 8.5, 5.9 Hz, 2H). ^13^C NMR (126 MHz, CDCl_3_) *δ* 170.3, 139.6, 128.1, 127.5, 126.7, 122.8, 115.0, 70.6, 59.0, 57.7, 56.8, 42.6, 42.0, 32.0, 25.7, 25.1, 24.4. LC-MS: Calc m/z = 291.2067 for C_17_H_27_N_2_O_2_^+^; found [M+H]^+^ = 291.2068; 99% purity.

### 2.2. Molecular Studies

#### 2.2.1. Docking Simulation

Molecular docking of ligands was performed using the Molecular Operating Environment (MOE) software package [47]. The receptor used was pdb structure 6CM4, which contains a structure of the atypical antipsychotic drug and D_2_R antagonist risperidone bound to the D_2_R [48]. An induced fit docking protocol was used, with the Triangle Matcher used for placement (10.000 placements), London dG scoring function used for initial scoring (100 conformations retained), refinement with Amber10:EHT force field, and rescoring with GBVI/WSA dG (100 conformations retained and ranked by docking score). Before docking, compounds were prepared by protonation at physiological pH and energy minimization with Amber10:EHT force field.

#### 2.2.2. Thermodynamic Integration and Free Energy Calculations

Final docked conformations of risperidone, aripiprazole, USC-D301, and **5e** were generated using the preceding method. For thermodynamic integration standard settings were used (Figure S60, Supplementary Material). The used force field was Amber10:EHT. Preparation of the molecular system was done using MOE, the thermodynamic free energy calculation was performed using the PMEMD package of the AMBER molecular dynamics toolkit [47].

### 2.3. BBB Score Prediction

Blood–brain barrier (BBB) score of newly developed compounds was calculated using an algorithm defined by Gupta et al. [49]. A MarvinSketch software (ChemAxon Ltd., v. 20.15.0; https://www.chemaxon.com) was used to predict some of the physicochemical descriptors like number of aromatic ring, number heavy atoms, MWHBN (a descriptor comprising molecular weight, hydrogen bond donor, and hydrogen bond acceptors), topological polar surface area, and pK_A_.

### 2.4. Biology Evaluation

#### 2.4.1. D2 Receptor Binding Affinity–Transfection and Membrane Preparation

Chinese hamster ovary cells (CHO cells) were used for the binding experiments. About 24 h before transfection, CHO cells were plated on a 10 cm Petri dish at a density of 1.5 × 10^6^ cells and cultivated in 10 mL DMEM/Ham’s F12 supplemented with 10% heat-inactivated fetal bovine serum (FBS).

For transfection, the desired amount of linear polyethyleneimine (PEI) 25_K (Polysciences, Eppelheim, Germany) and DNA (dopamine receptor (D2) wild type, cDNA resource centre, Bloomsberg, PA, USA) were diluted separately in phosphate buffer saline (PBS). After 20 min, DNA was added to PEI solution, mixed and left for an additional 30 min. Final concentration of reactants was 6 µg plasmid DNA and 18 µg PEI per ml. PEI/DNA complex was added (1 mL/dish) and the plates were incubated for another 24 h. Then, the medium was replaced with fresh DMEM/Ham’s F12 with 10% FBS and incubated for an additional 24 h. Cells were maintained at 37 °C in a 5% CO_2-_humidified atmosphere.

About 48 h after transfection, cells were washed with PBS, mechanically detached from the dish with a plastic scraper in the ice-cold PBS, and centrifuged for 3 min at 250× *g*. Pellet was suspended in the ice-cold homogenization medium (100 mM NaCl, 20 mM Na-HEPES, 10 mM EDTA, pH 7.4) and homogenized on ice by two 30-s strokes using Polytron homogenizer (Ultra-Turrax; Janke & Kunkel GmbH & Co. KG, IKA-Labortechnik, Staufen, Germany) with a 30-s pause between strokes. Cell homogenates were centrifuged for 5 min at 1000× *g*. The supernatant was collected and centrifuged for 30 min at 30,000× *g*. Pellets were suspended in incubation medium (100 mM NaCl, 20 mM Na-HEPES, 10 mM MgCl_2_, pH 7.4), left for 30 min at 4 °C and centrifuged again for 30 min at 30,000× *g*. The membrane pellets were kept at −80 °C until use.

#### 2.4.2. D_2_R Binding Affinity–Radioligand Experiment

All radioligand binding experiments were optimized and carried out as described by El-Fakahany and Jakubik [50]. Dissociation constant K_D_ of [^3^H]-spiperone to D2Rs was determined in saturation binding experiment. Saturation experiments were performed in 800 μL volume containing: 400 μL of the membrane suspension and 400 μL of the radioligand in six increasing concentrations (ranging from 31 to 1000 pM).

Affinity of the tested compounds was determined in competition experiments with 180 pM [^3^H]-spiperone, that corresponds to triple K_D_ value ([^3^H]-spiperone, K_d_ = 60.1 ± 2.79 pM (*n* = 6)). The examined compounds were diluted in incubation buffer and tested in six concentrations (ranging from 0.1 nM–1 mM). The reactions were performed in 400 µL volume containing: 100 µL of the radioligand, 100 µL of tested substances dilution, and 200 µL of the membranes.

Nonspecific binding was determined in the presence of 10 μM unlabeled sulpiride. Membrane suspensions from both saturation and binding experiments (approximately 10 μg of membrane proteins per sample) were incubated in 96-well plates for 1 h at 25 °C in the incubation medium (100 mM NaCl, 20 mM Na-HEPES,10 mM MgCl_2_, pH = 7.4) in a shaking incubator (25 °C; PST-60HL, Biosan, Riga, Latvia).

The binding reactions were terminated by filtration of the membranes through APFC filter plate (Millipore, Prague, Czech Republic) pre-soaked with 0.5% PEI and washed with ice-cold distilled water using a Brandel cell harvester (Brandel, Gaithersburg, MD, USA). Then, filters with labelled membranes were dried. After 24 h, scintillation cocktail (Rotiszint eco plus, Carl Roth) was added to each sample and radioactivity was quantified by liquid scintillation spectrometry using Wallac Microbeta scintillation counter (Wallac, Turku, Finland). Competition binding experiments were performed per triplicate and all experiments were performed three times. Protein concentration was determined by the Lowry method in the Peterson modification [51].

#### 2.4.3. D2 Receptor Binding Affinity–Data Analysis

##### [^3^H]NMS Saturation Binding

The equilibrium dissociation constant (*K*_D_) and maximum binding capacity (B_MAX_) were determined in the saturation experiments. Non-specific binding in the presence of 10 µM sulpiride was subtracted to determine specific binding. Free concentration of [^3^H]-spiperone was calculated by subtraction of values of specific binding from the final concentration of [^3^H]-spiperone calculated from measurements of added radioactivity. Equation (1) was fitted to the data.

(1) $$y=\frac{B_{MAX}\;*\;x}{K_{D}+x}$$

where *y* is the specific binding at free concentration *x*. *K_D_* values are expressed as µM and *B_MAX_* values as pmol of binding sites per mg of membrane protein.

##### Competition Binding

The binding of tested agonists was determined in competition experiments with 180 pM [^3^H]-Spiperone fitting of Equation (2)

(2) $$y=100-\frac{100\;*\;x}{{x\;+\;\mathrm{IC}}_{50}}$$

where y is the specific radioligand biding at concentration x of competitor expressed as a percent of binding in the absence of a competitor, IC_50_ is the concentration causing 50% inhibition of radioligand binding. Inhibition constants *K_I_* for analyzed agonists were calculated as:

(3) $$K_{I}=\frac{{\mathrm{IC}}_{50}}{1+\frac{\left[D\right]}{K_{D}}}$$

where IC_50_ is the concentration causing 50% inhibition of [^3^H]-spiperone binding calculated according to Equation (2) from competition binding data, [*D*] is the concentration of [^3^H]NMS used, and *K_D_* is its equilibrium dissociation constant calculated according to Equation (1) from saturation binding data.

#### 2.4.4. MTT Assay

Standard MTT (3-[4,5-dimethylthiazol-2-yl]-2,5-diphenyltetrazolium bromide) assay (Sigma Aldrich, Prague, Czech Republic) was used according to the manufacturer´s protocol on the CHO-K1 (Chinese hamster ovary, ECACC, Salisbury, UK) in order to compare the effect of different compounds within the series. The cells were cultured according to ECACC recommended conditions and seeded in a density of 8000 per well as described previously [52]. Briefly, tested compounds were dissolved in DMSO (Sigma Aldrich, Prague, Czech Republic) and subsequently in the growth medium (F–12) so that the final concentration of DMSO did not exceed 1% (*v*/*v*). Cells were exposed to a tested compound for 24 h. Then the medium was replaced by a medium containing 10 μM of MTT and cells were allowed to produce formazan for another approximately 3 h under surveillance. Thereafter, medium with MTT was sucked out and crystals of formazan were dissolved in DMSO (100 µL). Cell viability was assessed spectrophotometrically by the amount of formazan produced. Absorbance was measured at 570 nm with 650 nm reference wavelength on Synergy HT (BioTek, Winooski, VT, USA). IC_50_ was then calculated from the control-subtracted triplicates using non-linear regression (four parameters) of GraphPad Prism 9 software. Final IC_50_ and SEM values were obtained as a mean of three independent measurements.

#### 2.4.5. PAMPA Assay

PAMPA is a high-throughput screening tool applicable for prediction of the passive transport of potential drugs across the BBB [53]. In this study, it has been used as a non-cell-based in vitro assay carried out in a coated 96-well membrane filter. The filter membrane of the donor plate was coated with PBL (Polar Brain Lipid, Avanti, AL, USA) in dodecane (4 µL of 20 mg/mL PBL in dodecane) and the acceptor well was filled with 300 µL of PBS pH 7.4 buffer (*V_A_* tested compounds were dissolved first in the DMSO/phosphate-buffered saline mixture (maximum 0.5% *v/v* of DMSO) and subsequently diluted with phosphate-buffered saline (pH 7.4) to final concentrations of 40–100 μM in the donor wells). Concentration of DMSO did not exceed 0.5% (*v*/*v*) in the donor solution. About 300 µL of the donor solution was added to the donor wells (V_D_) and the donor filter plate was carefully put on the acceptor plate so that coated membrane was “in touch” with both donor solution and acceptor buffer. Test compound diffused from the donor well through the lipid membrane (Area = 0.28 cm^2^) to the acceptor well. The concentration of the drug in both donor and the acceptor wells were assessed after 3, 4, 5, and 6 h of incubation in quadruplicate using the UV plate reader Synergy HT (Biotek, Winooski, VT, USA) at the maximum absorption wavelength of each compound. Besides that, solution of theoretical compound concentration, simulating the equilibrium state established if the membrane were ideally permeable was prepared and assessed as well. Concentration of the compounds in the donor and acceptor well and equilibrium concentration were calculated from the standard curve and expressed as the permeability (*P_e_*) according to the equation:

(4) $$P_{e}=C\times ln\left(1-\frac{\left[drug\right]\;acceptor}{\left[drug\right]\;equilibrium}\right)\;where\;C=\frac{\left(V_{D}\times V_{A}\right)}{\left(V_{D}\times V_{A}\right)\;Area\times Time}$$

## 3. Results

### 3.1. Design of Novel Compounds

Aripiprazole (Figure 1), a partial D_2_Rs agonist, belongs to the third generation of antipsychotic drugs and has been approved by the Food and Drug Administration (FDA) agency for the use as an adjunctive medication in the treatment of depressive and bipolar disorders [9,11,54,55,56]. Aripiprazole has a unique, biased, mode of action comprising partial agonism for Gα_i/o_ and a robust antagonism for Gβγ signaling and an antagonism or a partial agonism for β-arrestin-2 signaling [57,58]. Furthermore, if extracellular concentration of dopamine levels are high (e.g., in mesolimbic areas), aripiprazole competes with dopamine and acts as a partial antagonist. On the other hand, in the presence of low dopamine concentration (e.g., dopamine areas that are involved in working memory), aripiprazole can activate other receptors. Thus, aripiprazole can be classified as a “dopamine stabilizer” [9,59,60]. In addition, aripiprazole is a D_3_ and 5-HT_1A_ receptors partial agonist and 5-HT_2A_Rs antagonist [54,61]. In its structure, aripiprazole combines 3,4-dihydro-7-hydroxyquinolin-2(1*H*)-one fragment attached at position 7- to 2,3-dichlorophenyl piperazine and thus is a member of large group of antipsychotics, so called 1,4-disubstituted arylpiperazines. The biological activity of this subgroup of compounds is encoded by an aromatic warhead, which controls intrinsic activity, and an amine moiety, which is responsible for the formation of a hydrogen bond to the crucial residue Asp^3.32^ in the transmembrane helix 3 of D_2_R [62]. A linker controls subtype selectivity; 3-methylene linker was found suitable for D_2_R selectivity [63,64]. Aromatic/heteroaromatic appendage on the opposite site of the ligand orchestrates receptor affinity [62]. In the past decade, many compounds have been generated containing 2,3-dichlorophenylpiperazine fragment with unique pharmacological profile exhibiting high D_2_Rs affinity. From the extensive SAR, it was deduced that the central linker has only moderate impact on affinity but huge effect on functional activity at D_2_Rs [65,66,67,68]. Besides various substitutions made to the central linker, it has been shown that lipophilic appendages strongly influence functional and subtype selectivity [68,69,70]. 3,4-Dihydroquinolin-2(1*H*)-one scaffold-containing ligands have shown to possess an affinity to D_2_Rs as well. In this case, the nature of the central linker showed a moderate impact on D_2_Rs affinity [71]. Modifications within the amine moiety influenced D_2_Rs affinity and functional selectivity [72]. Besides, substitutions in aromatic warheads also strongly affected D_2_Rs affinity and functional and subtype selectivity [71,72]. Recently, some 3,4-dihydroquinolin-2-(1*H*)-one scaffold-containing compounds exhibited high D_4_Rs selectivity over other D_2_-like family receptors [73]. These findings show that small structural modifications within one region of the molecule based on aripiprazole can tune significantly the properties of the ligand.

In the study of Lopéz et al., researchers tethered arylpiperazine-like core (different phenylpiperazine moieties) with 3,4-dihydroquinolin-2(1*H*)-one moiety (red color, Figure 1) at position 1- [41]. USC-D301 (Figure 1) is an example of small molecule exhibiting strong D_2_R antagonism and high selectivity over D_3_Rs [41]. On the other hand, eticlopride (Figure 1) is a substituted benzamide analog without 1,4-disubstituted arylpiperazine fragment exhibiting very high affinity for D_2_Rs [74]. Thus, we wanted to explore the effect of novel prepared analogs containing various tertiary amines with 3,4-dihydroquinolin-2(1*H*)-one fragment connected by aliphatic linker at position 1- of the quinolinone core towards the D_2_Rs as the main targets. We are aware of the fact that other dopamine receptors (especially D_3_R and D_4_R) are of importance for the complexity of the antipsychotic action, as observed also in case of aripiprazole [54], however, this was not the aim of this study. Molecular imaging studies have revealed that striatal D_2_Rs antagonism is essential in vivo for therapeutic doses of all neuroleptics [19,75,76,77,78,79,80], and D_2_Rs affinity of antipsychotic drugs is the crucial for their antipsychotic efficacy. The aliphatic linkers (1,3-propane-diyl and 1,4-butane-diyl) for new ligands were chosen based on previous studies [41,44]. These new derivatives were evaluated for their D_2_R antagonistic properties with the emphasis on the structure–activity relationships regarding the type of amine and length of the linker.

### 3.2. The Synthesis of Novel Compounds

**Scheme 1:**
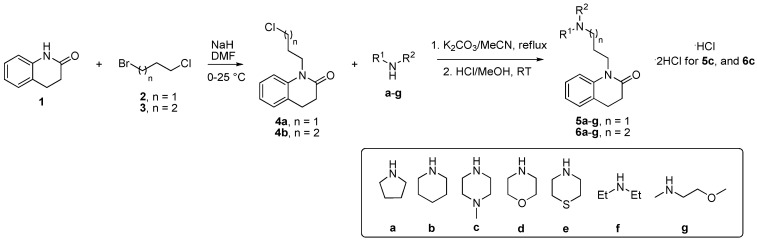
Synthesis of new quinolinone derivatives **5a**-**g** and **6a**-**g**.

The syntheses of novel analogues are depicted in Scheme 1. Nucleophilic addition of 1-bromo-3-chloropropane (**2**) or 1-bromo-4-chlorobutane (**3**) to the starting compound 3,4-dihydro-2(1*H*)-quinolinone (**1**, Scheme 1) in the presence of sodium hydride produced intermediates **4a**,**b** [42]. These derivatives were obtained in excellent yields (>70%). The final compounds **5a**-**g** and **6a**-**g** were prepared again by nucleophilic addition of the appropriate amine (**a**-**g**) to **4a**,**b** in the presence of K_2_CO_3_ [46]. All the reactions exhibited good yields (>45%). The final products were analyzed by ^1^H and ^13^C NMR, HRMS and LC-MS analysis revealing the purity > 95%.

### 3.3. Binding Affinities of Novel Compounds at D_2_Rs and Their Cytotoxicities

The results of the affinity of **5a**-**g** and **6a**-**g** for D_2_R are summarized in Table 1. In general, 1,3-propane-diyl derivatives **5a**-**g** exhibited slightly better D_2_R antagonism than their 1,4-butane-diyl counterparts **6a**-**g**. The aliphatic analogues **5f**,**g** and **6g** possessed slightly lower D_2_R antagonism than the molecules with cyclic amines **5a**-**e** and **6a**-**c**,**e**. Morpholine-containing compound **6d** showed the lowest D_2_R antagonism from all cyclic amine derivatives (**5a**-**e** and **6a**-**e**). On the other hand, thiomorpholine analogue **5e** had the strongest antagonistic behavior at D_2_Rs from all prepared final compounds. Interestingly, the final derivatives **5a**-**g**, **6a**-**g** exhibited very low cytotoxicity in MTT (3-[4,5-dimethylthiazol-2-yl]-2,5-diphenyltetrazolium bromide) assay using Chinese hamster ovary cell lines (Table 1) so that the relatively lower affinity toward D_2_R is compensated by low toxicity allowing higher dosing.

### 3.4. Molecular Modelling Studies

**Figure 2:**
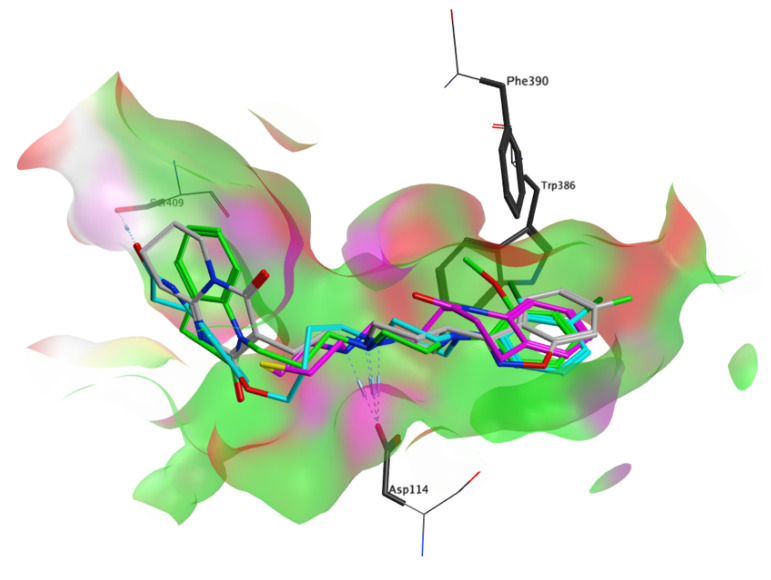
All four ligands with docked poses in the binding pocket with different rendering style (grey: risperidone, cyan: aripiprazole, green: USC-D301, purple: **5e**). Surface color of pocket: green is hydrophobic, purple is polar, and red is exposed. For clarity, only residues that showed selective interactions in the docking screens are rendered, sidechains in bold.

**Figure 3:**
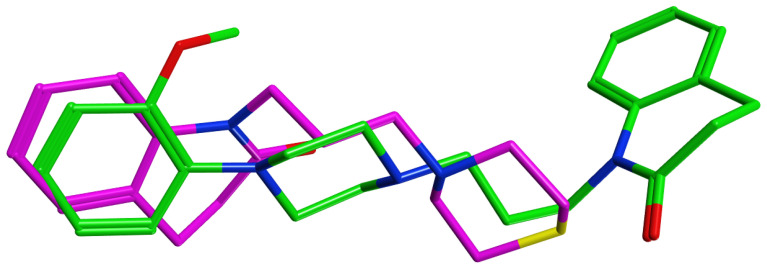
Closer view of the 3D overlay of the final docked conformations of USC-D301 (green) and **5e** (purple). Despite their chemical similarity, there is no alignment.

A molecular modelling study was undertaken to help explain differences in binding strength. Quantitative results are summarized in Table 2, the docking score (S) and the calculated relative binding energy (ddG) from the thermodynamic integration free energy calculation follow the same tendency as the measured affinities. Docking of USC-D301 and **5e** revealed strongly differing binding poses, despite their high molecular similarity (Figure 2). The positively charged nitrogen of all docked ligands was recognized by Asp114 via a strong ionic ammonium-carboxylate interaction. A pi–pi interaction with Phe390 was also apparent, with the isoxazole, 2,3-dichlorophenyl and 2-methoxyphenyl fragment of risperidone, aripiprazole, and USC-D301, respectively. Surprisingly, the lowest scoring docking pose of **5e** did not overlap with USC-D301 (Figure 3). Owing to its smaller size and thiomorpholine cap, there is no aromatic group at that position that can undergo the stacking interaction with Phe390 or other nearby residues, likely leading to a strong penalty to the binding energy. Instead, the molecule adopts a flipped conformation that places the benzolactam fragment in this pocket, where the fused benzene ring is not positioned well to undergo stacking with Phe390, Trp386, and others.

### 3.5. Central Nervous System Availability Prediction and Study for Novel Compounds

Before the synthesis, we have calculated the so-called BBB score to predict the compound’s CNS availability. Indeed, all the compounds displayed high values above 5.0 (5.2–5.4) which is indicative of their high potential to cross BBB. The prediction was then confirmed by the data from parallel artificial membrane permeation (PAMPA) assay pointing out their potential to cross the BBB by passive diffusion (**5a**-**g** and **6a**-**g** *P*_e_ (× 10^−6^ cm s^−1^) = 7.0–24) (Table 3). The validation of PAMPA has been performed using standard compounds whose availability or unavailability was experimentally predicted in vitro and confirmed in vivo [53,82].

## 4. Conclusions

In summary, a series of 3,4-dihydroquinolin-2(1*H*)-one analogues, inspired by aripiprazol was designed and synthesized. The substitutions of the amine group revealed a negligible impact on D_2_R affinity. Although the binding affinities at D_2_Rs of new analogues are much weaker compared to aripiprazole, they are very close to the binding affinity, for instance, of memantine acting as *N*-methyl-d-aspartate receptor antagonist, a well-established drug for the treatment of Alzheimer’s disease [83,84]. Out of these ligands, **5e** possessed the highest D_2_R affinity, very low cytotoxicity profile, and the highest probability to cross the BBB. Molecular modeling simulation revealed completely different binding mode of **5e** compared to USC-D301, which might be the culprit of the reduced affinity of **5e** toward D_2_R. The subject of further investigation of these compounds will be to assess their affinity toward other members of the D_2_-like receptors family and other GPCRs, especially 5-HT_1A_ and 5-HT_2A_Rs. Since aripiprazole has few of the typical adverse effects of other antipsychotics, such as extrapyramidal symptoms, hyperprolactinemia, weight gain, metabolic disorders, and sedation given to its unique biased activity and/or partial agonistic/antagonistic actions on D_2_Rs supplemented by the action on other dopamine receptor subtypes (mainly D_3_R) and 5-HT receptor subtypes (mainly 5-HT_1A_, 5-HT_2A_) [54], the ongoing study must determine the functional affinity of newly developed compounds at these receptors to evaluate the real antipsychotic effect and clinical potential [85].

## Figures and Tables

**Table 1:** Binding affinity of tested final compounds **5a**-**g** and **6a**-**g** at D_2_Rs and their cytotoxicity.

Compound	*K*_i_ (μM) ± SEM ^1^	CHO-K1 IC_50_ (mM) ± SEM ^2^
**5a**	24 ± 5.8	2.0 ± 0.8
**5b**	9.7 ± 1.6	1.5 ± 0.3
**5c**	12 ± 3.2	2.0 ± 1.0
**5d**	20 ± 1.6	1.1 ± 0.3
**5e**	7.6 ± 1.9	0.5 ± 0.1
**5f**	37 ± 8.6	0.9 ± 0.2
**5g**	26 ± 6.1	1.5 ± 0.5
**6a**	23 ± 3.6	1.1 ± 0.2
**6b**	14 ± 2.5	0.8 ± 0.2
**6c**	21 ± 0.6	1.2 ± 0.2
**6d**	41 ± 14	1.3 ± 0.2
**6e**	9.7 ± 2.3	0.8 ± 0.1
**6f**	27 ± 4.7	1.8 ± 0.3
**6g**	45 ± 11	2.1 ± 0.8
Aripiprazole	3.3 nM ^3^	0.1 ^4^

^1^ Values are expressed as mean ± SEM (μM) (*n* = 3). ^2^ The effect of the compounds on the cell viability. Values are expressed as the IC_50_: mean ± SEM (mM) (*n* = 3). ^3^ Please see paper by Shapiro et al. [54]. The value is *K*_i_ value. ^4^ The effect of aripiprazole in MTT assay on NIH3T3 cell lines. The IC_50_ value taken from ref. [81]. K_D_ = 60 ± 2.8 pM (*n* = 6); B_MAX_ = 2.0 ± 0.1 pmol/mg (*n* = 6).

**Table 2:** Results for molecular studies.

Ligand	S (Docking Score, kcal/moL)	ddG (Relative Free Energy, kcal/moL)
risperidone	−9.6461	−10.0988
aripiprazole	−9.1619	−10.2544
USC-D301	−8.4562	−9.5903
**5e**	−7.0336	−8.2686

**Table 3:** Prediction of BBB barrier penetration of the studied compounds expressed as *P*_e_ (*n* = 3) and BBB score of final derivatives.

Compound	BBB Score ^1^	*P*_e_ ± SEM (× 10^−6^ cm s^−1^)	CNS (+/−) ^2^
**5a**	5.3	7.3 ± 0.8	CNS +
**5b**	5.2	13 ± 0.1	CNS +
**5c**	5.4	7.0 ± 0.4	CNS +
**5d**	5.2	12 ± 2.0	CNS +
**5e**	5.2	24 ± 2.1	CNS +
**5f**	5.2	9.4 ± 0.4	CNS +
**5g**	5.3	10 ± 1.6	CNS +
**6a**	5.2	7.7 ± 1.8	CNS +
**6b**	5.2	10 ± 1.4	CNS +
**6c**	5.4	7.1 ± 1.2	CNS +
**6d**	5.3	17 ± 2.1	CNS +
**6e**	5.2	23 ± 3.3	CNS +
**6f**	5.2	7.4 ± 0.9	CNS +
**6g**	5.3	9.5 ± 1.1	CNS +
Donepezil	5.3	22 ± 2.1	CNS +
Tacrine	5.4	6.0 ± 0.6	CNS +
Rivastigmine	5.1	20 ± 2.1	CNS +
Furosemide	-	0.2 ± 0.1	CNS −
Chlorothiazide	-	1.2 ± 0.5	CNS −
Ranitidine	-	0.4 ± 0.3	CNS −

^1^ Ligands exhibiting BBB score of 4–5 have 54.5% probability to cross BBB; score ranging between 5–6 indicates 90.3% probability of potential central activity [49]; ^2^ CNS + (high BBB permeability predicted), *P*_e_ (10^−6^ cm∙s^−1^) > 4.0; CNS − (low BBB permeability predicted), *P*_e_ (10^−6^ cm∙s^−1^) < 2.0; CNS +/− (BBB permeability uncertain), *P*_e_ (10^−6^ cm∙s^−1^) from 4.0 to 2.0 [53].

## Data Availability

The compounds developed within this study are available from the corresponding author on reasonable request.

## Supplementary Materials

The following are available online at https://www.mdpi.com/article/10.3390/biom11091262/s1. Figure S1: ^1^H NMR spectrum for **5a**. Figure S2: ^13^C NMR spectrum for **5a**. Figure S3: UV-LC chromatogram for **5a**. Figure S4: HRMS spectrum for **5a**. Figure S5: ^1^H NMR spectrum for **5b**. Figure S6: ^13^C NMR spectrum for **5b**. Figure S7: UV-LC chromatogram for **5b**. Figure S8: HRMS spectrum for **5b.** Figure S9: ^1^H NMR spectrum for **5c**. Figure S10: ^13^C NMR spectrum for **5c**. Figure S11: UV-LC chromatogram for **5c**. Figure S12: HRMS spectrum for **5c.** Figure S13: ^1^H NMR spectrum for **5d**. Figure S14: ^13^C NMR spectrum for **5d**. Figure S15: UV-LC chromatogram for **5d**. Figure S16: HRMS spectrum for **5d**. Figure S17: ^1^H NMR spectrum for **5e**. Figure S18: ^13^C NMR spectrum for **5e**. Figure S19: UV-LC chromatogram for **5e**. Figure S20: HRMS spectrum for **5e**. Figure S21: ^1^H NMR spectrum for **5f**. Figure S22: ^13^C NMR spectrum for **5f**. Figure S23: UV-LC chromatogram for **5f**. Figure S24: HRMS spectrum for **5f**. Figure S25: ^1^H NMR spectrum for **5g**. Figure S26: ^13^C NMR spectrum for **5g**. Figure S27: UV-LC chromatogram for **5g**. Figure S28: HRMS spectrum for **5g**. Figure S29: ^1^H NMR spectrum for **6a**. Figure S30: ^13^C NMR spectrum for **6a**. Figure S31: UV-LC chromatogram for **6a**. Figure S32: HRMS spectrum for **6a**. Figure S33: ^1^H NMR spectrum for **6b**. Figure S34: ^13^C NMR spectrum for **6b**. Figure S35: UV-LC chromatogram for **6b**. Figure S36: HRMS spectrum for **6b.** Figure S37: ^1^H NMR spectrum for **6c**. Figure S38: ^13^C NMR spectrum for **6c**. Figure S39: UV-LC chromatogram for **6c**. Figure S40: HRMS spectrum for **6c.** Figure S41: ^1^H NMR spectrum for **6d**. Figure S42: ^13^C NMR spectrum for **6d**. Figure S43: UV-LC chromatogram for **6d**. Figure S44: HRMS spectrum for **6d**. Figure S45: ^1^H NMR spectrum for **6e**. Figure S46: ^13^C NMR spectrum for **6e**. Figure S47: UV-LC chromatogram for **6e**. Figure S48: HRMS spectrum for **6e**. Figure S49: ^1^H NMR spectrum for **6f**. Figure S50: ^13^C NMR spectrum for **6f**. Figure S51: UV-LC chromatogram for **6f**. Figure S52: HRMS spectrum for **6f**. Figure S53: ^1^H NMR spectrum for **6g**. Figure S54: ^13^C NMR spectrum for **6g**. Figure S55: UV-LC chromatogram for **6g**. Figure S56: HRMS spectrum for **6g**. Figure S57: Saturation binding curve and Scatchard plot of the affinity of [^3^H]spiperone to D_2_Rs. Figure S58: Inhibition of [^3^H]spiperone binding to D_2_RS receptors by 3,4-dihydroquinolin-2(1*H*)-one derivatives (**5a**-**g**). Figure S59: Inhibition of [^3^H]spiperone binding to D_2_RS receptors by 3,4-dihydroquinolin-2(1*H*)-one derivatives (**6a**-**g**). Figure S60: Schematic representation of the settings used in the Thermodynamic Integration Free Energy Calculation. Table S1: Functional activities of **5a**-**g** and **6a**-**g** at D_2_Rs.
