# Supplementary material for: Synthesis and In Vitro Evaluation of Novel Dopamine Receptor D2 3,4-dihydroquinolin-2(1H)-one Derivatives Related to Aripiprazole
Source: Biomolecules. 2021 Aug 24;11(9):1262. doi: 10.3390/biom11091262 (PMC8464836; doi:10.3390/biom11091262)
Supplement: Supplementary file 1 [file biomolecules-11-01262-s001.zip › biomolecules-1320999-supplementary.pdf]

Synthesis and *in vitro* evaluation of novel dopamine receptor D_2 3,4-dihydroquinolin-2(1*H*)-one derivatives related to aripiprazole

Radomir Juza[a,b], Kristyna Stefkova[a], Wim Dehaen[c], Alena Randakova[d], Tomas Petrasek[a], Iveta Vojtechova[a], Tereza Kobrlova[e], Lenka Pulkrabkova[e], Lubica Muckova[e], Marko Mecava[e], Lukas Prchal[e], Eva Mezeiova[a,e], Kamil Musilek[b], Ondrej Soukup[a,*], Jan Korabecny[a,e,*]

[a] National Institute of Mental Health, Topolova 748, 250 67 Klecany, Czech Republic

[b] Department of Chemistry, University of Hradec Kralove, Rokitanskeho 62, 500 03 Hradec Kralove, Czech Republic

[c] CZ-OPENSCREEN: National Infrastructure for Chemical Biology, Department of Informatics and Chemistry, Faculty of Chemical Technology, University of Chemistry and Technology Prague, Technicka 5, Dejvice, 166 28 Prague, Czech Republic

[d] Institute of Physiology, Czech Academy of Sciences, Videnska 1083, 142 20, Prague, Czech Republic

[e] Biomedical Research Centre, University Hospital Hradec Kralove, Sokolska 581, 500 05 Hradec Kralove, Czech Republic

* Corresponding authors: Ondrej Soukup, phone: 00-420-495-833-447, e-mail: ondrej.soukup@fnhk.cz; Jan Korabecny, phone: 00-420-973-255-167, e-mail: jan.korabecny@fnhk.cz

1. 1H, 13C NMR spectra and LC-HRMS results for final compounds 5a-g and 6a-g

1.1 1-(3-(Pyrrolidin-1-yl)propyl)-3,4-dihydroquinolin-2(1*H*)-one (5a)

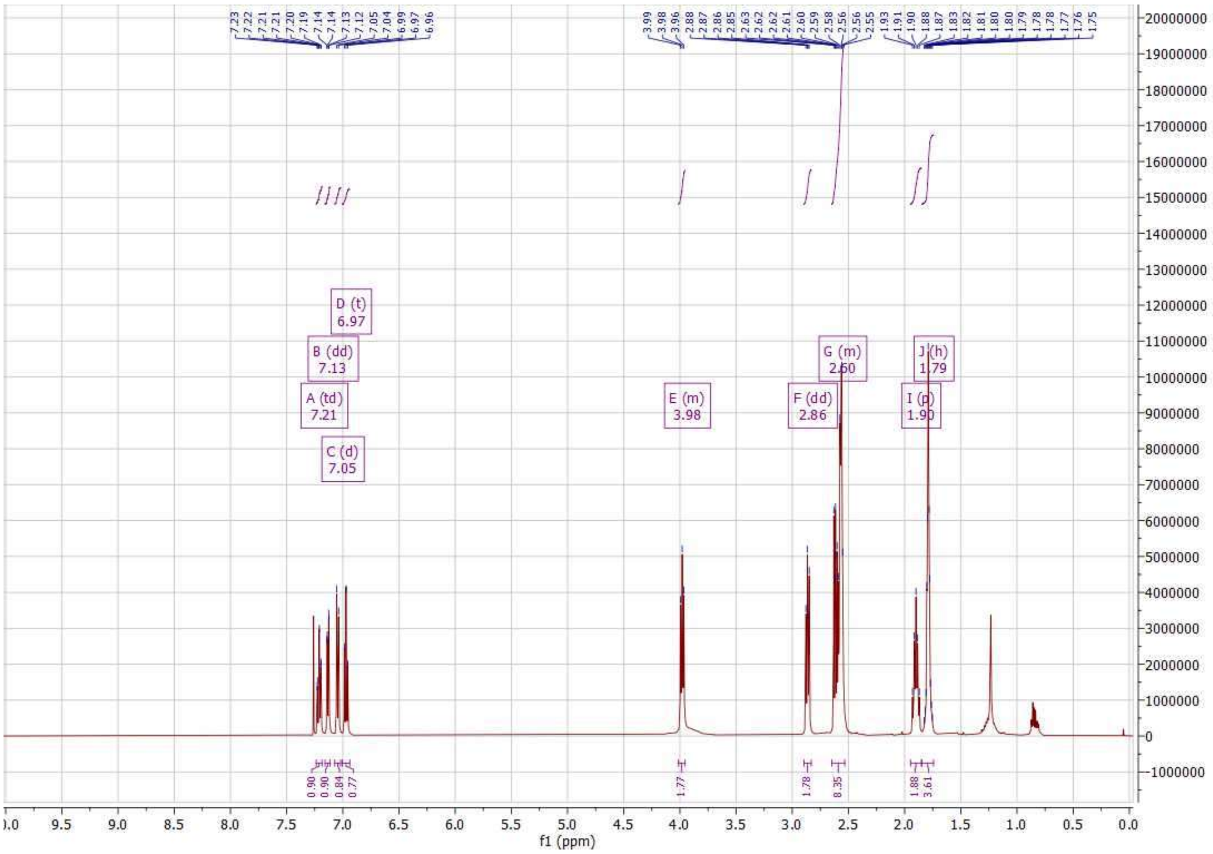

Figure S1 - ^{1}H NMR spectrum of 5a.

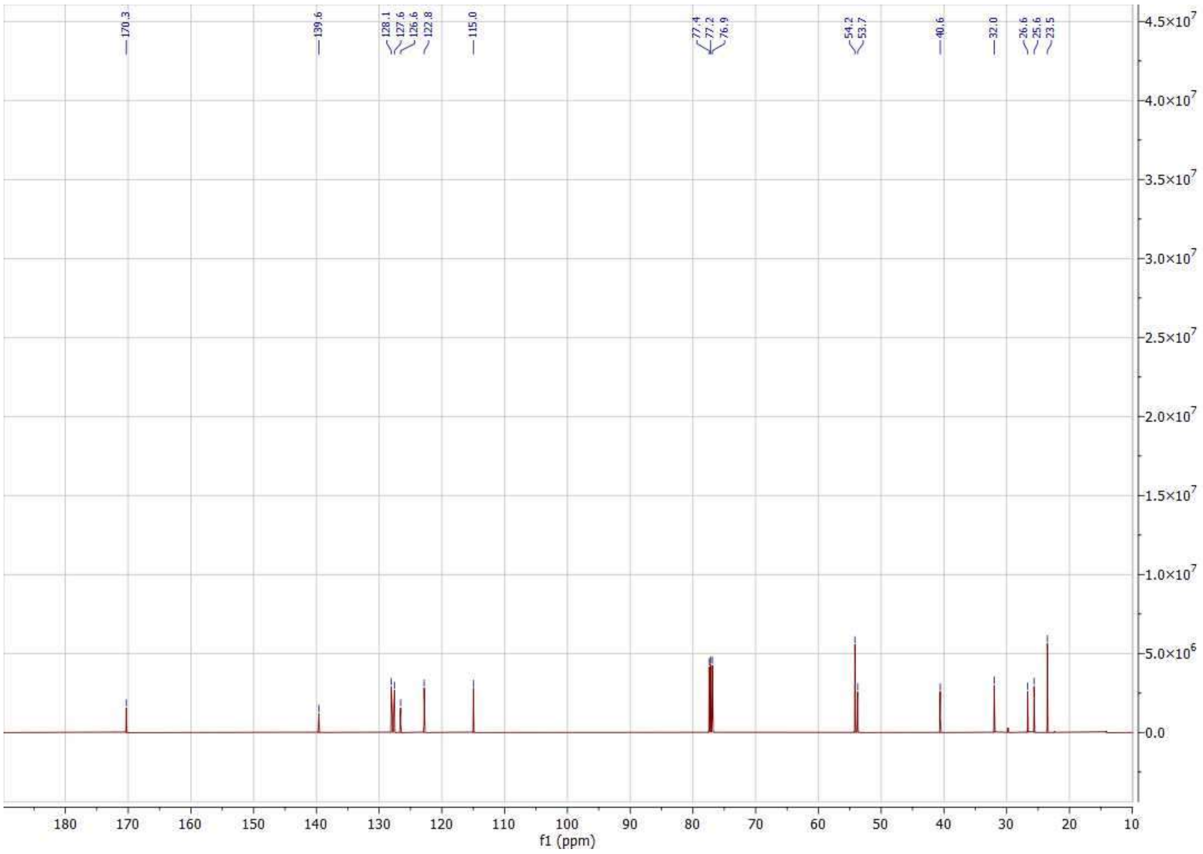

Figure S2 - ^{13}C NMR spectrum of 5a.

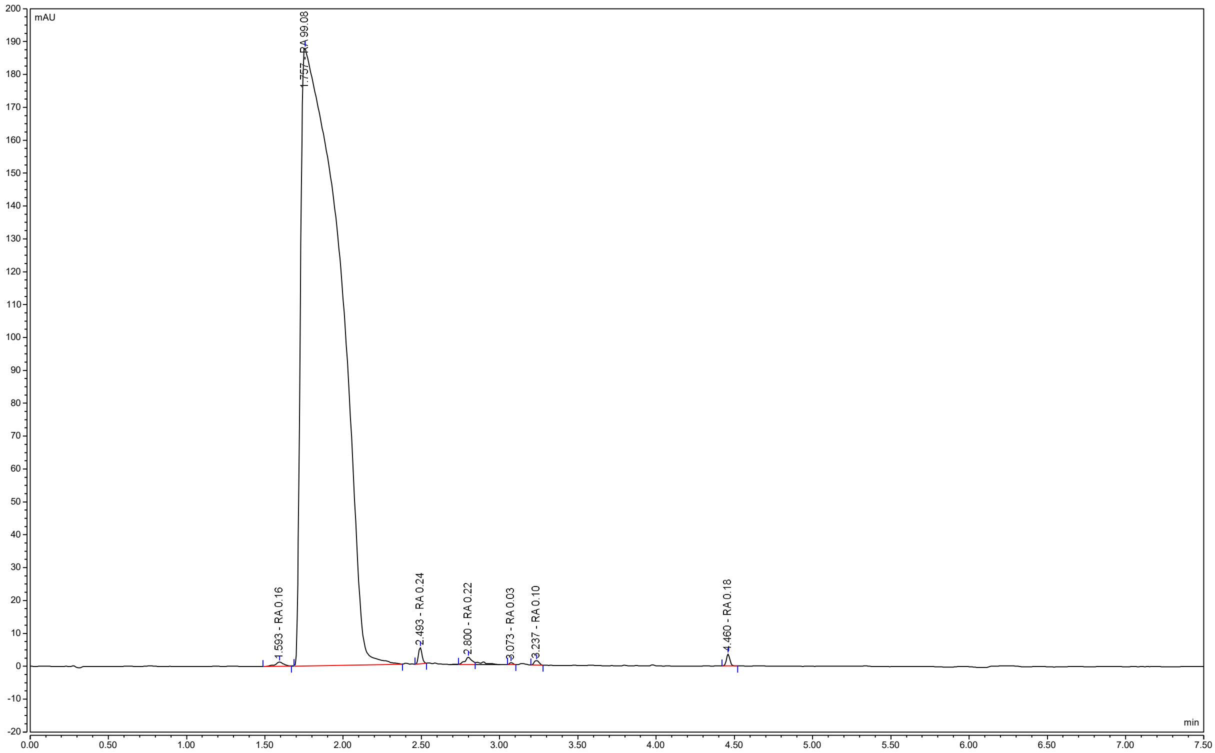

Figure S3 – UV-LC chromatogram of 5a.

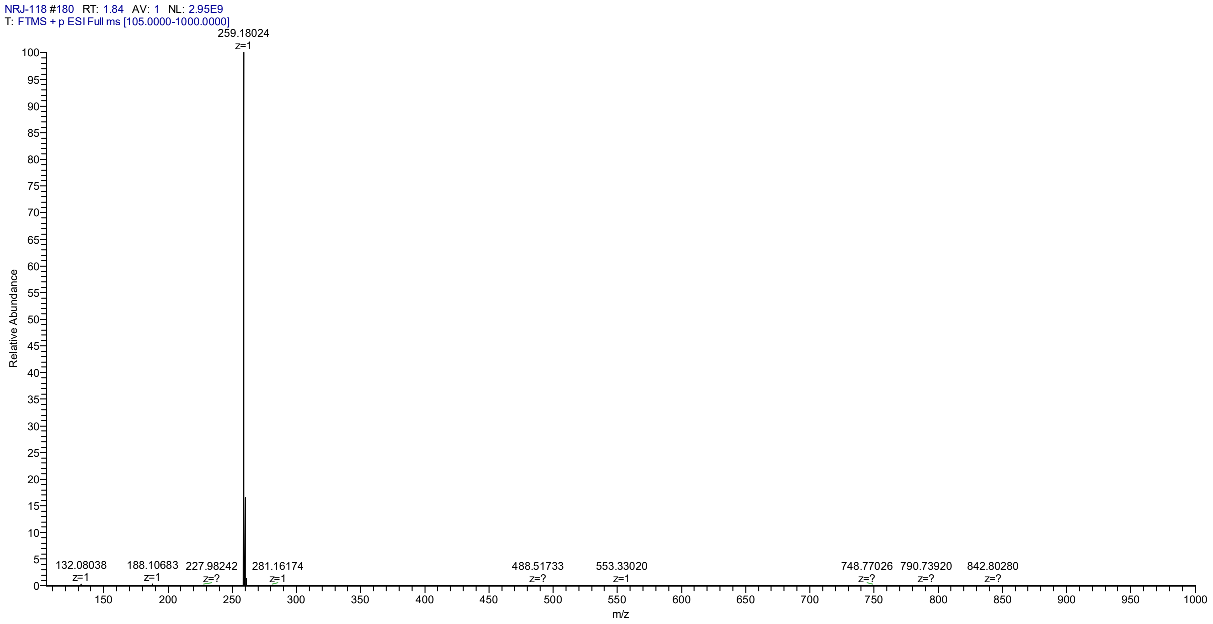

Figure S4 - HRMS spectrum for 5a.

1.2 1-(3-(Piperidin-1-yl)propyl)-3,4-dihydroquinolin-2(1*H*)-one (5b)

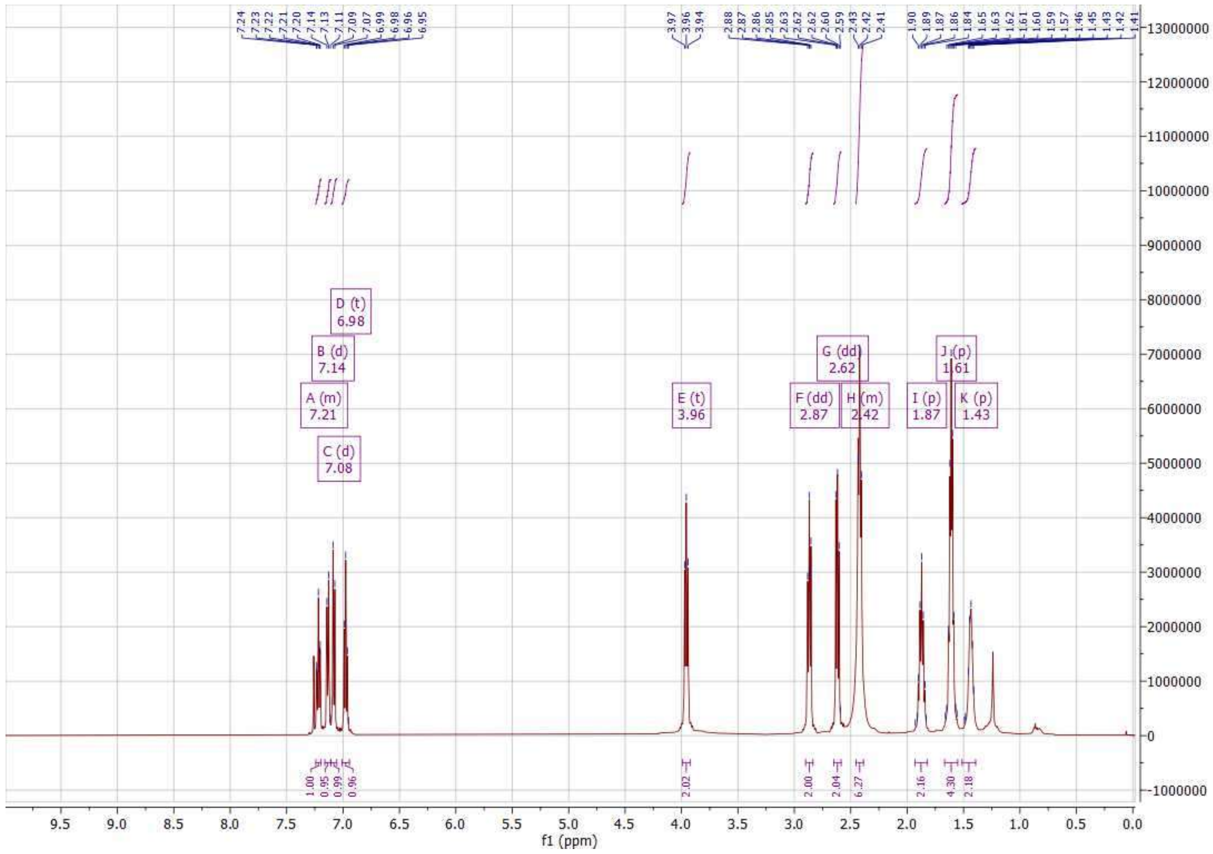

Figure S5 - ^{1}H NMR spectrum for 5b.

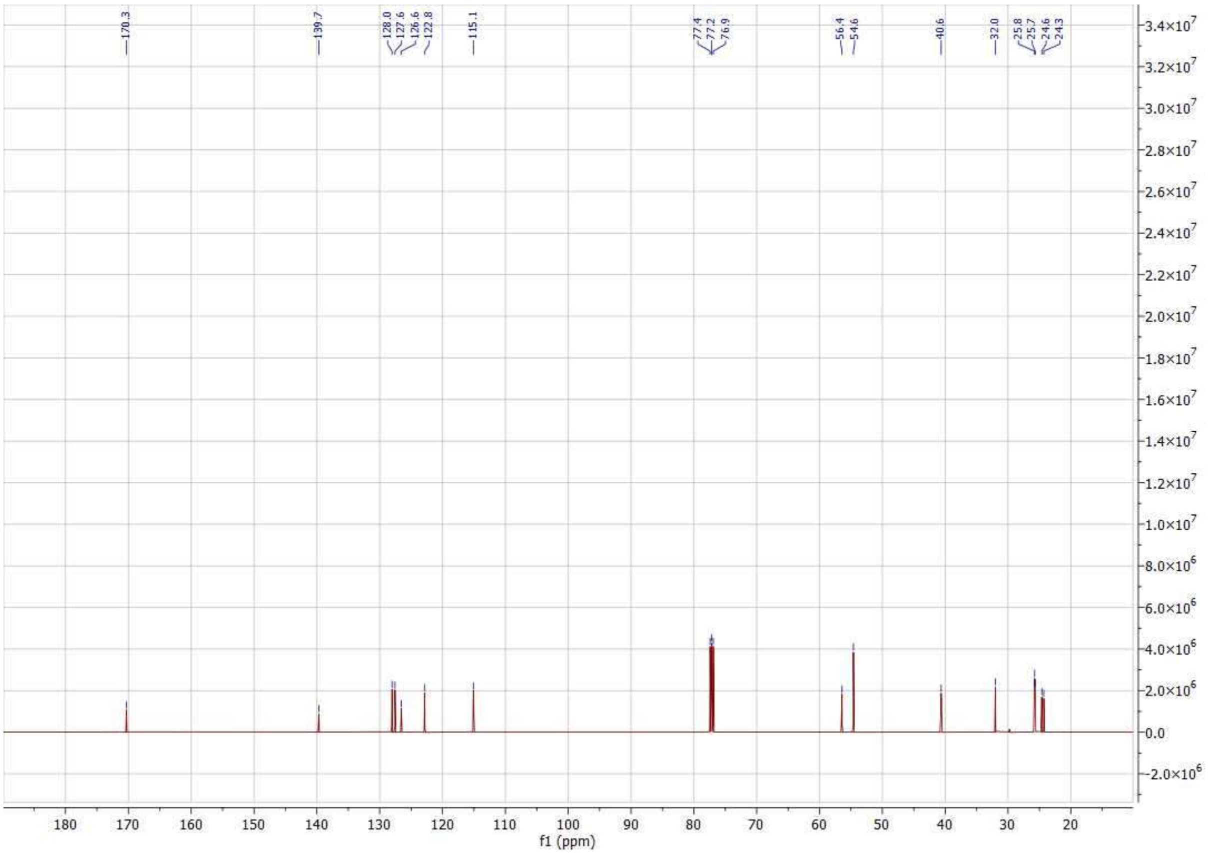

Figure S6 - ^{13}C NMR spectrum for 5b.

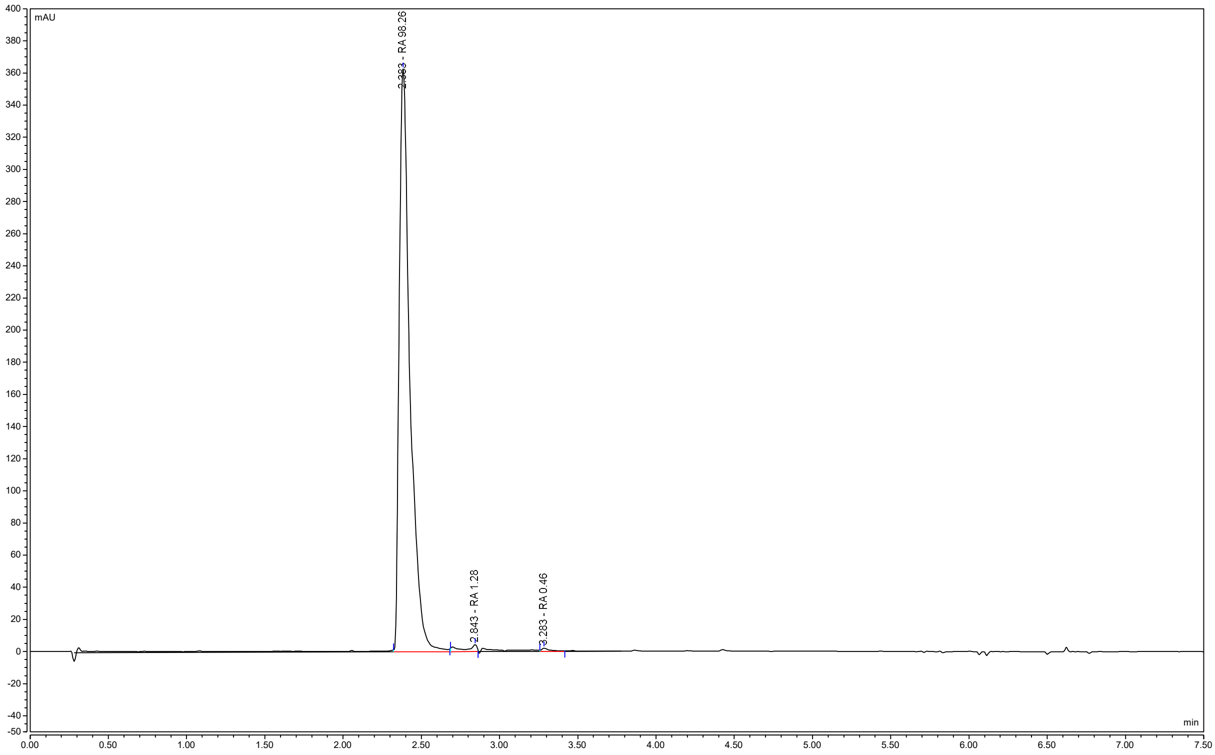

Figure S7 – UV-LC chromatogram for 5b.

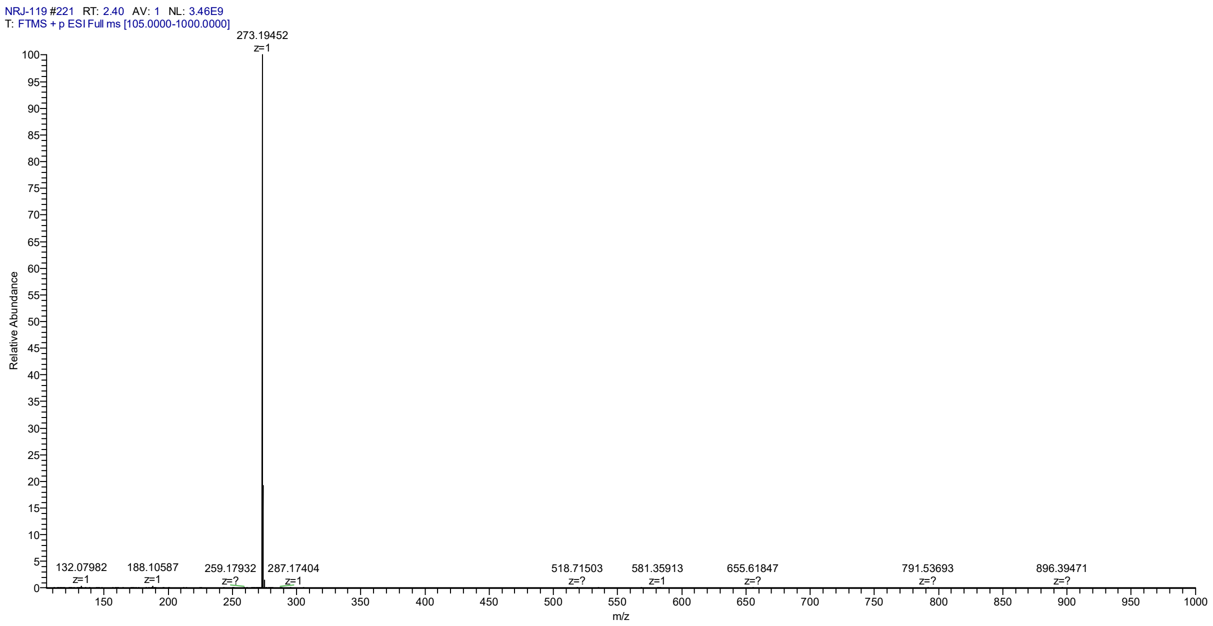

Figure S8 - HRMS spectrum for 5b.

1.3 1-(3-(4-Methylpiperazin-1-yl)propyl)-3,4-dihydroquinolin-2(1*H*)-one (5c)

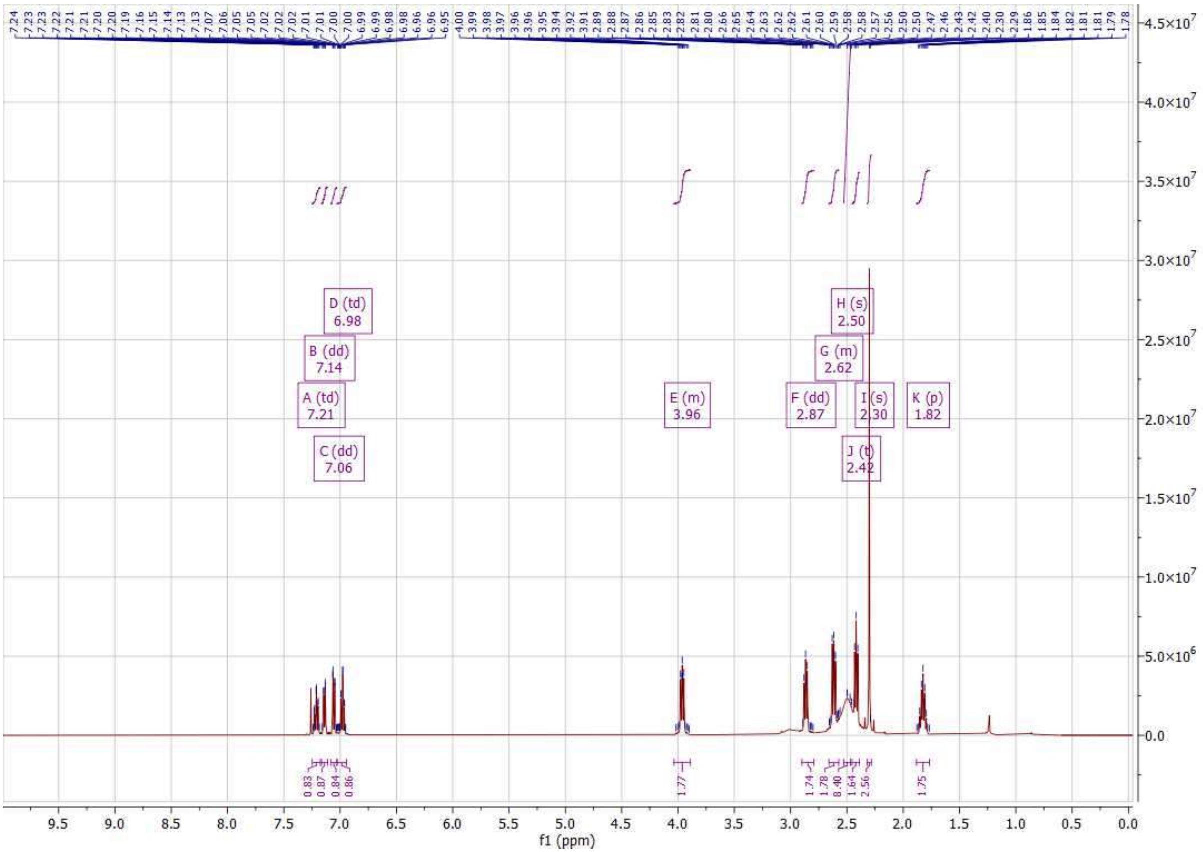

Figure S9 - ^{1}H NMR spectrum for 5c.

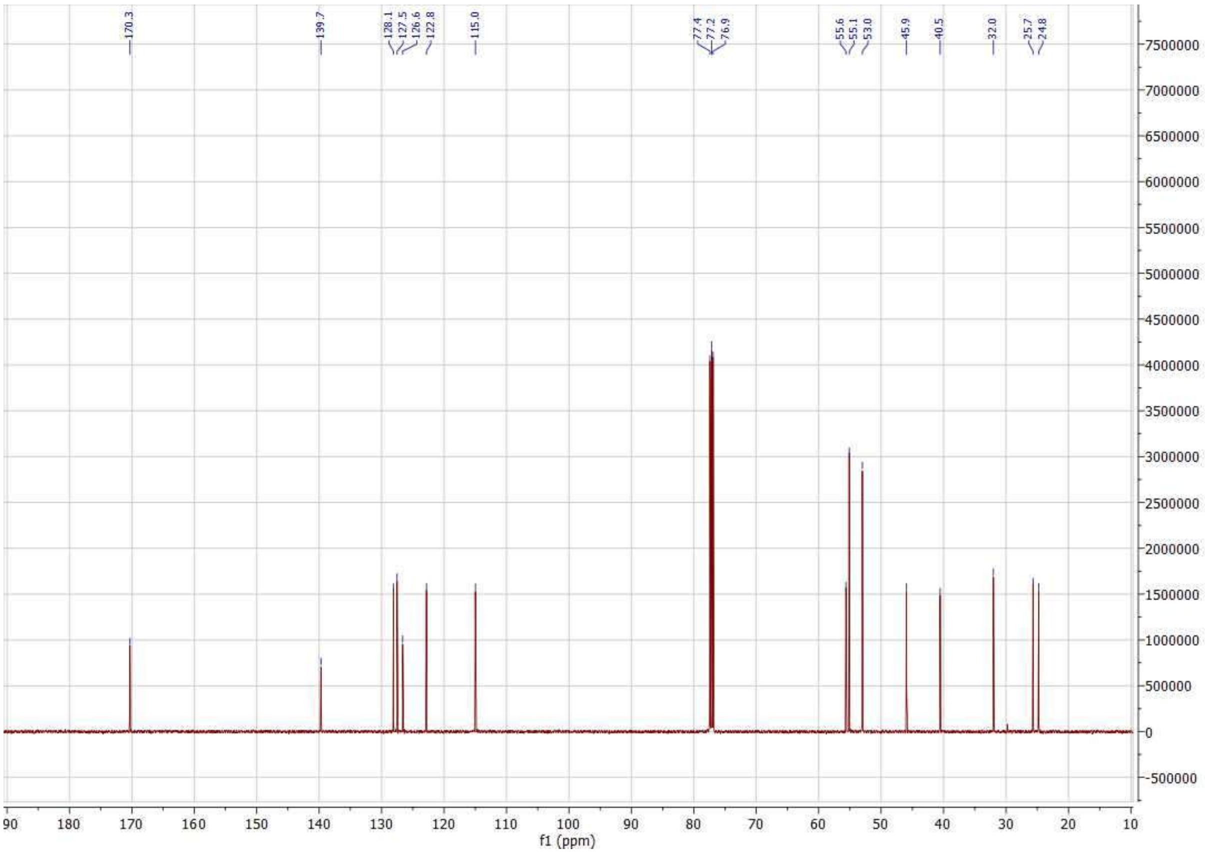

Figure S10 - ^{13}C NMR spectrum for 5c.

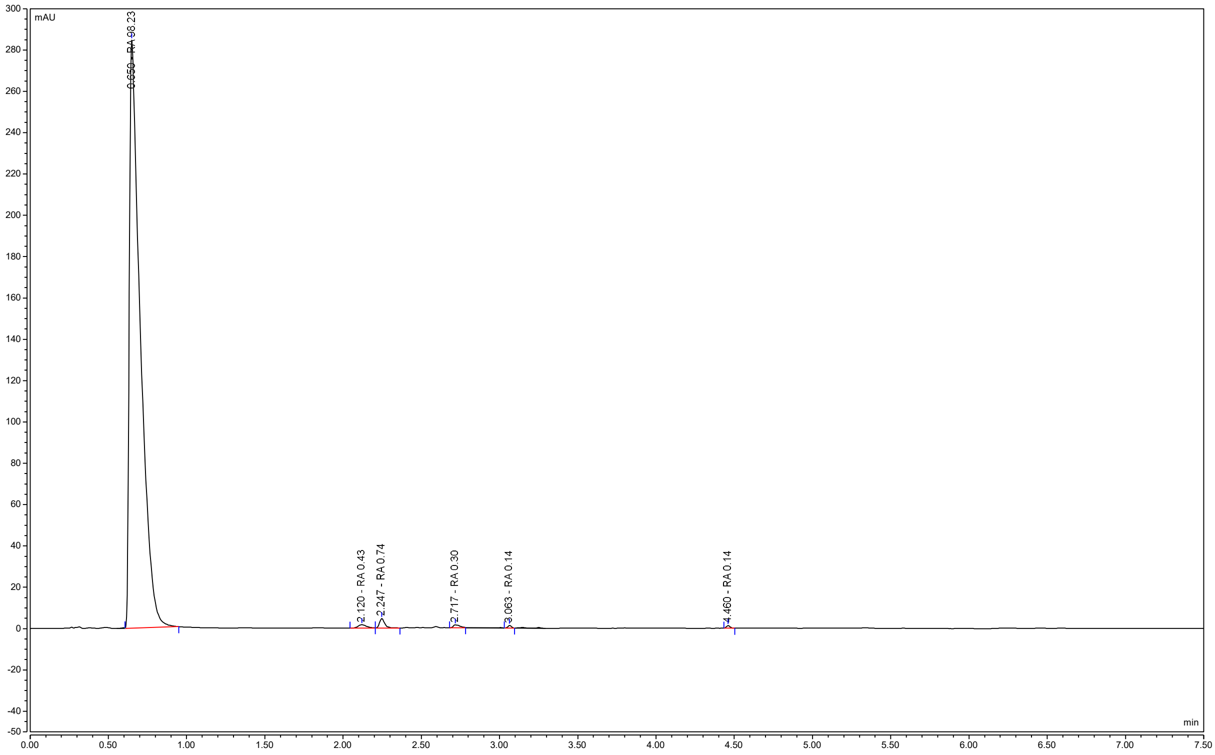

Figure S11 – UV-LC chromatogram for 5c.

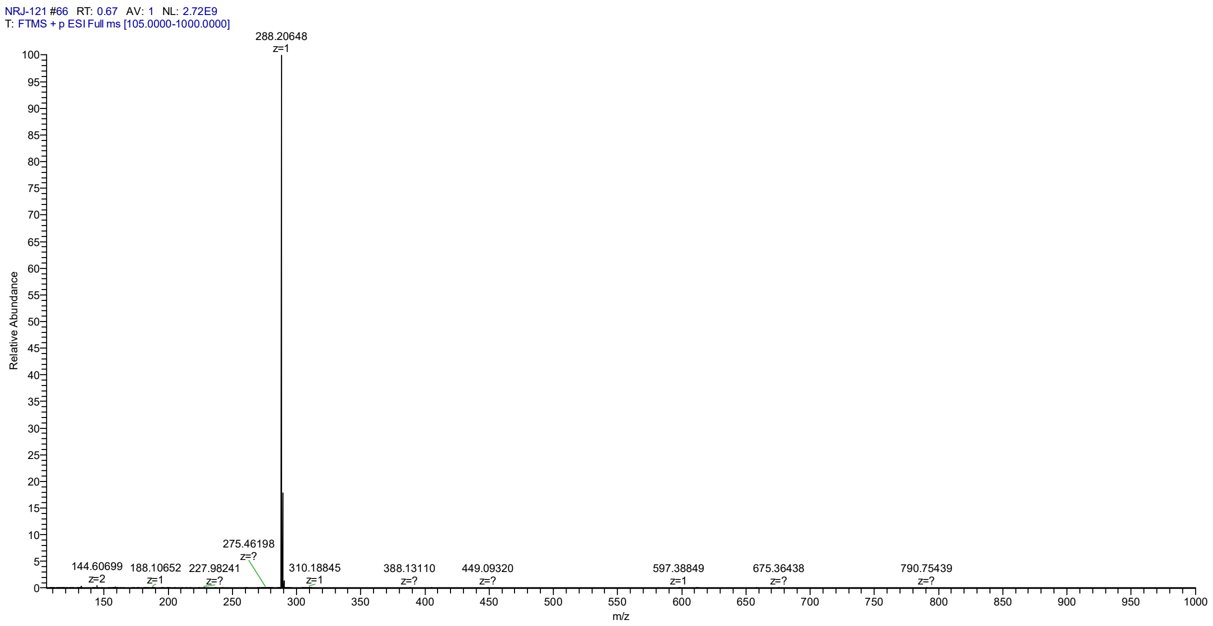

Figure S12 - HRMS spectrum for 5c.

1.4 1-(3-Morpholinopropyl)-3,4-dihydroquinolin-2(1*H*)-one (5d)

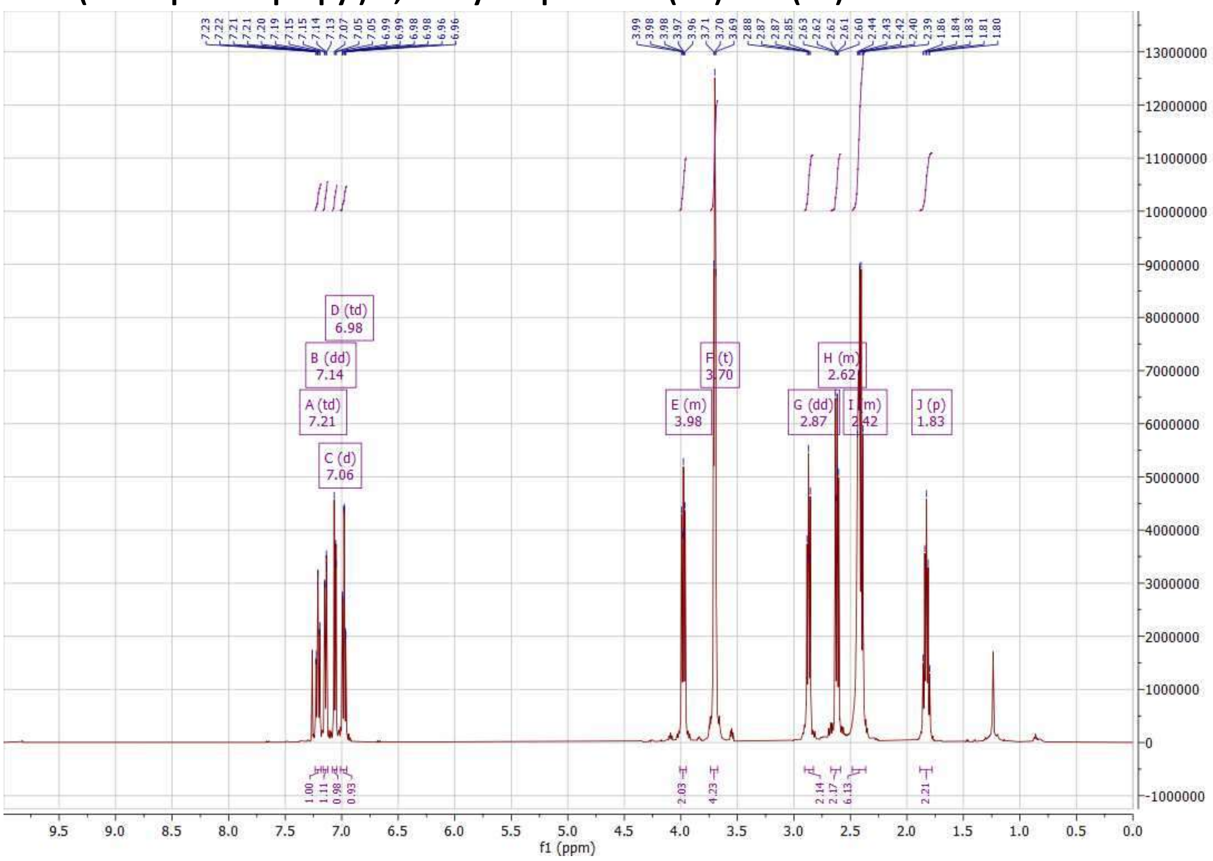

Figure S13 - ^{1}H NMR spectrum for 5d.

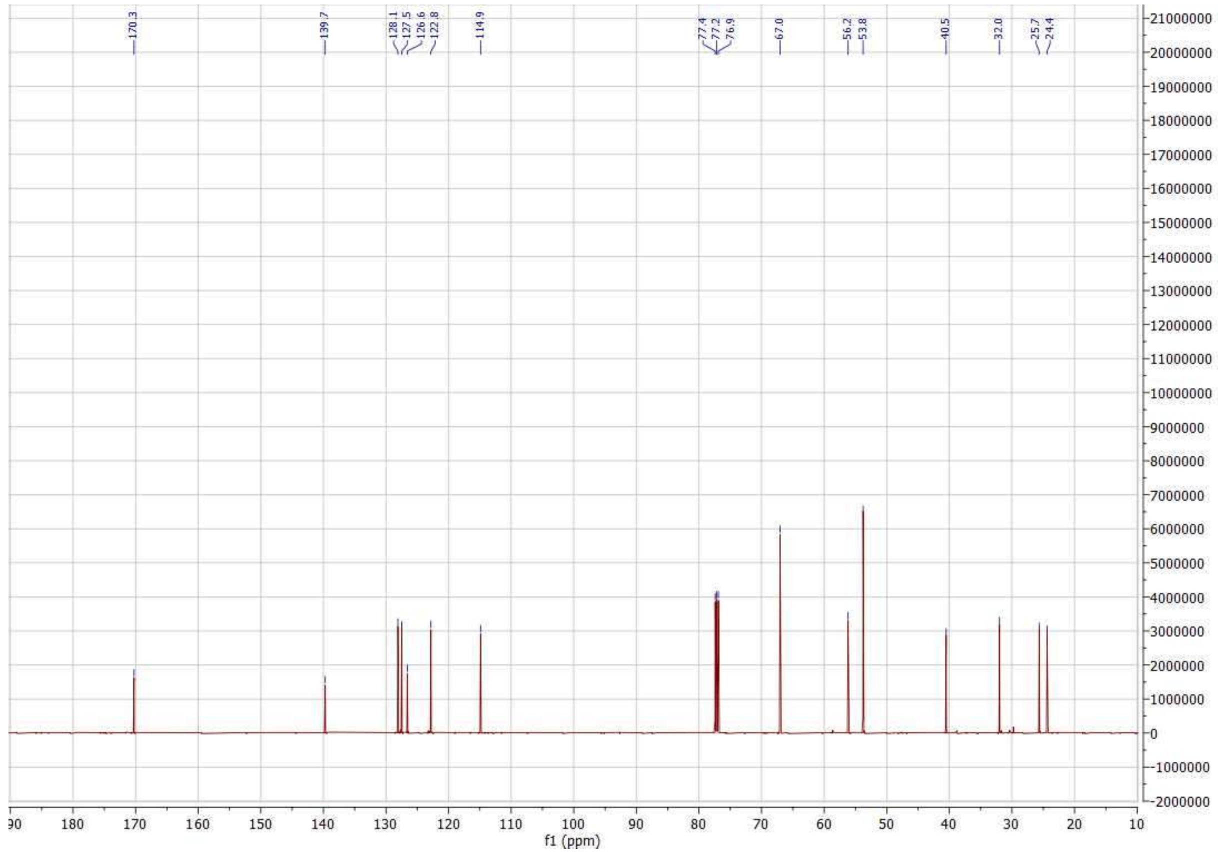

Figure S14 - ^{13}C NMR spectrum for 5d.

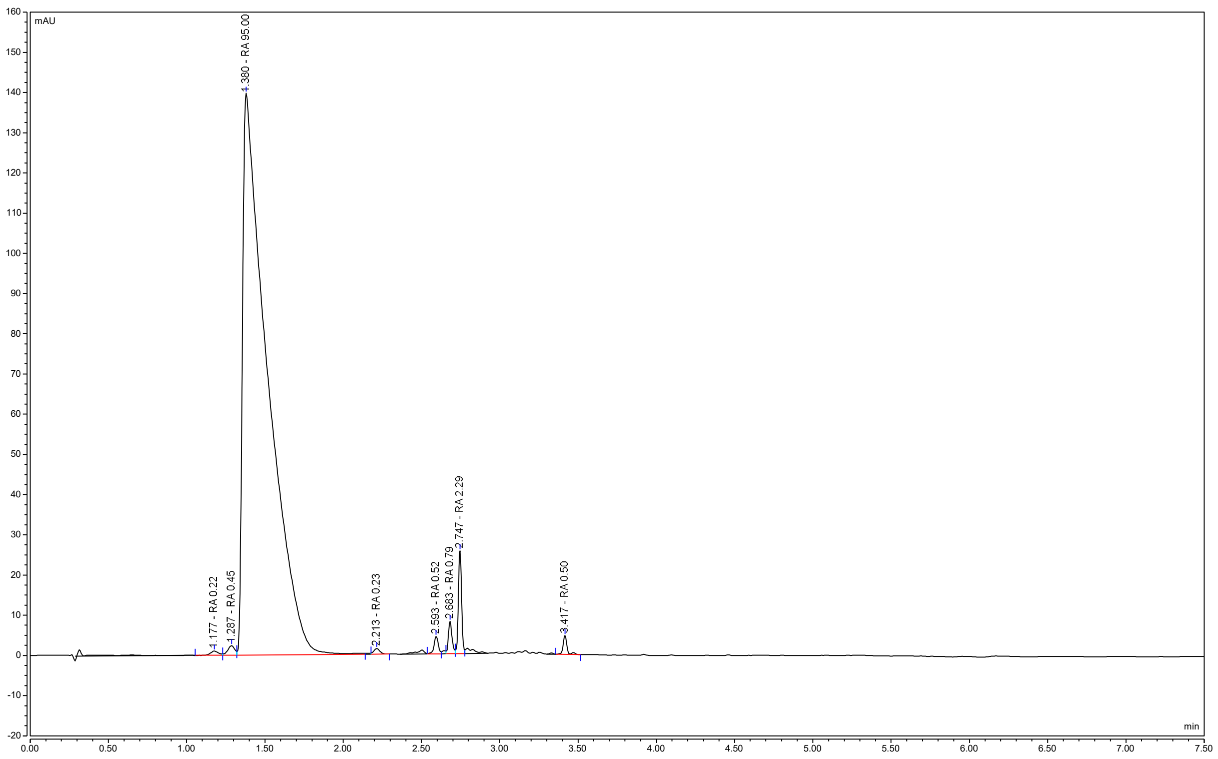

Figure S15 – UV-LC chromatogram for 5d.

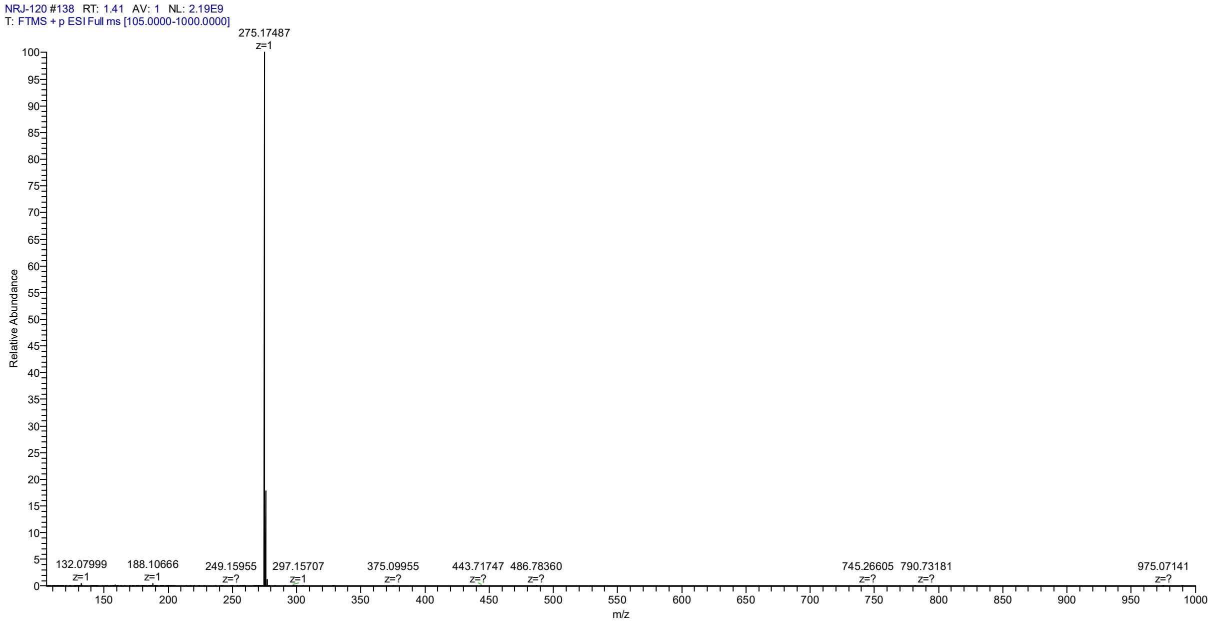

Figure S16 - HRMS spectrum for 5d.

1.5 1-(3-Thiomorpholinopropyl)-3,4-dihydroquinolin-2(1*H*)-one (5e)

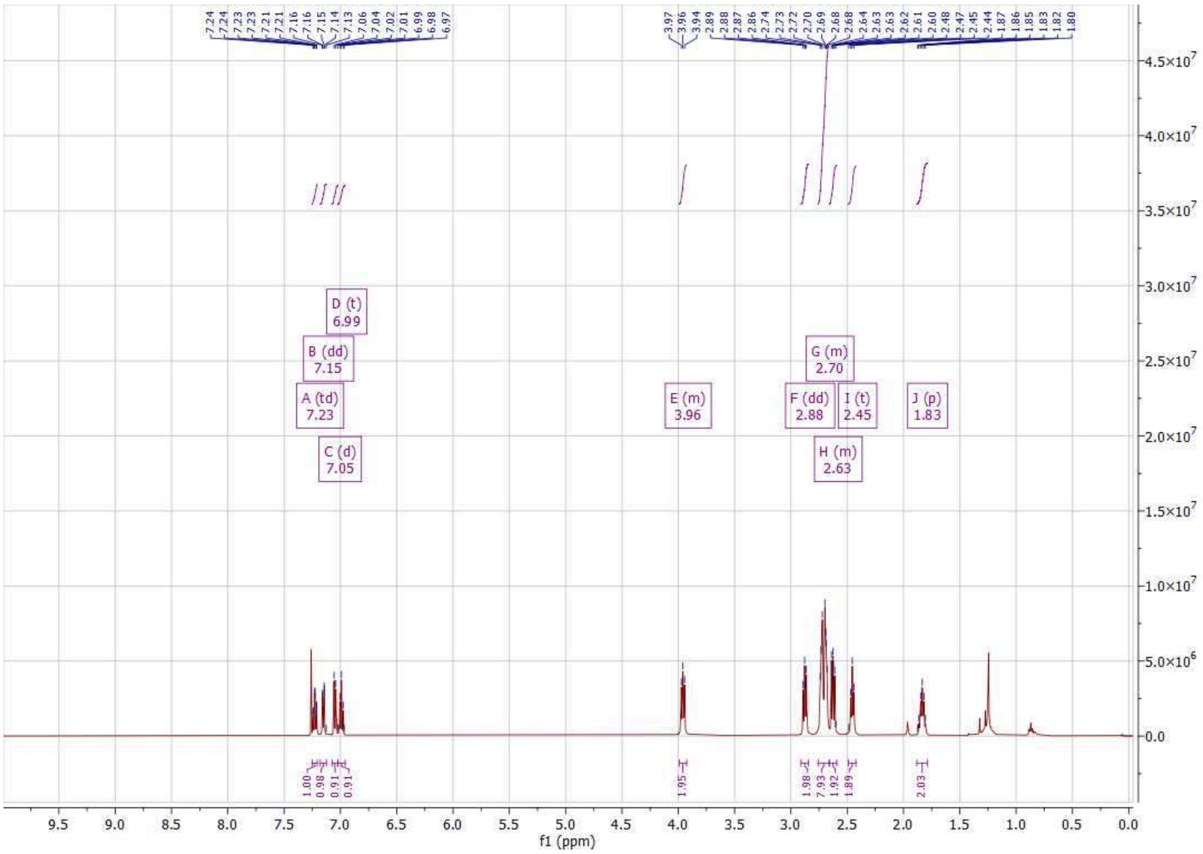

Figure S17 - ^{1}H NMR spectrum for 5e.

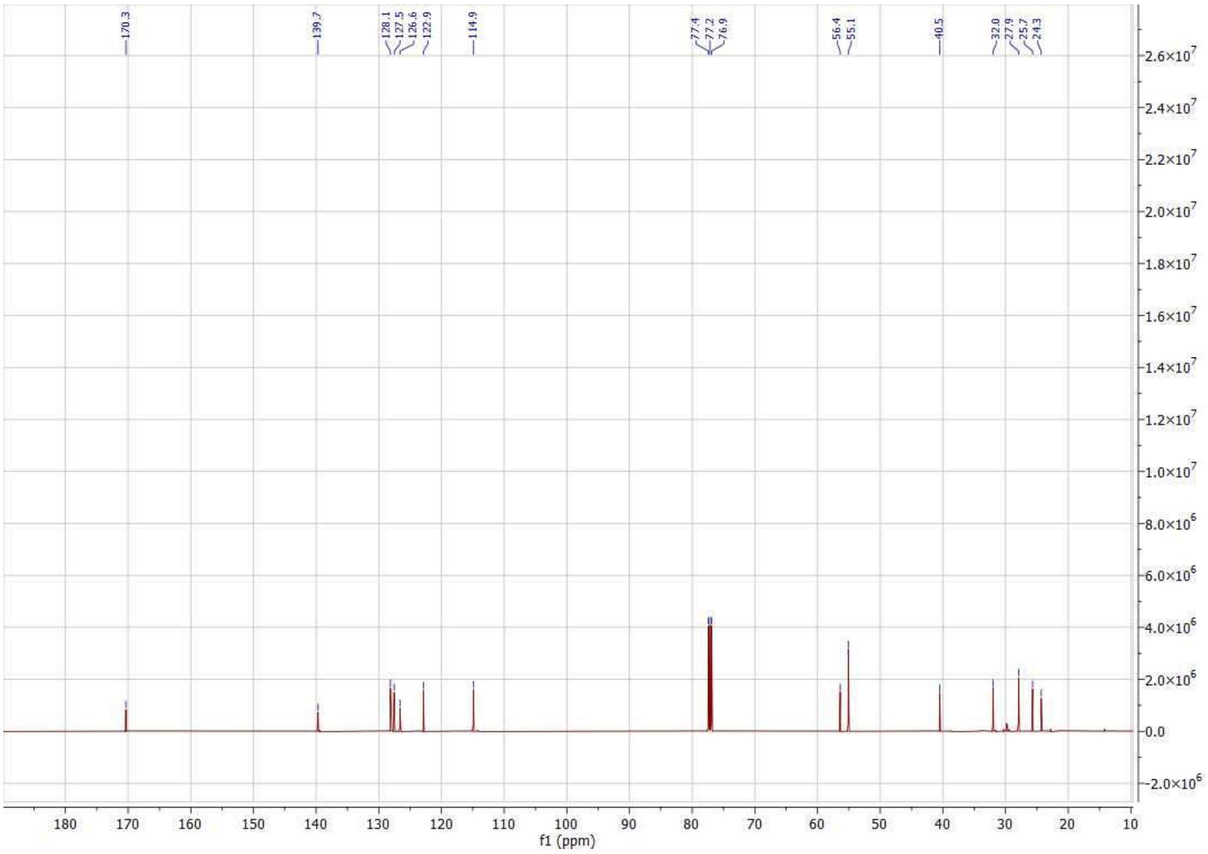

Figure S18 - ^{13}C NMR spectrum for 5e.

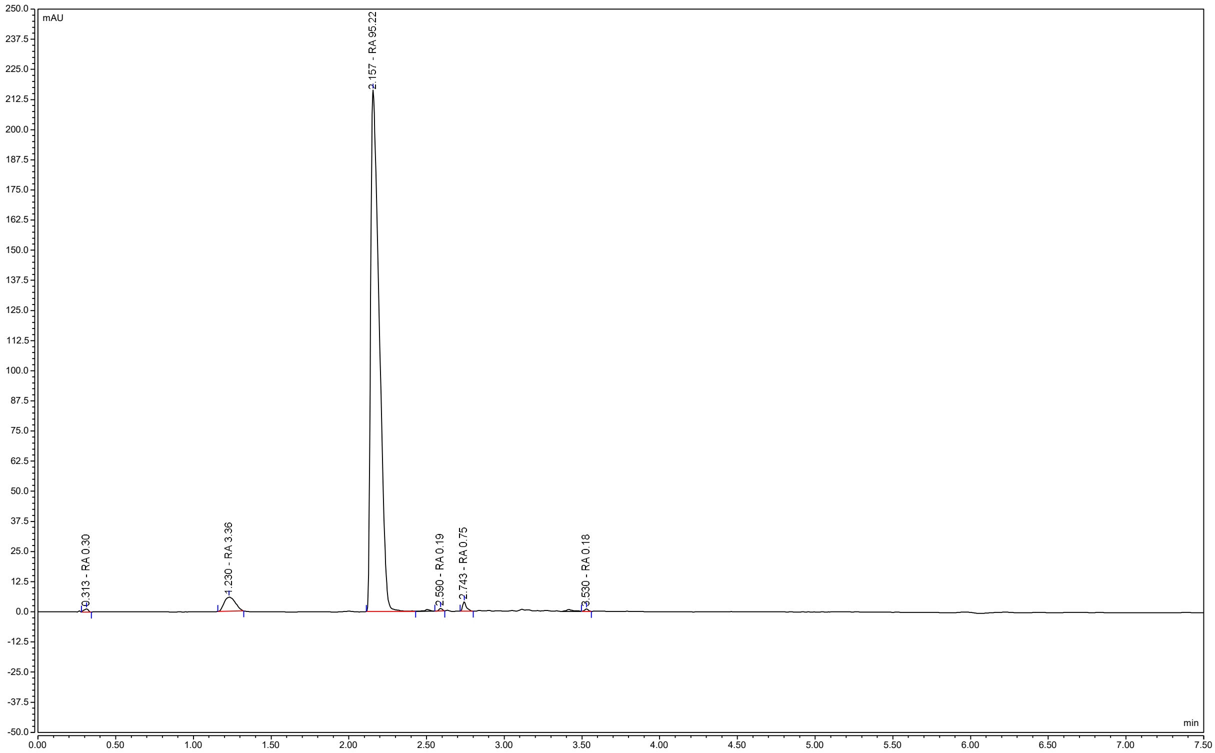

Figure S19 – UV-LC chromatogram for 5e.

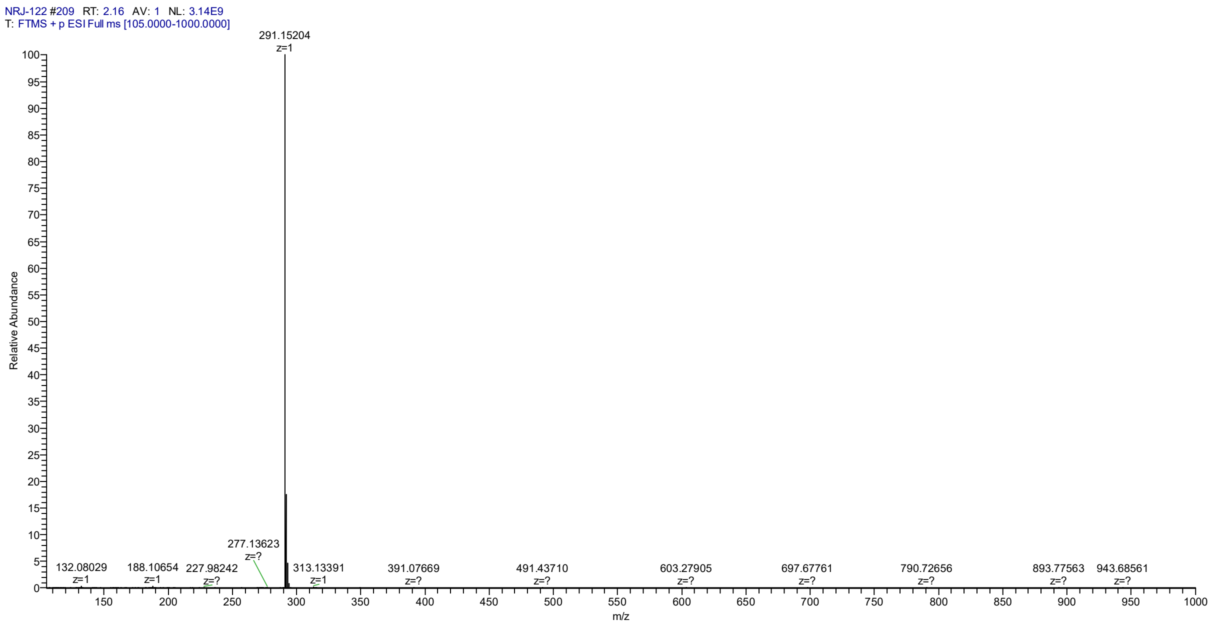

Figure S20 - HRMS spectrum for 5e.

1.6 1-(3-(Diethylamino)propyl)-3,4-dihydroquinolin-2(1*H*)-one (5f)

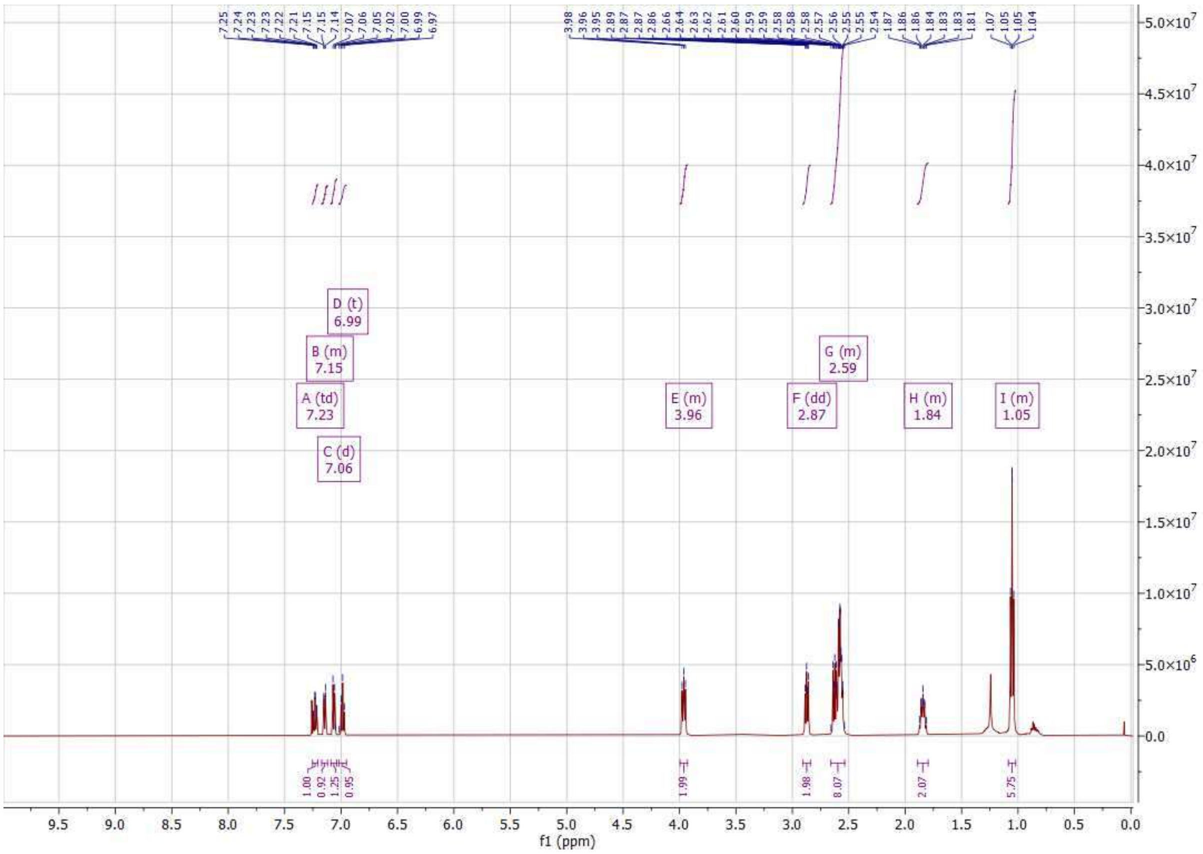

Figure S21 - ^{1}H NMR spectrum for 5f.

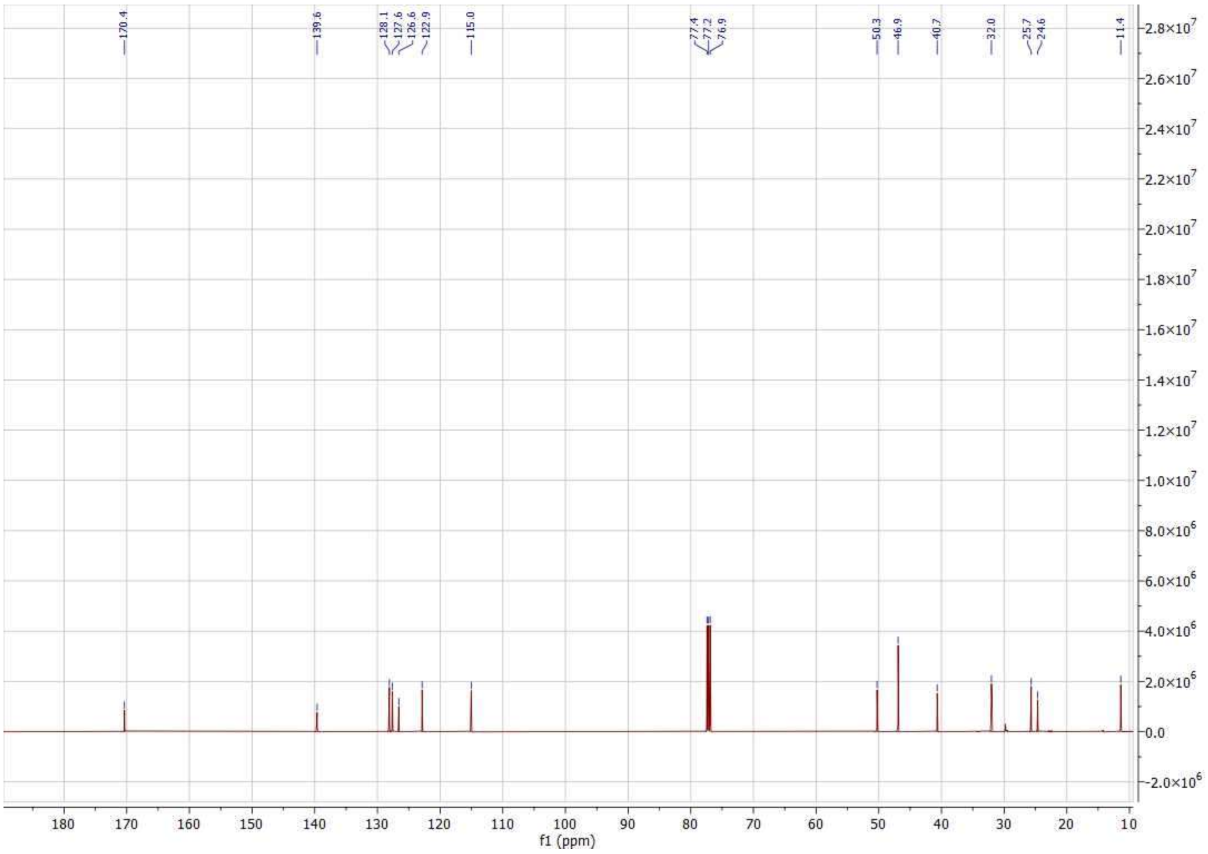

Figure S22 - ^{13}C NMR spectrum for 5f.

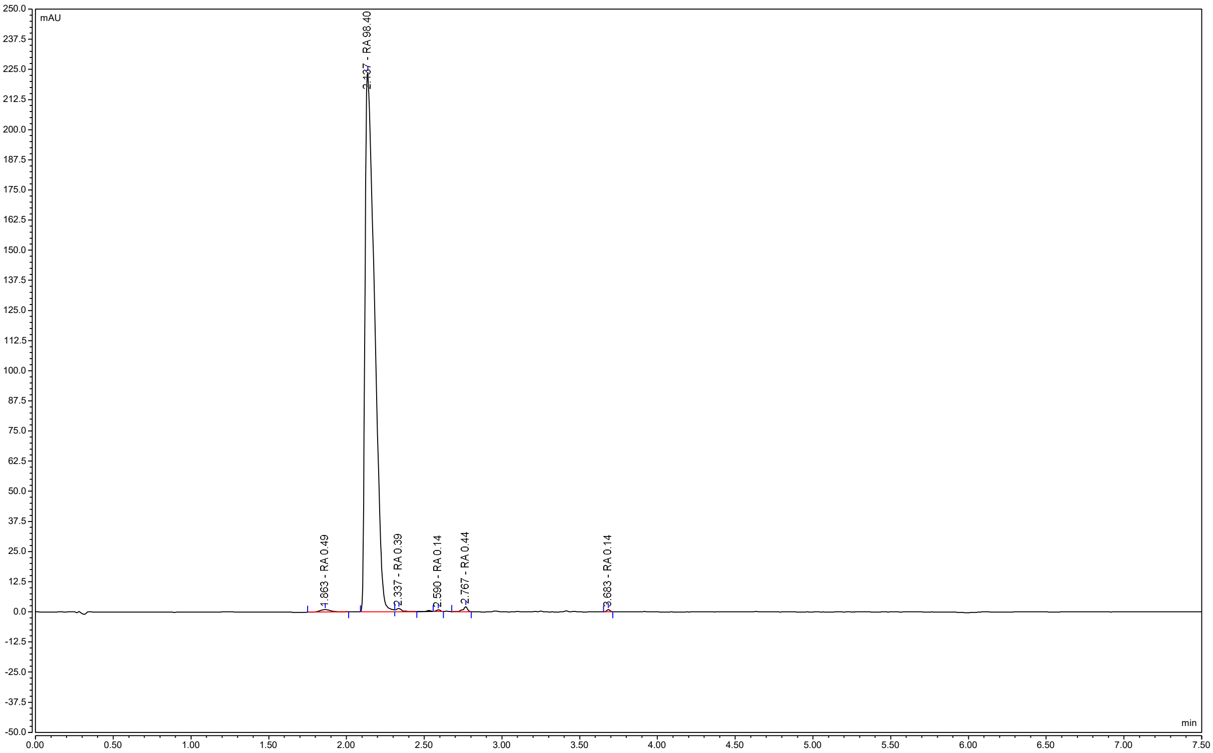

Figure S23 – UV-LC chromatogram for 5f.

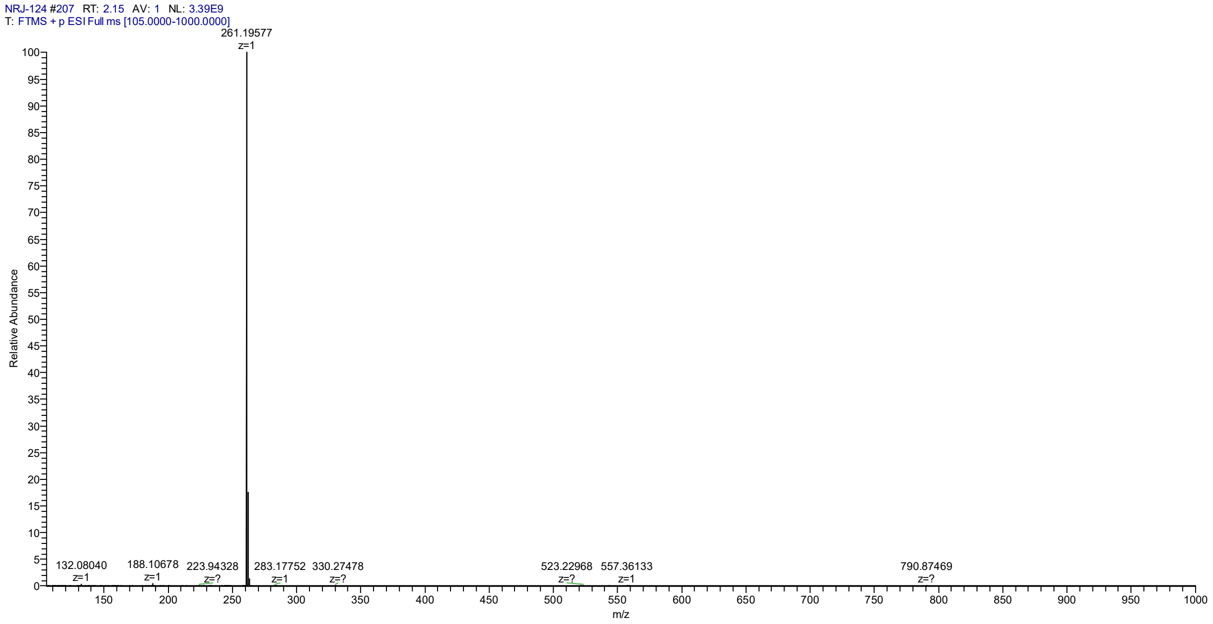

Figure S24 - HRMS spectrum for 5f.

1.7 1-(3-((2-Methoxyethyl)(methyl)amino)propyl)-3,4-dihydroquinolin-2(1*H*)-one (5g)

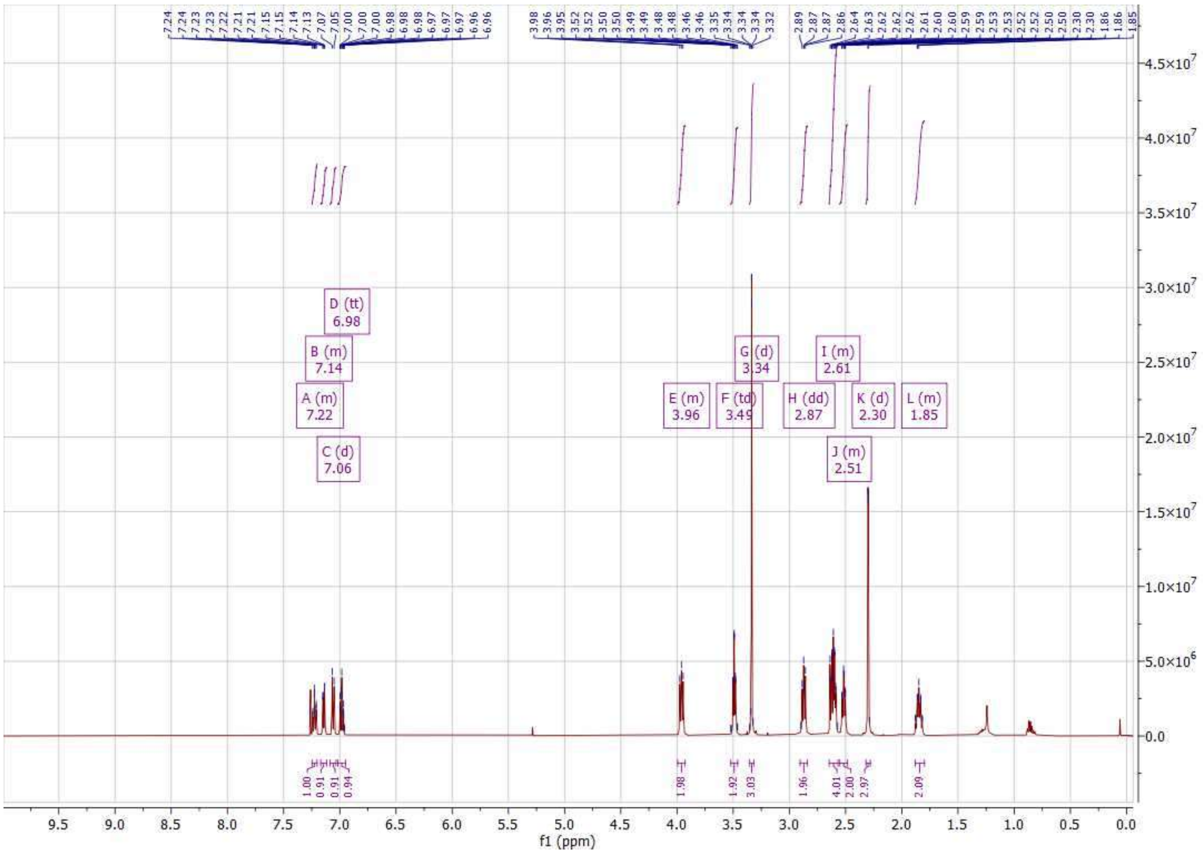

Figure S25 - ^{1}H NMR spectrum for 5g.

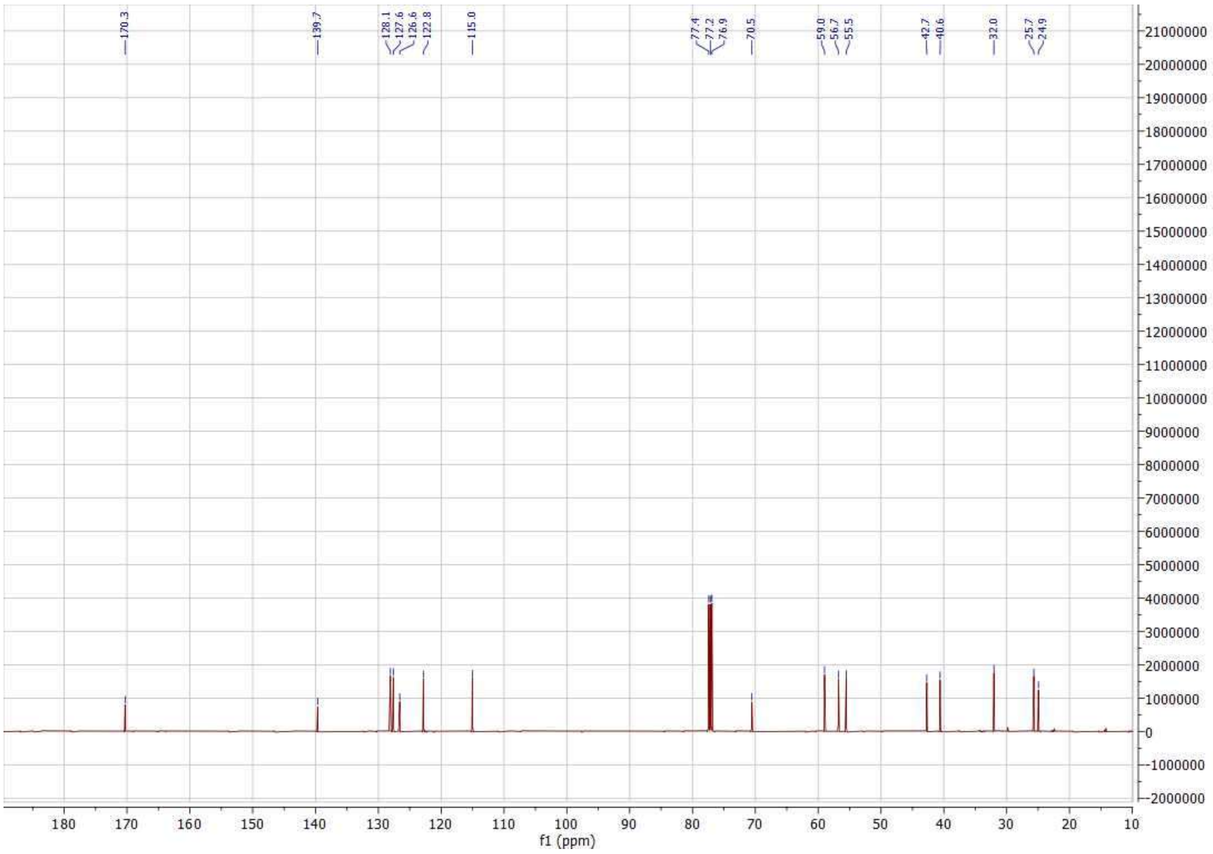

Figure S26 - ^{13}C NMR spectrum for 5g.

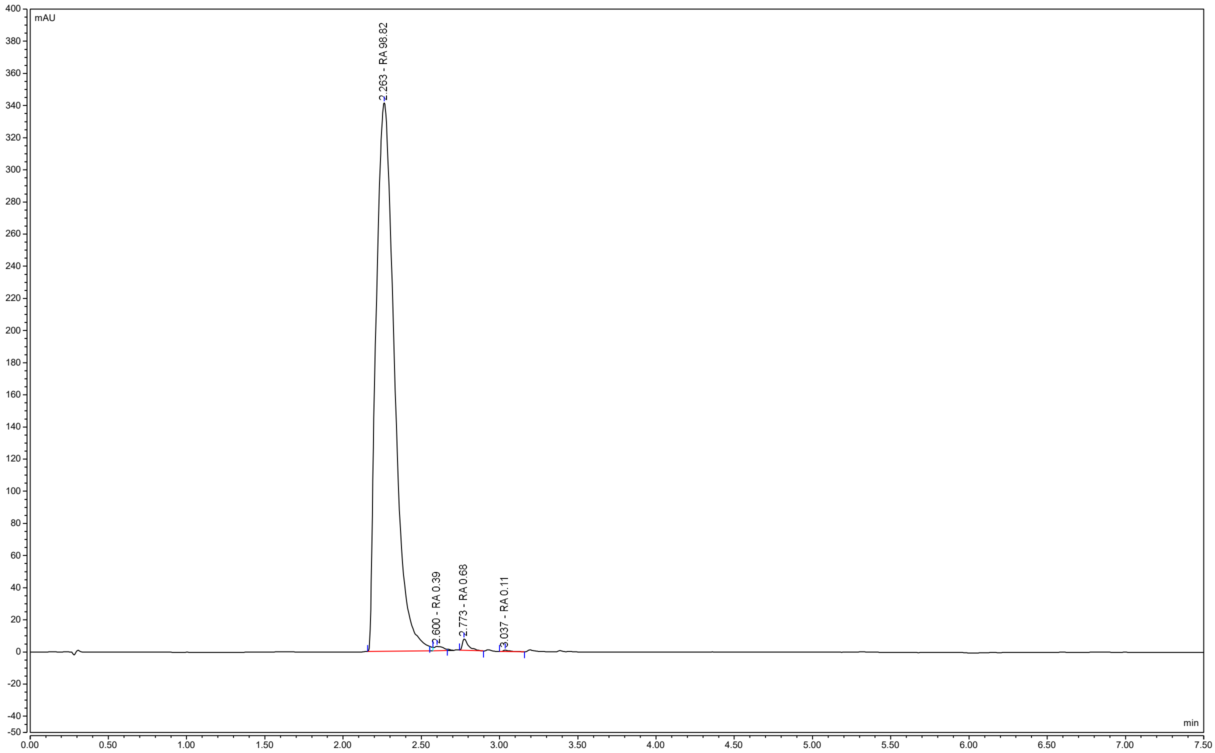

Figure S27 – UV-LC chromatogram for 5g.

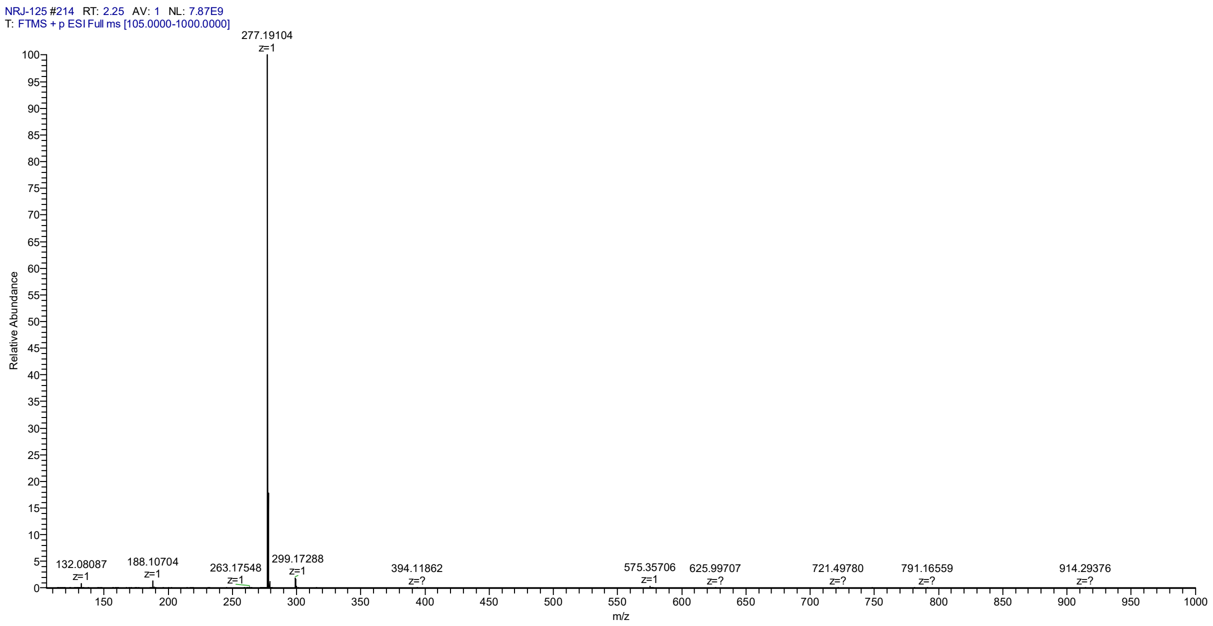

Figure S28 - HRMS spectrum for 5g.

1.8 1-(4-(Pyrrolidin-1-yl)butyl)-3,4-dihydroquinolin-2(1*H*)-one (6a)

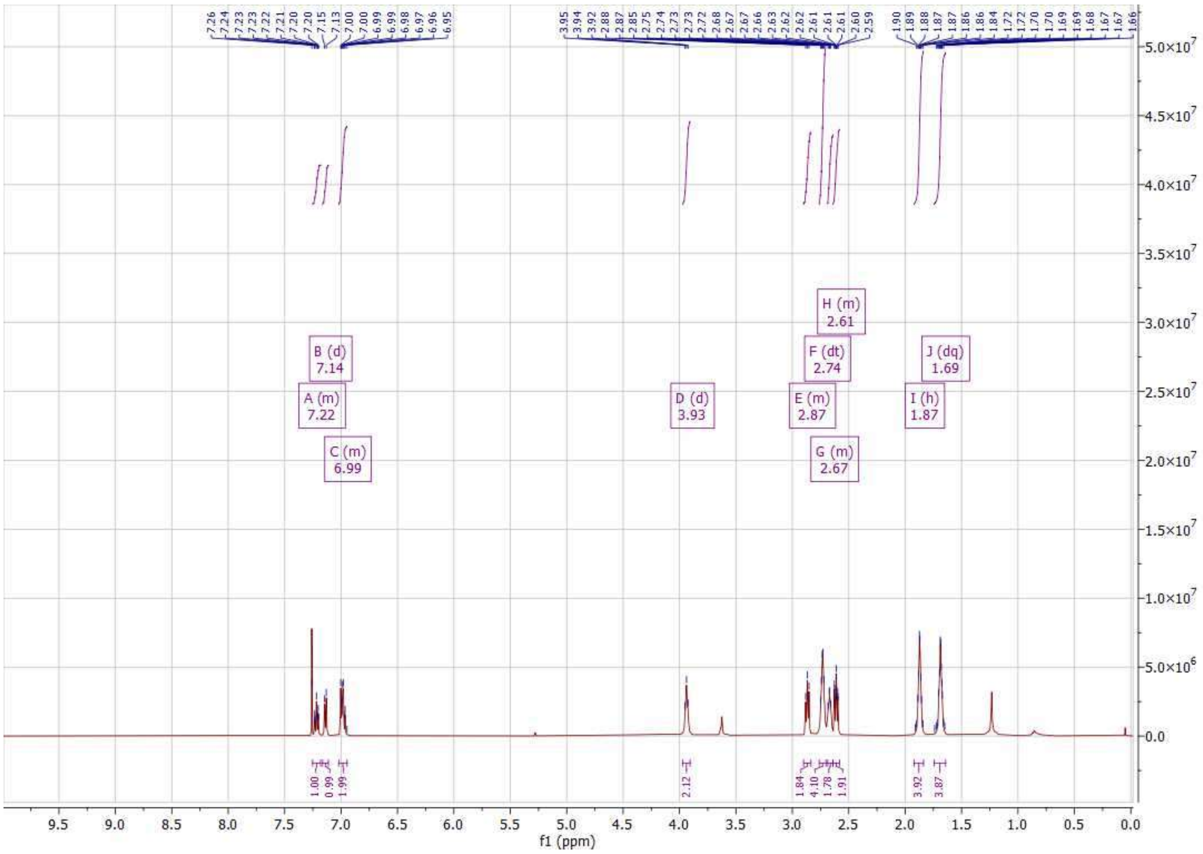

Figure S29 - ^{1}H NMR spectrum for 6a.

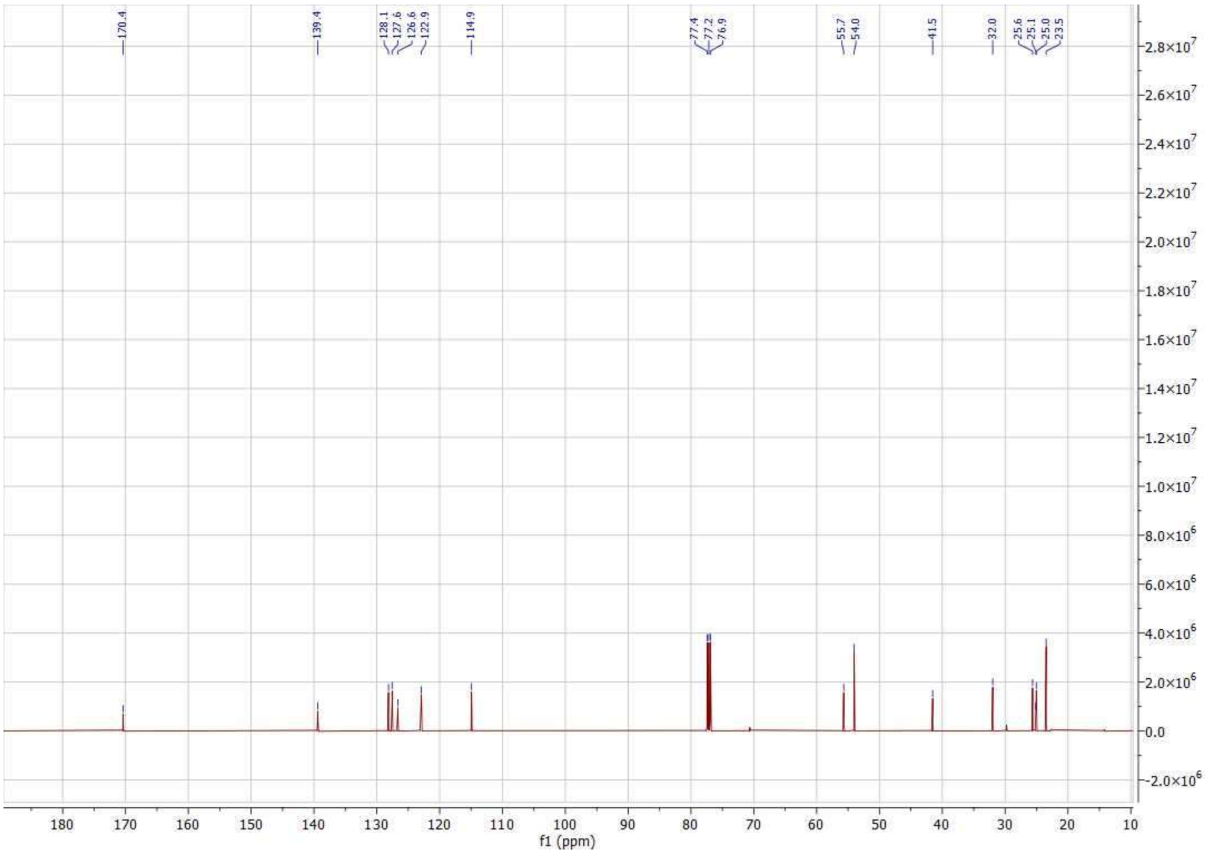

Figure S30 - ^{13}C NMR spectrum for 6a.

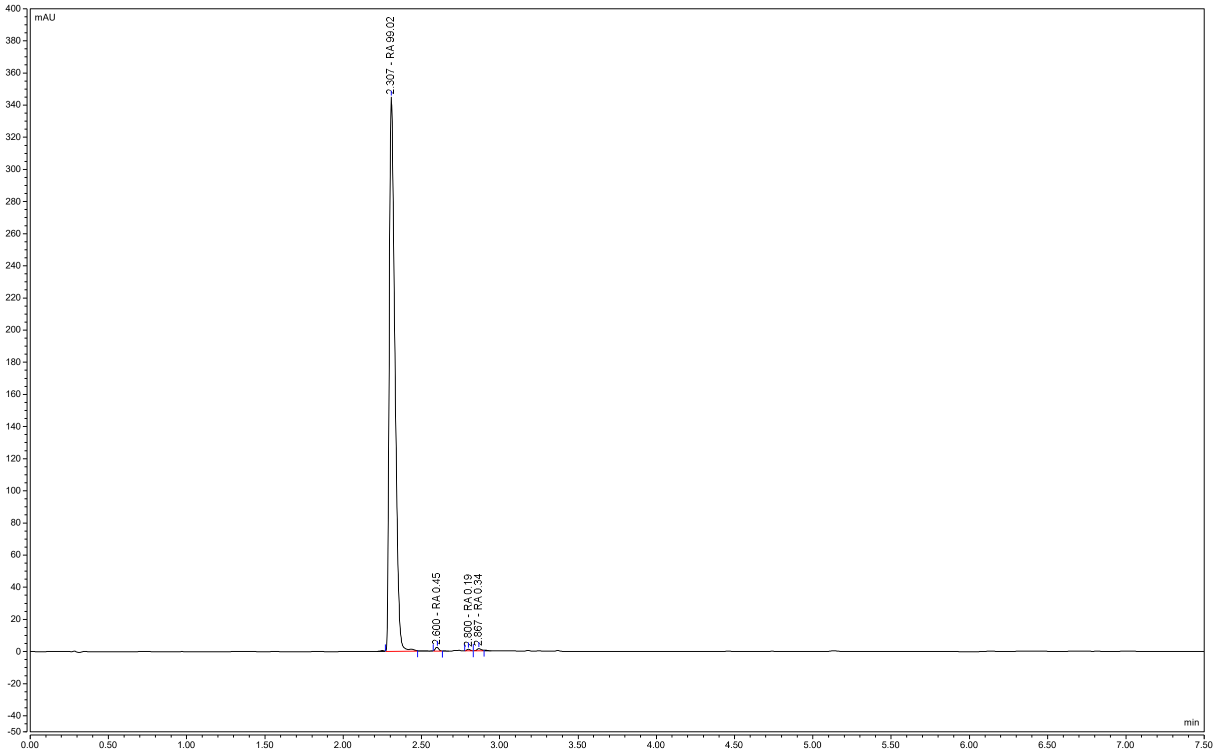

Figure S31 – UV-LC chromatogram for 6a.

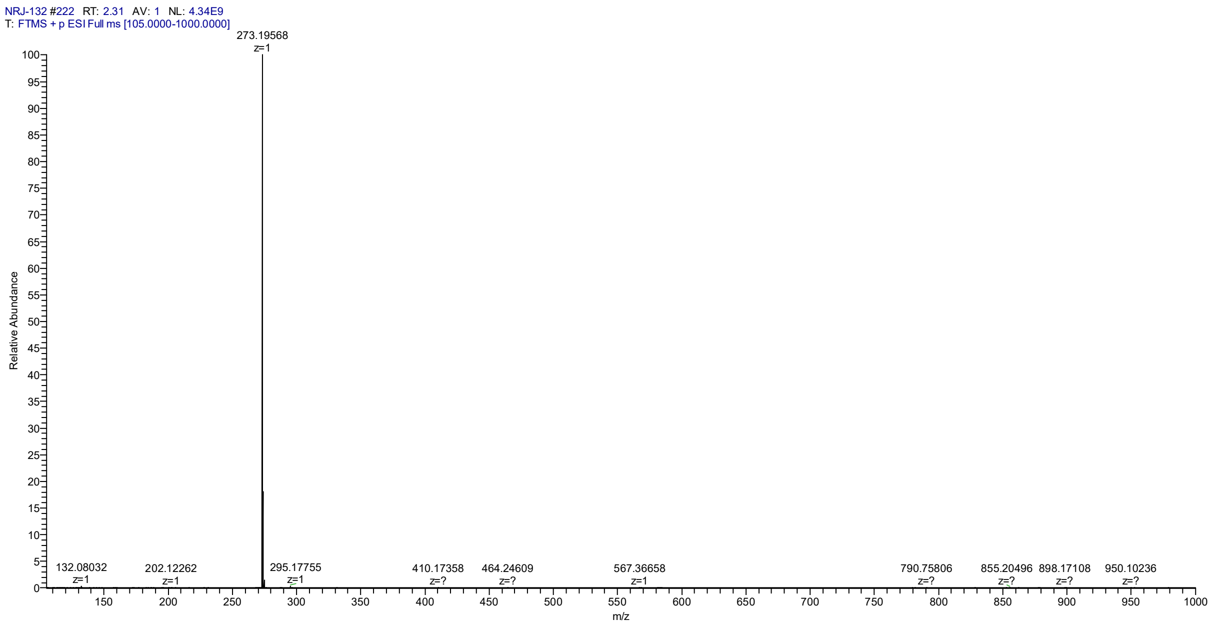

Figure S32 - HRMS spectrum for 6a.

1.9 1-(4-(Piperidin-1-yl)butyl)-3,4-dihydroquinolin-2(1H)-one (6b)

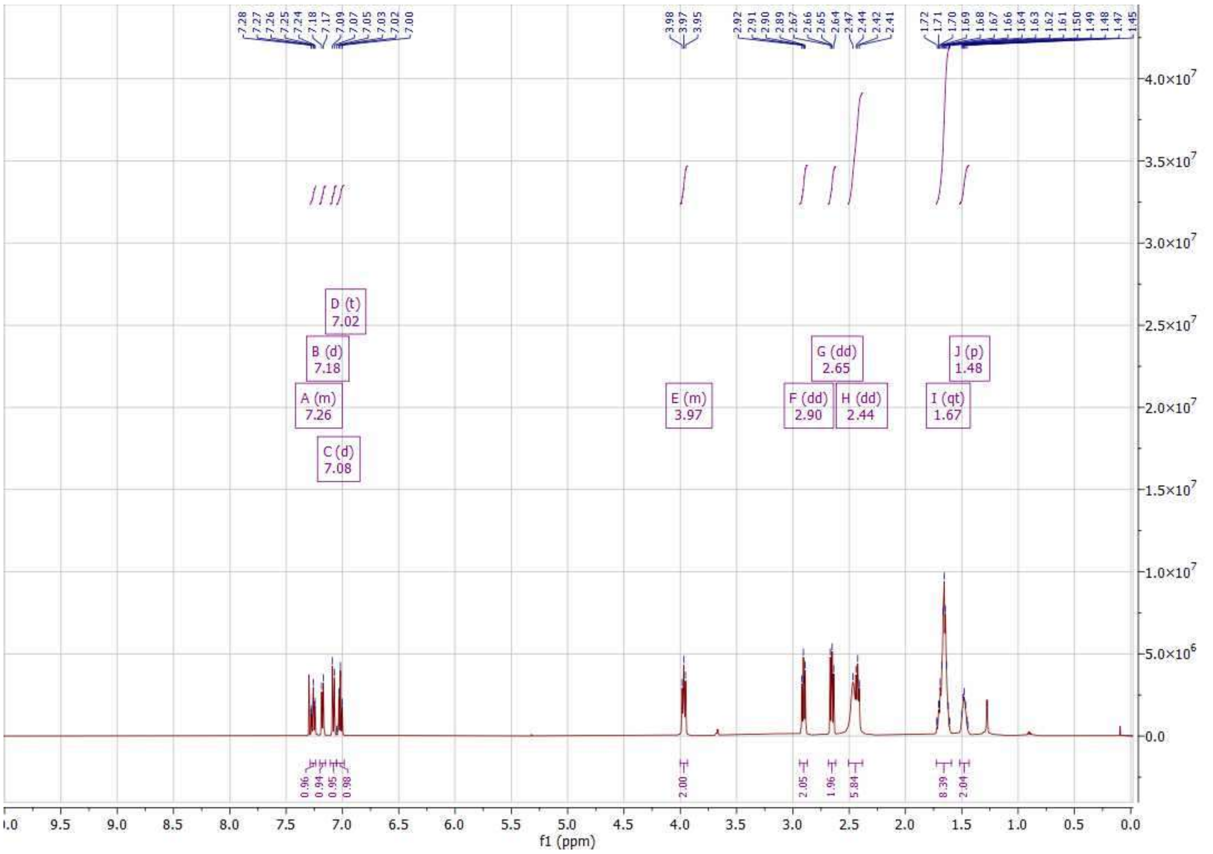

Figure S33 - ^{1}H NMR spectrum for 6b.

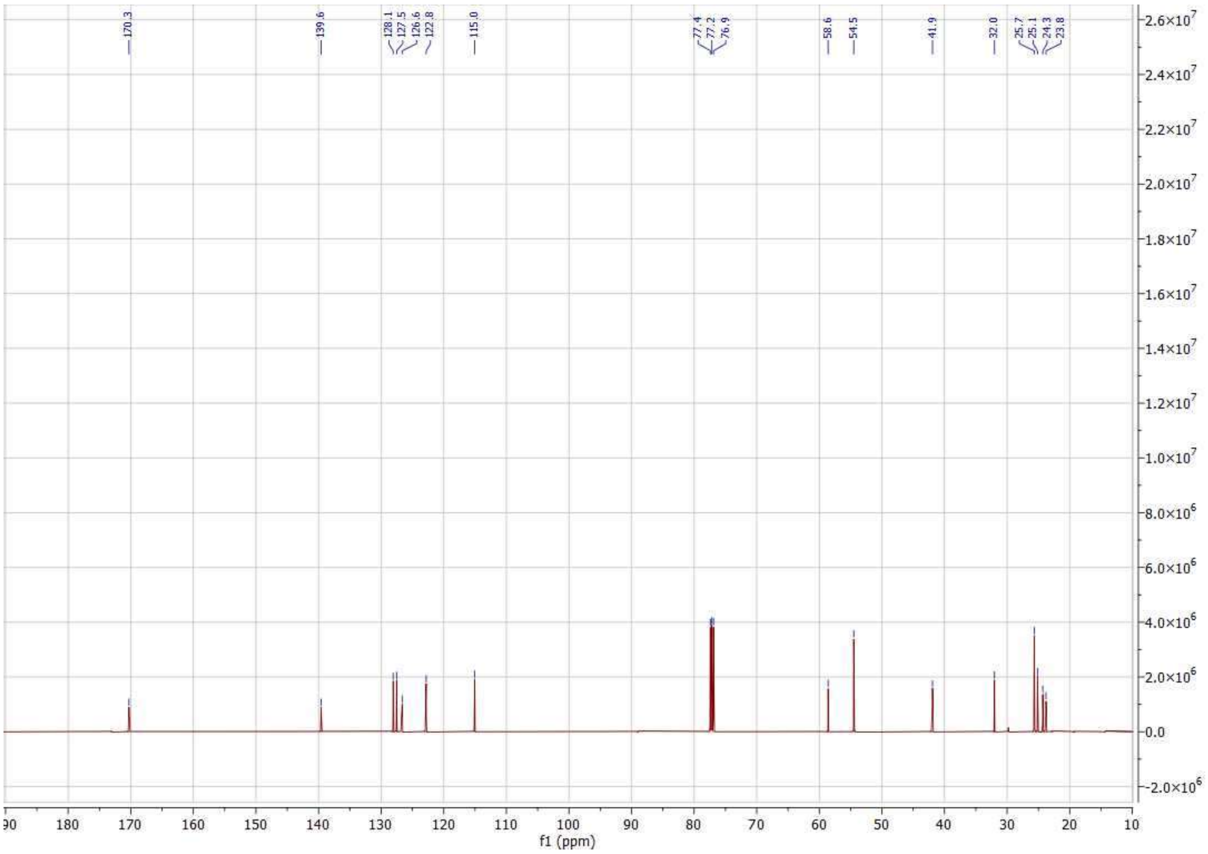

Figure S34 - ^{13}C NMR spectrum for 6b.

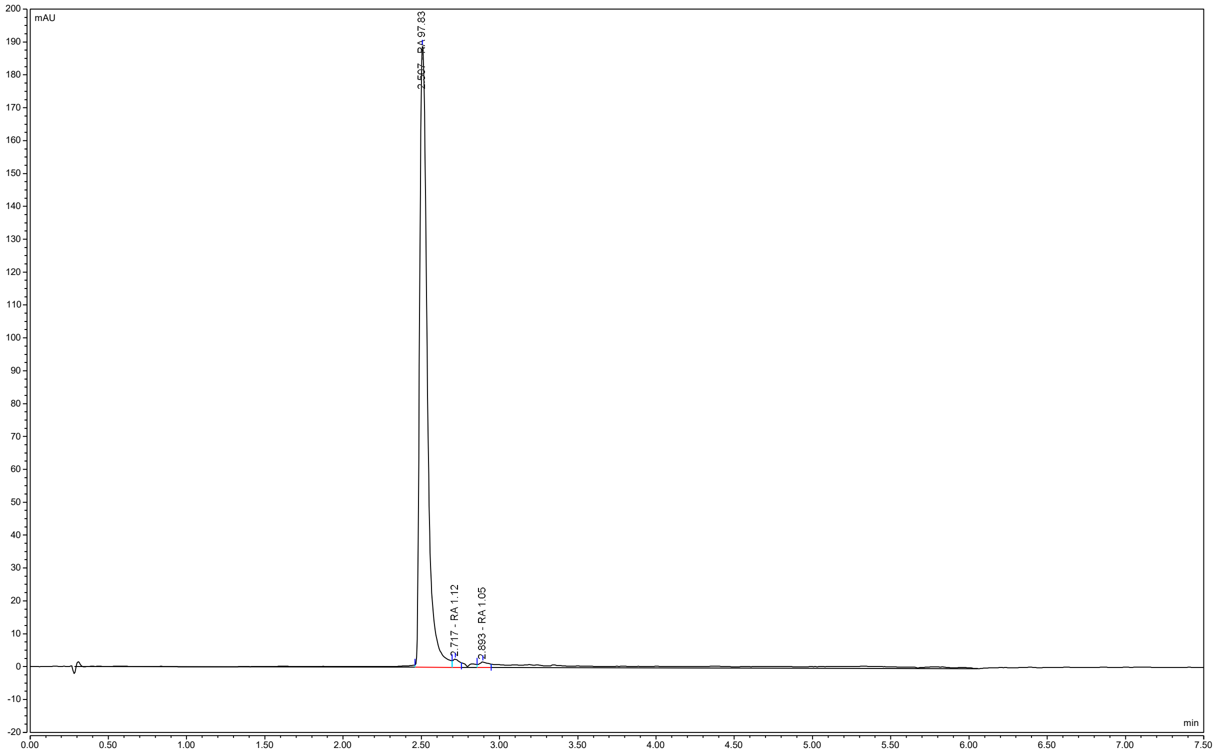

Figure S35 – UV-LC chromatogram for 6b.

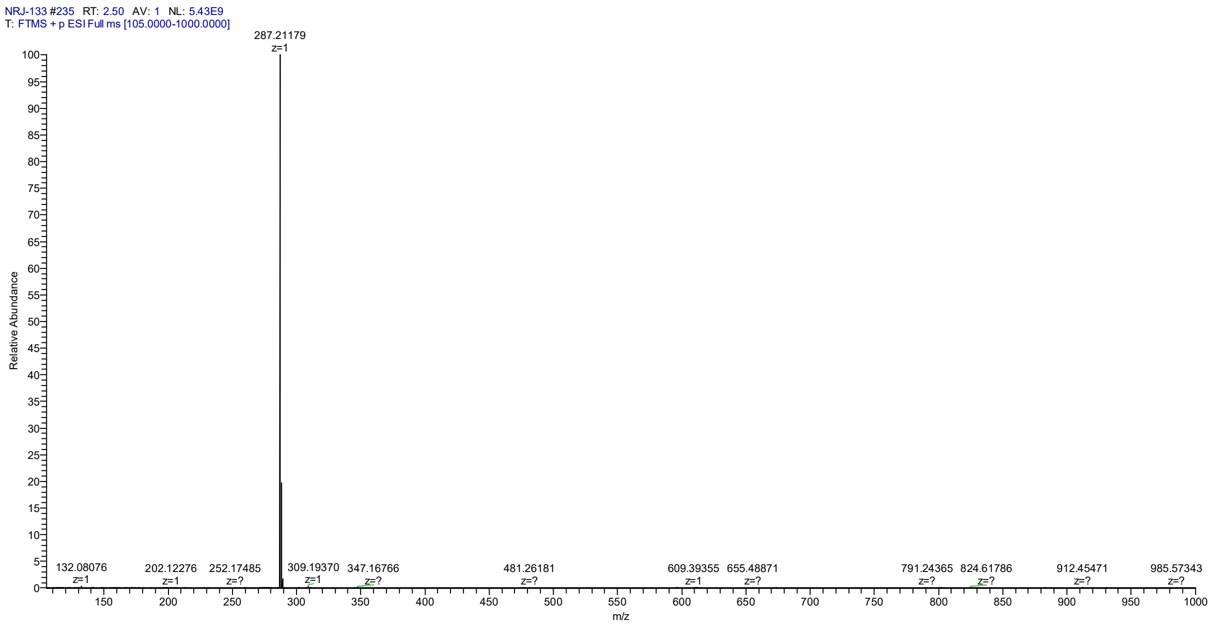

Figure S36 - HRMS spectrum for 6b.

1.10 1-(4-(4-Methylpiperazin-1-yl)butyl)-3,4-dihydroquinolin-2(1H)-one (6c)

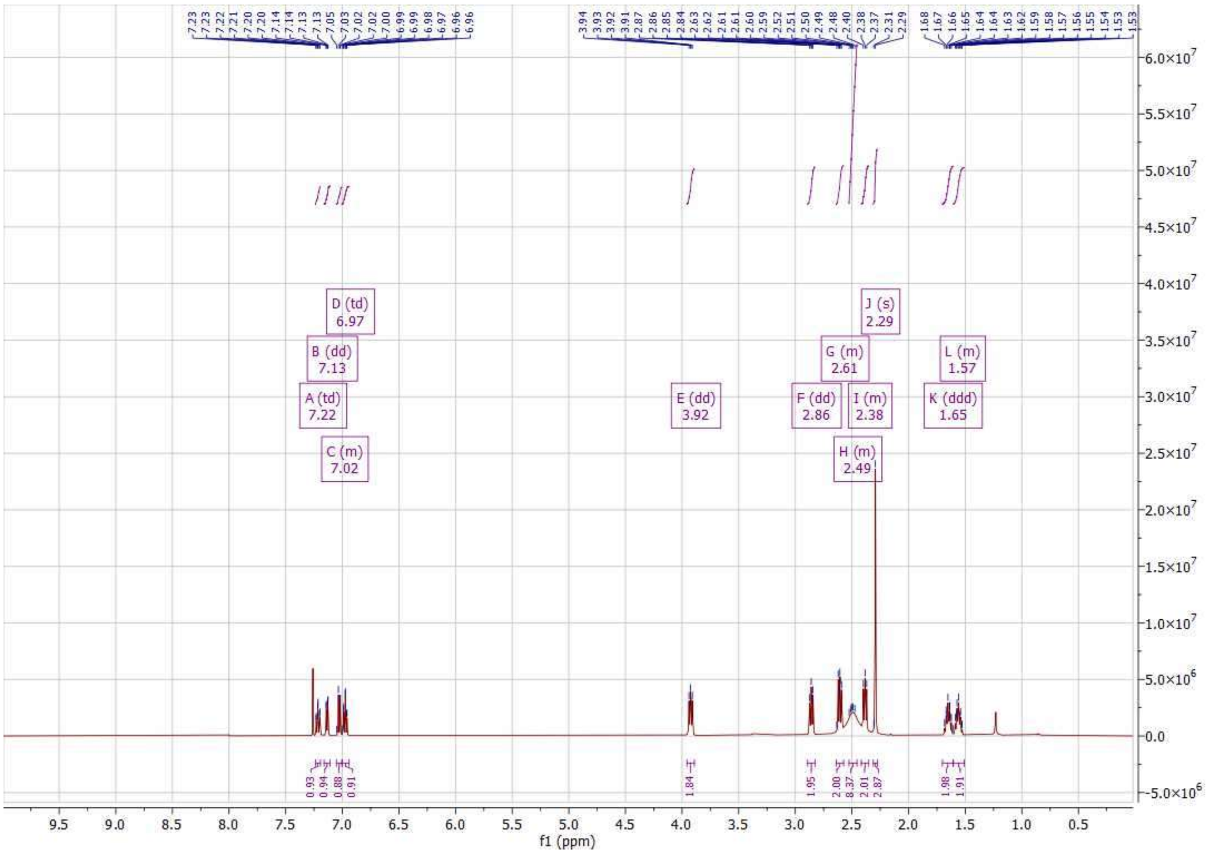

Figure S37 - ^{1}H NMR spectrum for 6c.

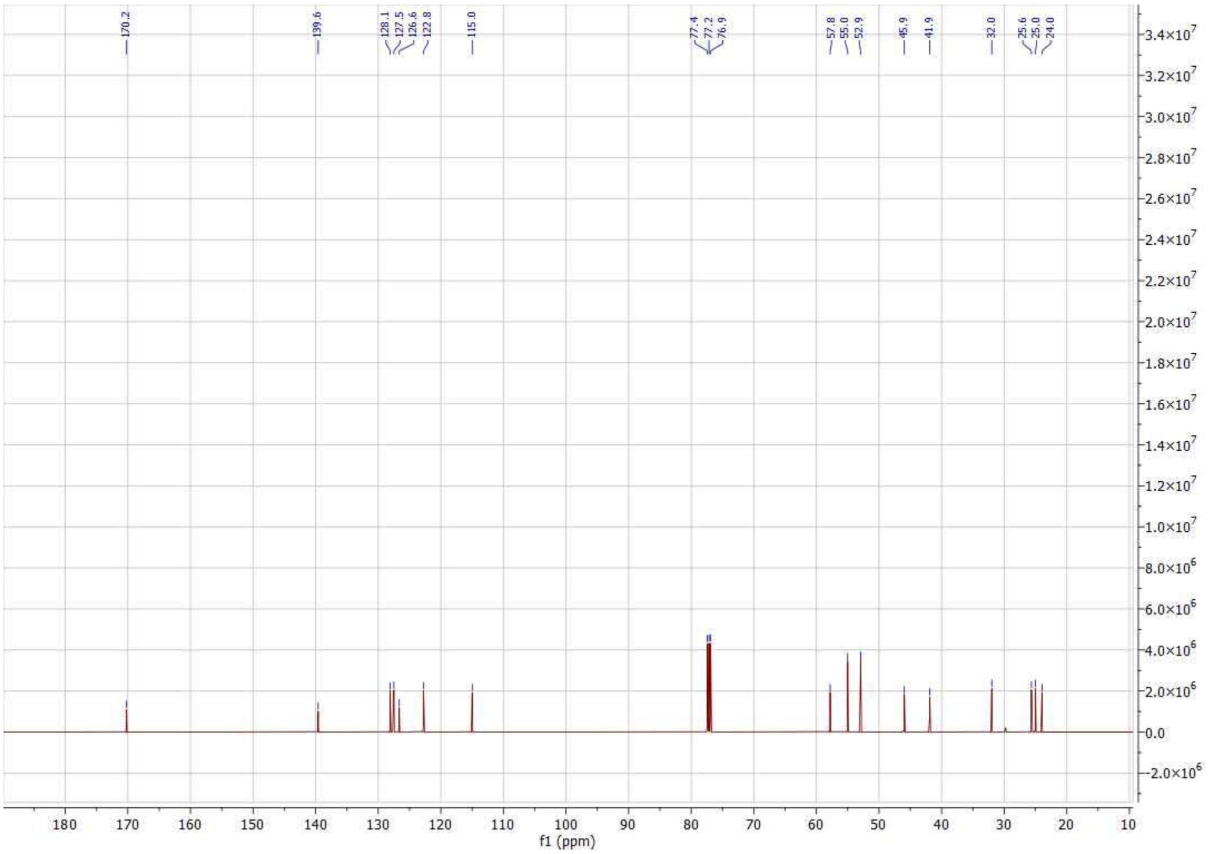

Figure S38 - ^{13}C NMR spectrum for 6c.

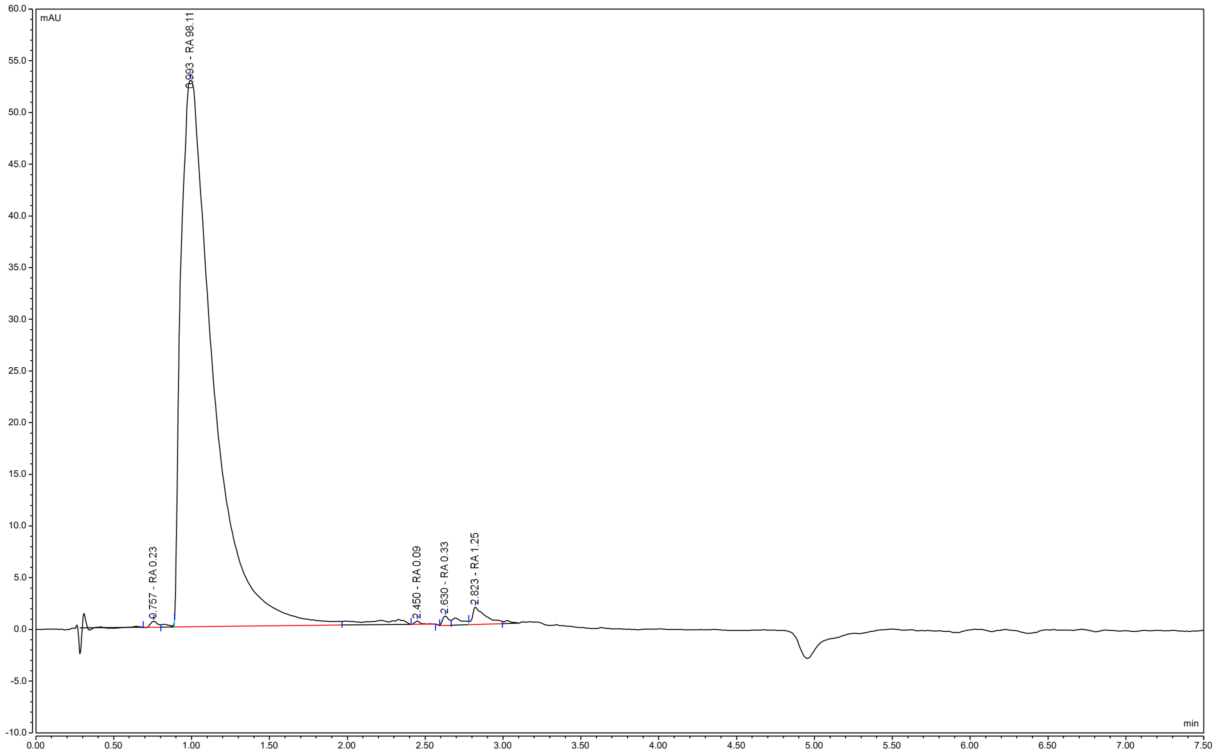

Figure S39 – UV-LC chromatogram for 6c.

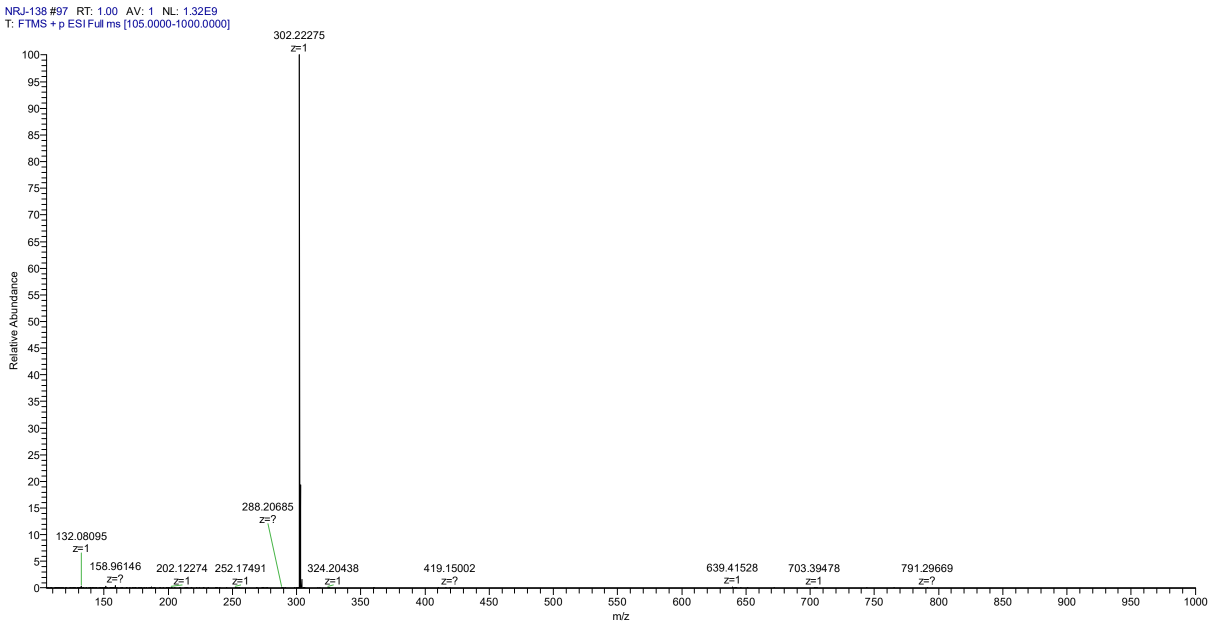

Figure S40 - HRMS spectrum for 6c.

1.11 1-(4-Morpholinobutyl)-3,4-dihydroquinolin-2(1*H*)-one (6d)

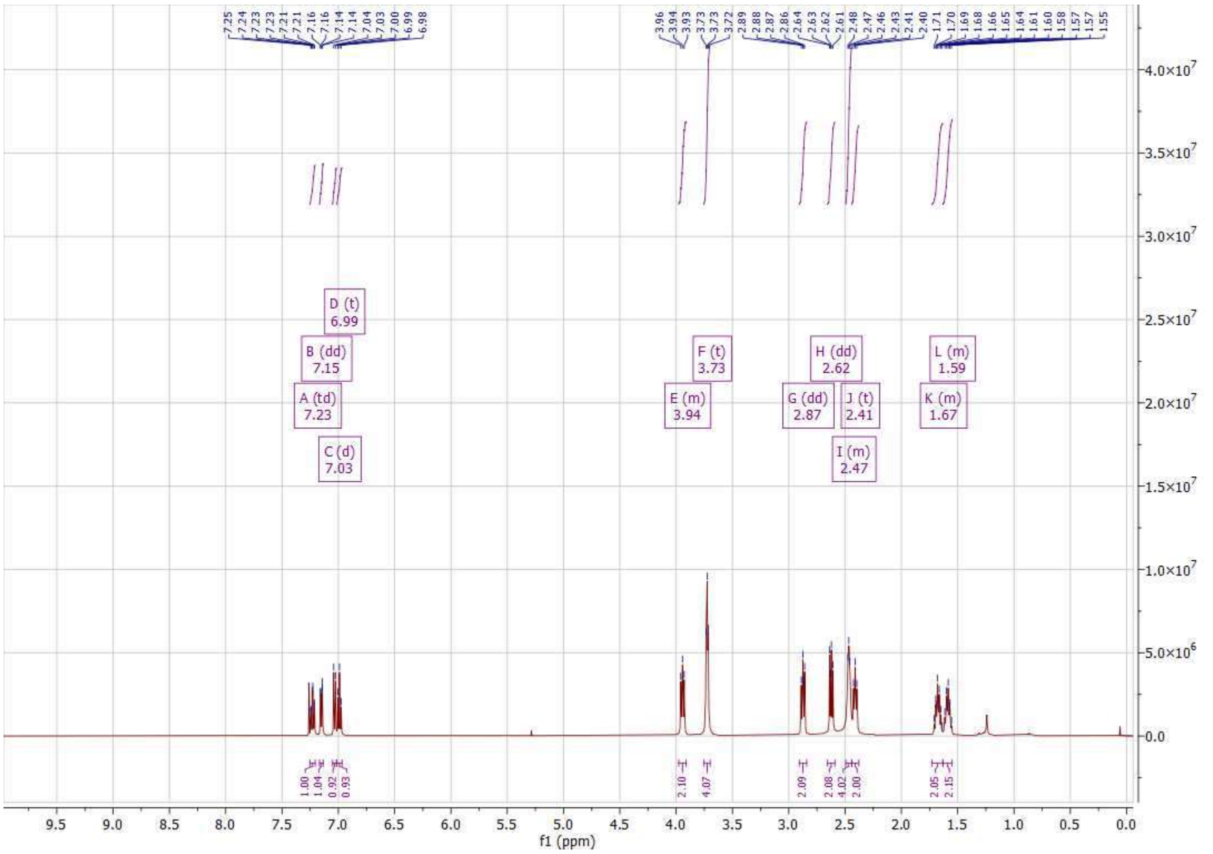

Figure S41 - ^{1}H NMR spectrum for 6d.

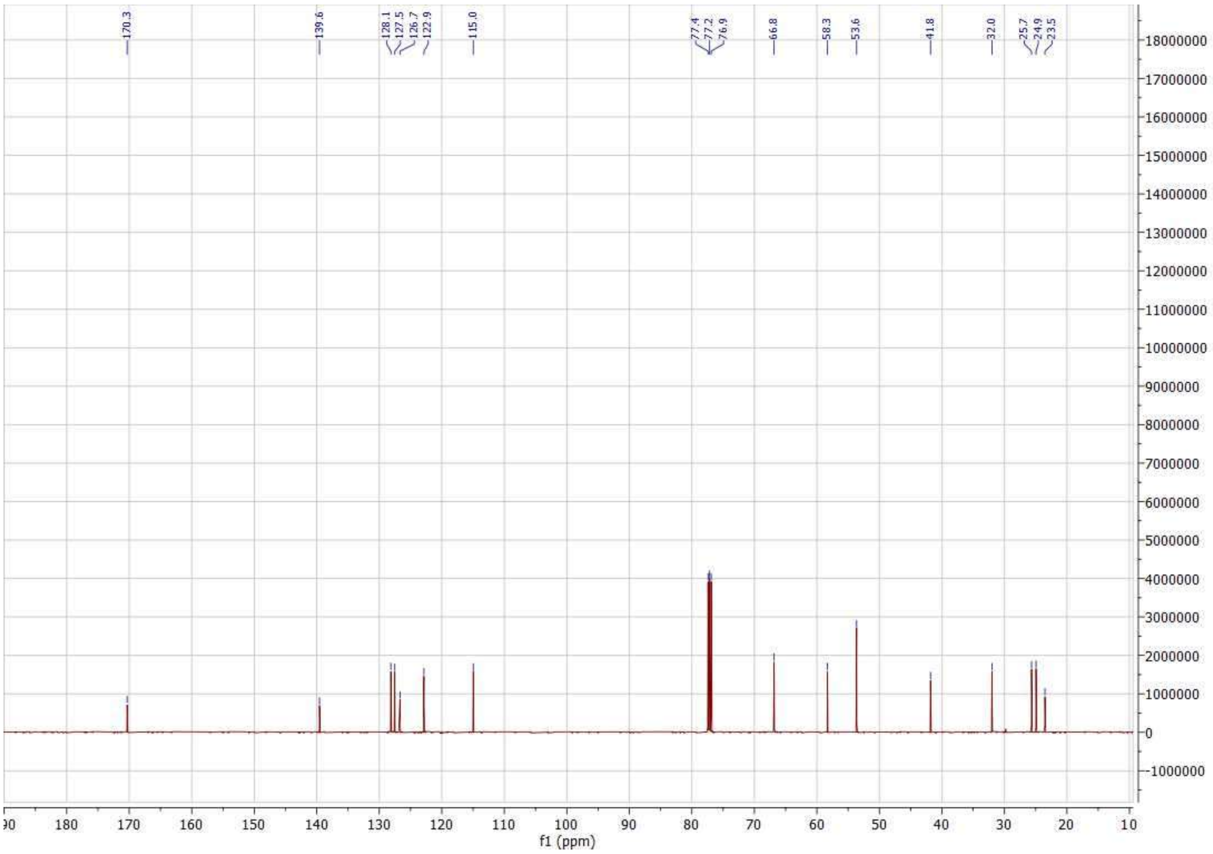

Figure S42 - ^{13}C NMR spectrum for 6d.

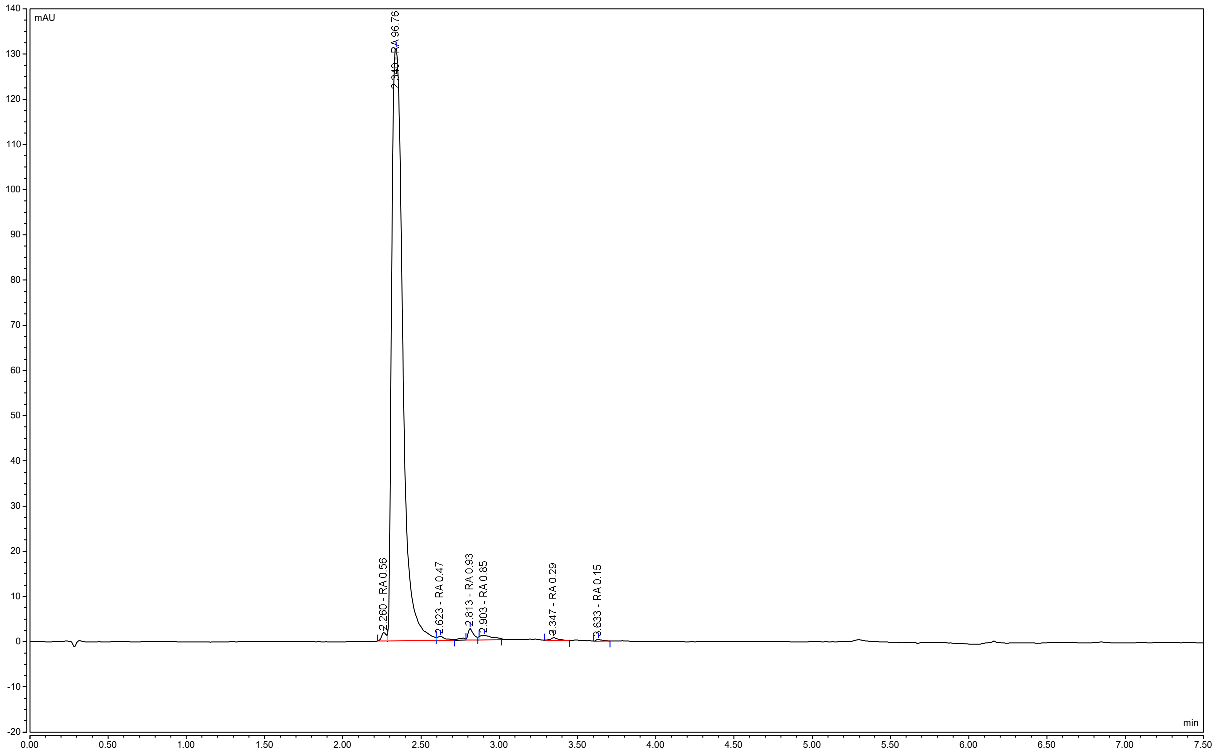

Figure S43 – UV-LC chromatogram for 6d.

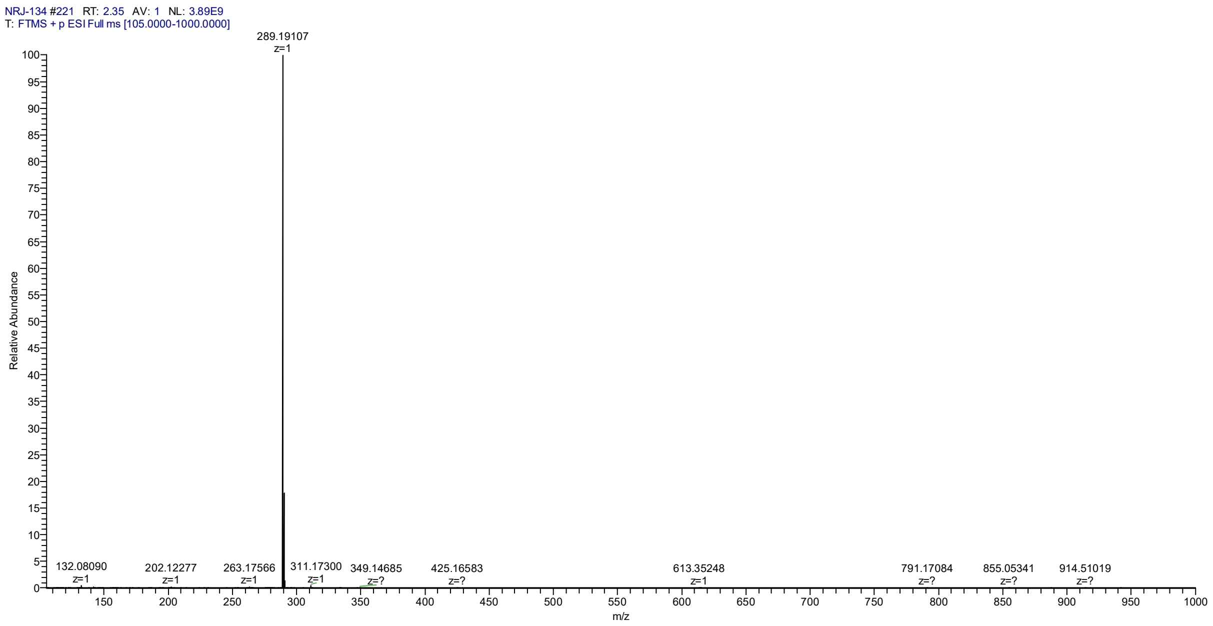

Figure S44 - HRMS spectrum for 6d.

1.12 1-(4-Thiomorpholinobutyl)-3,4-dihydroquinolin-2(1*H*)-one (6e)

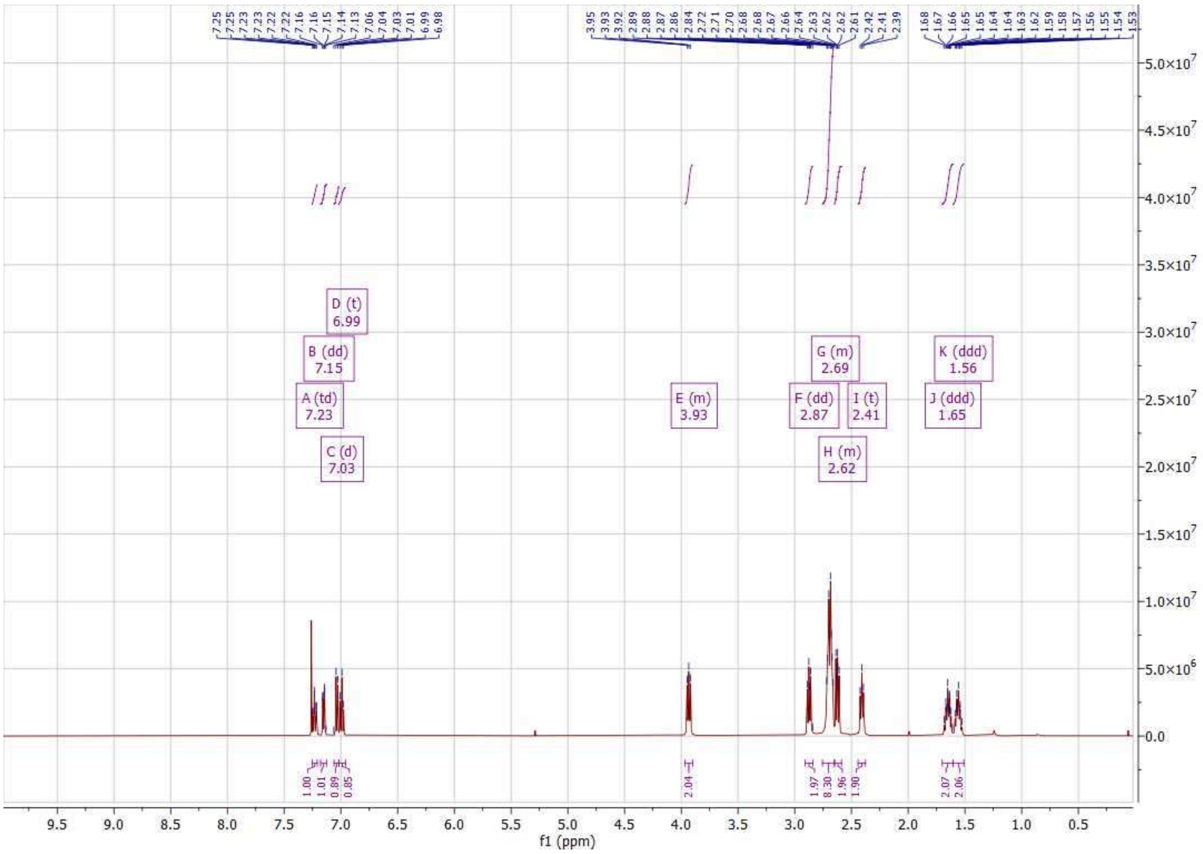

Figure S45 - ^{1}H NMR spectrum for 6e.

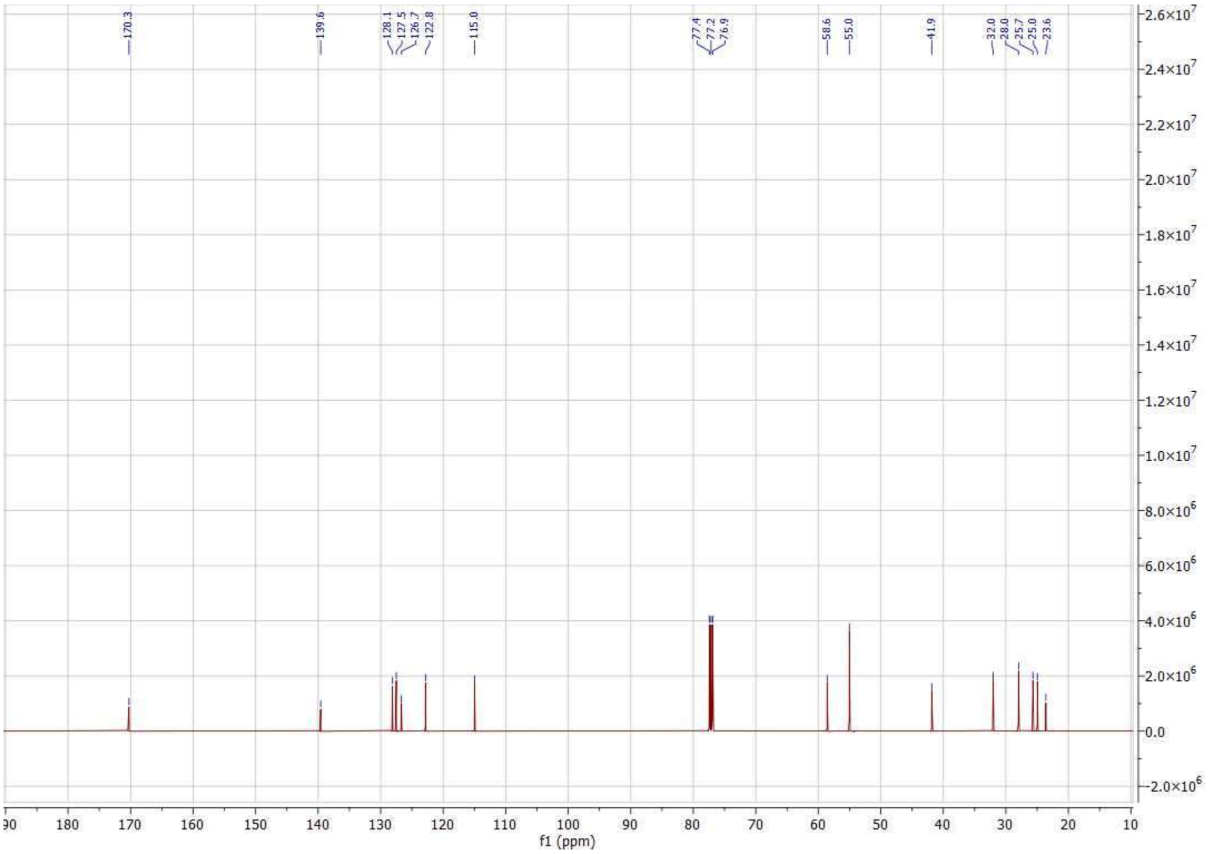

Figure S46 - ^{13}C NMR spectrum for 6e.

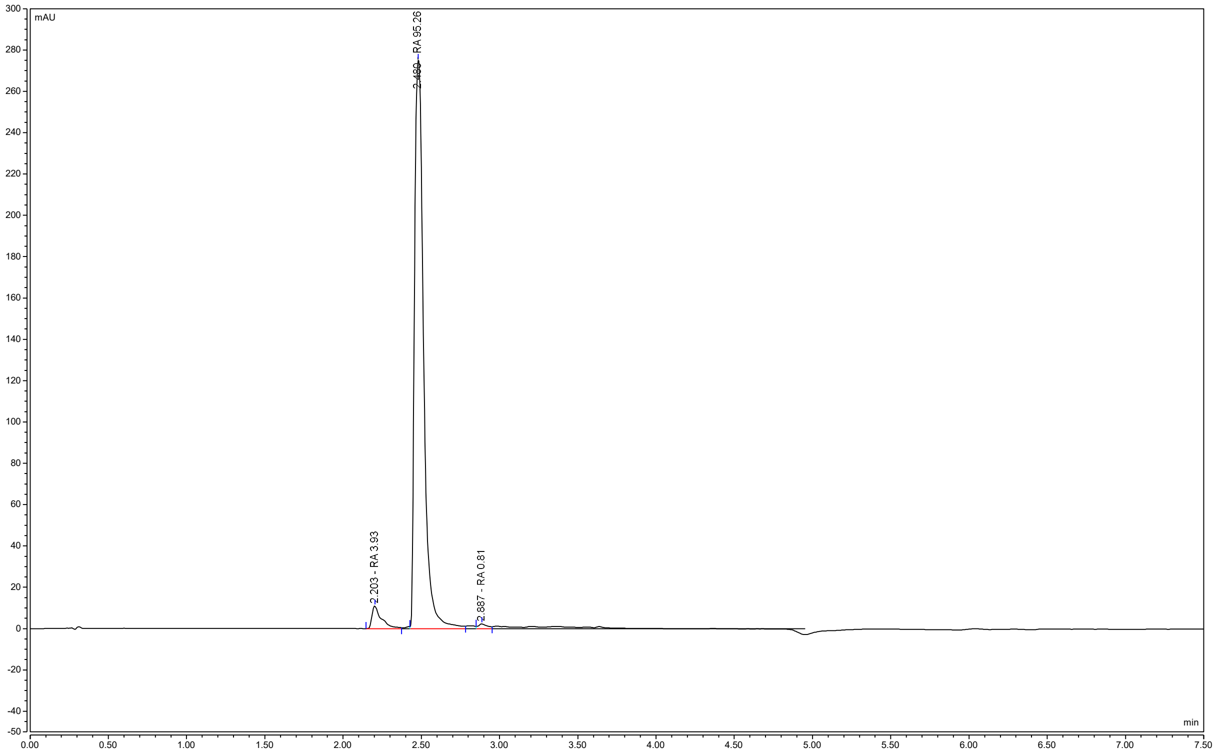

Figure S47 – UV-LC chromatogram for 6e.

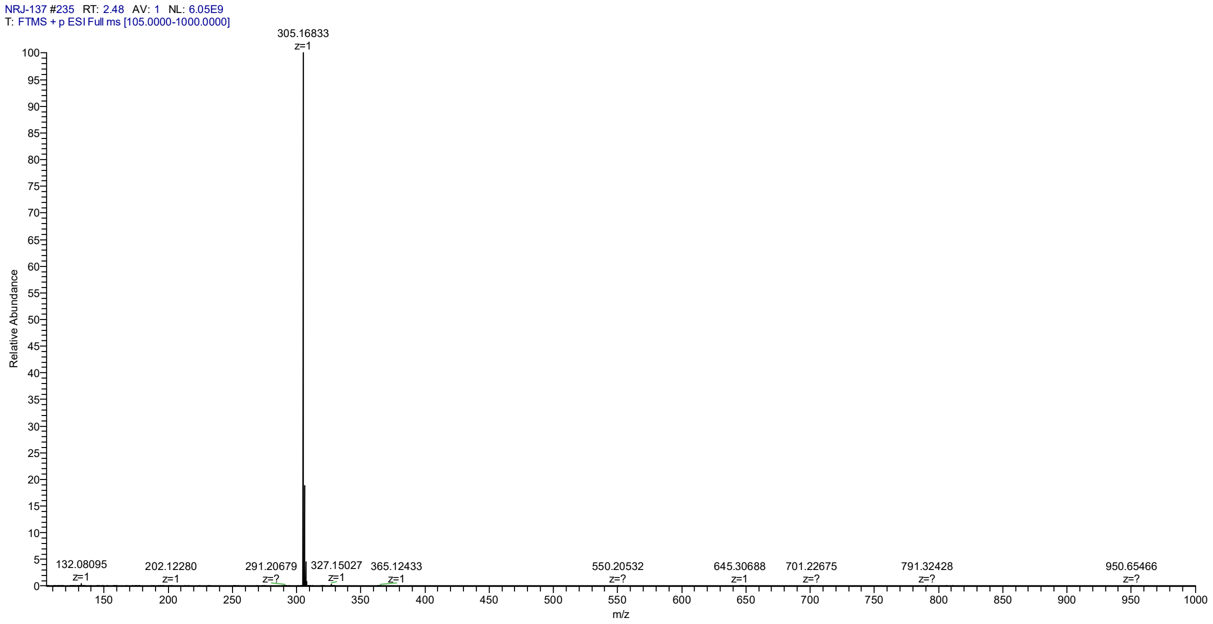

Figure S48 - HRMS spectrum for 6e.

1.13 1-(4-(Diethylamino)butyl)-3,4-dihydroquinolin-2(1*H*)-one (6f)

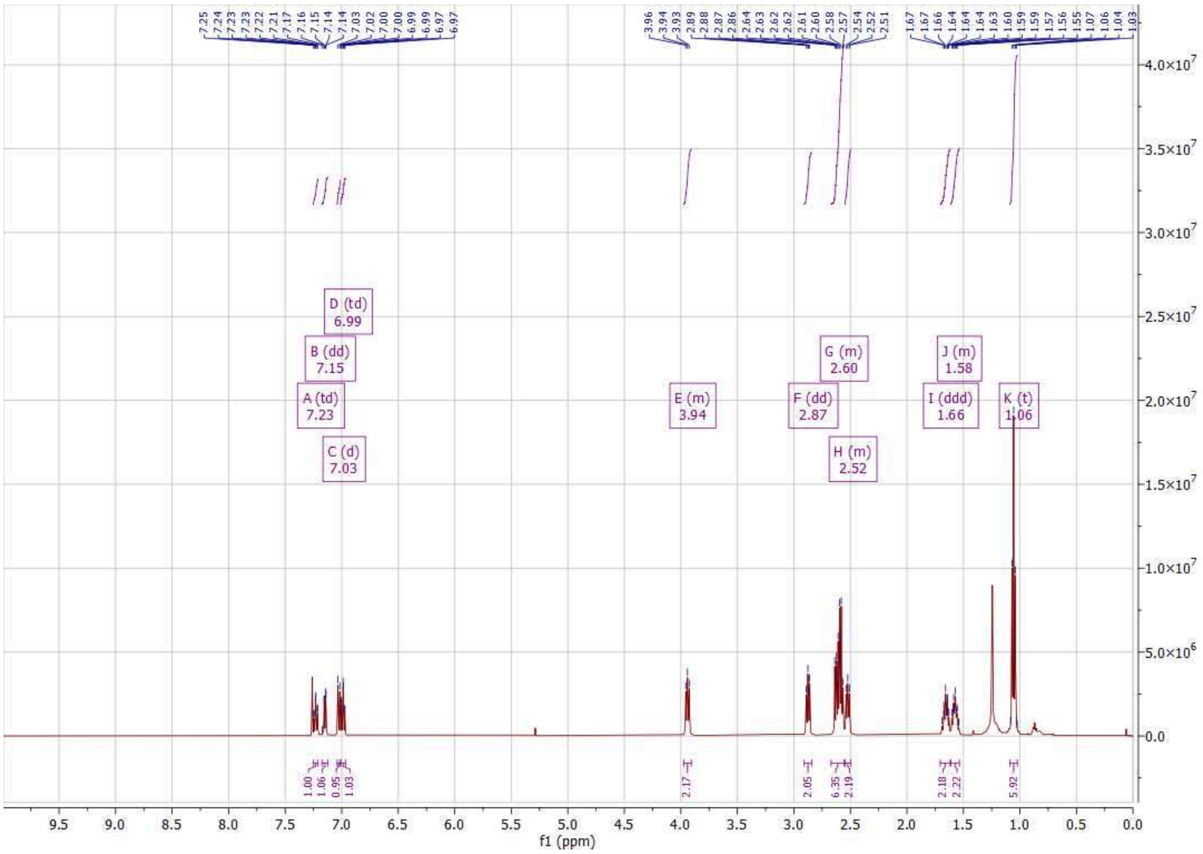

Figure S49 - ^{1}H NMR spectrum for 6f.

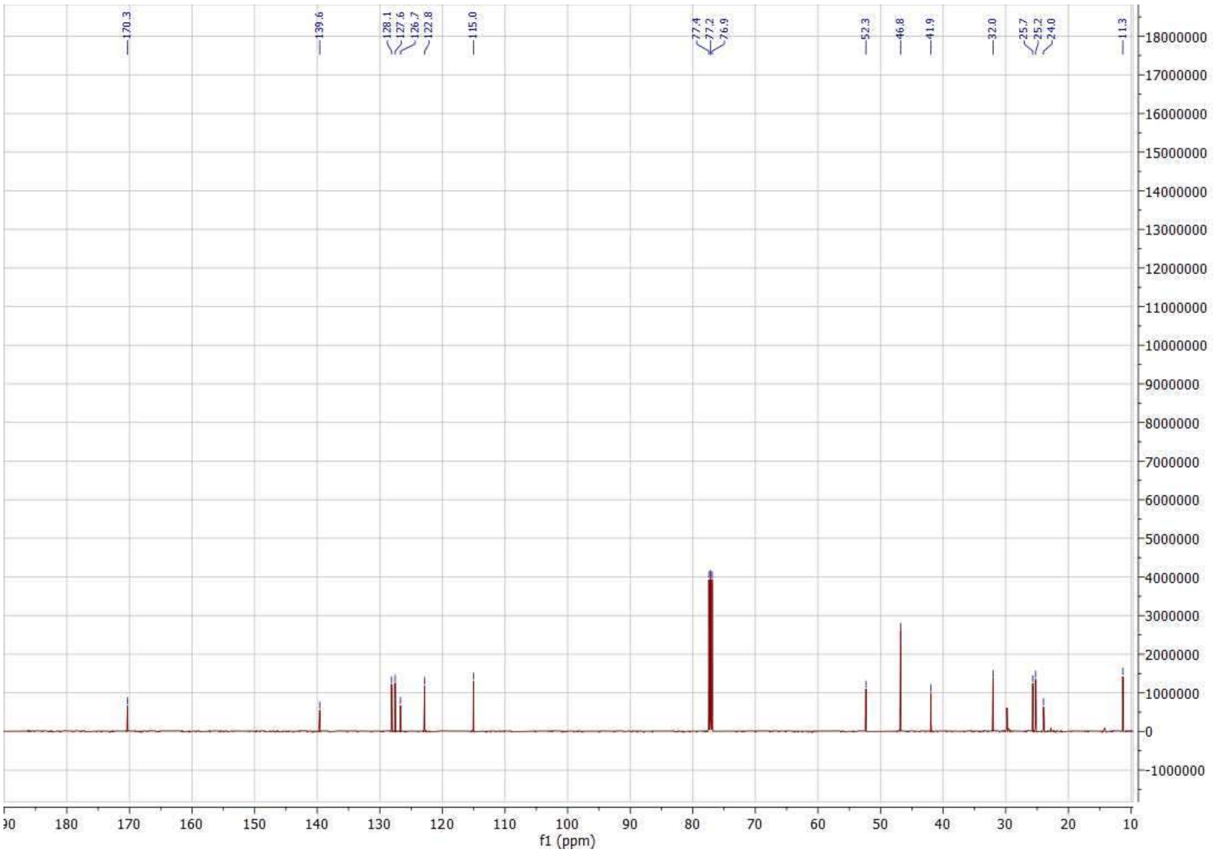

Figure S50 - ^{13}C NMR spectrum for 6f.

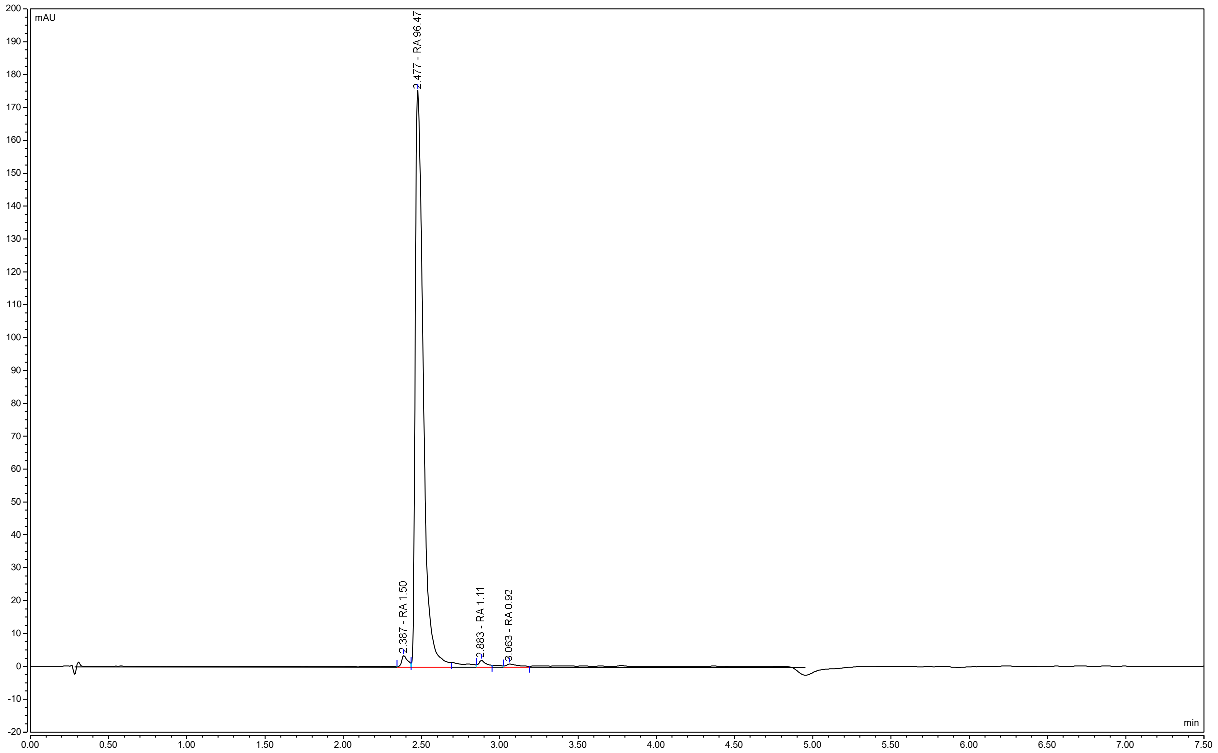

Figure S51 – UV-LC chromatogram for 6f.

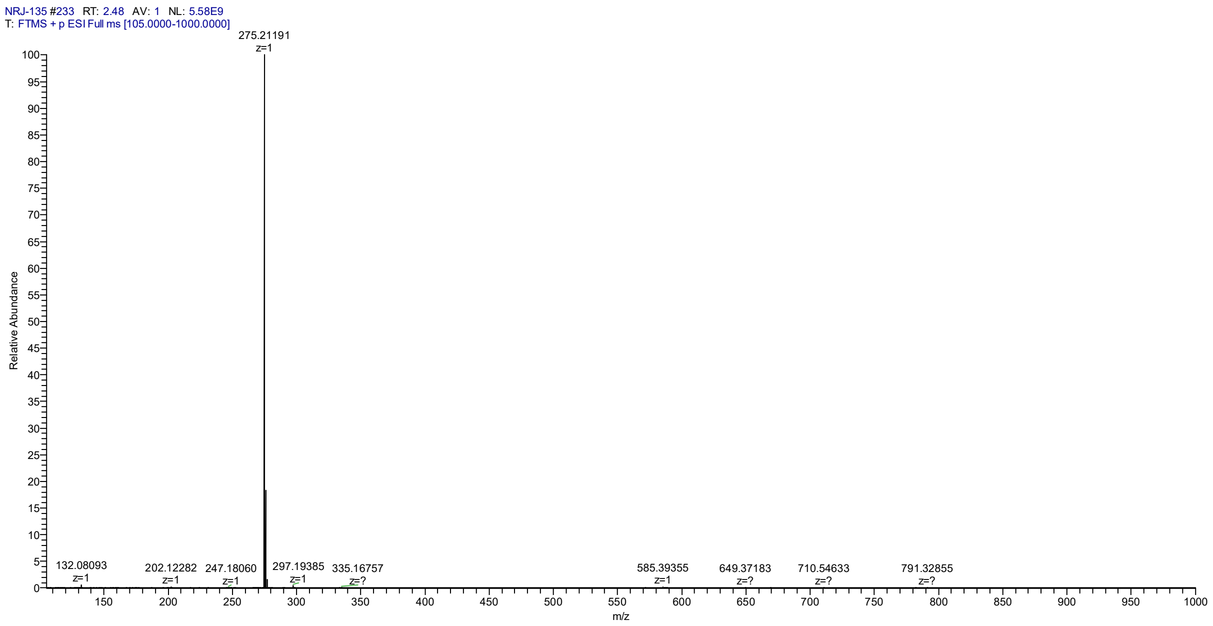

Figure S52 - HRMS spectrum for 6f.

1.14 1-(4-((2-Methoxyethyl)(methyl)amino)butyl)-3,4-dihydroquinolin-2(1*H*)-one (6g)

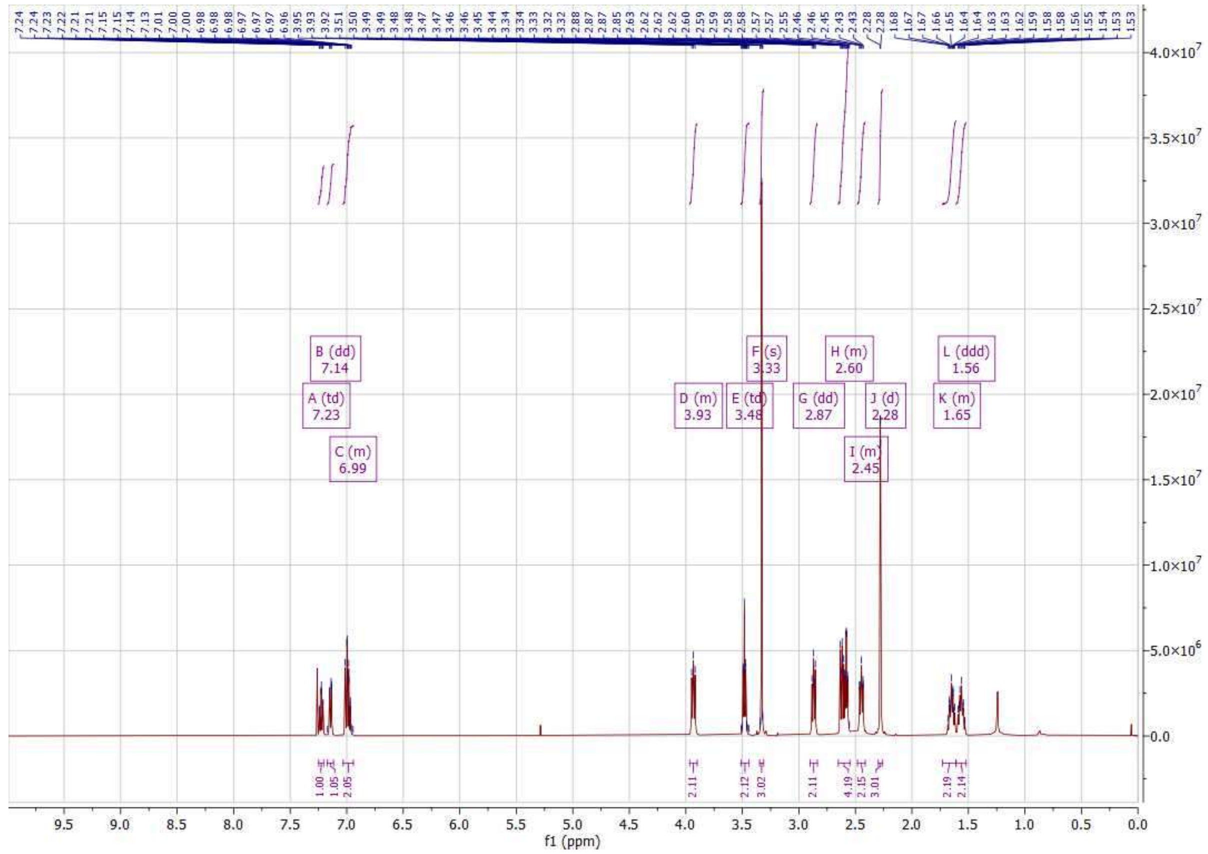

Figure S53 - ^{1}H NMR spectrum for 6g.

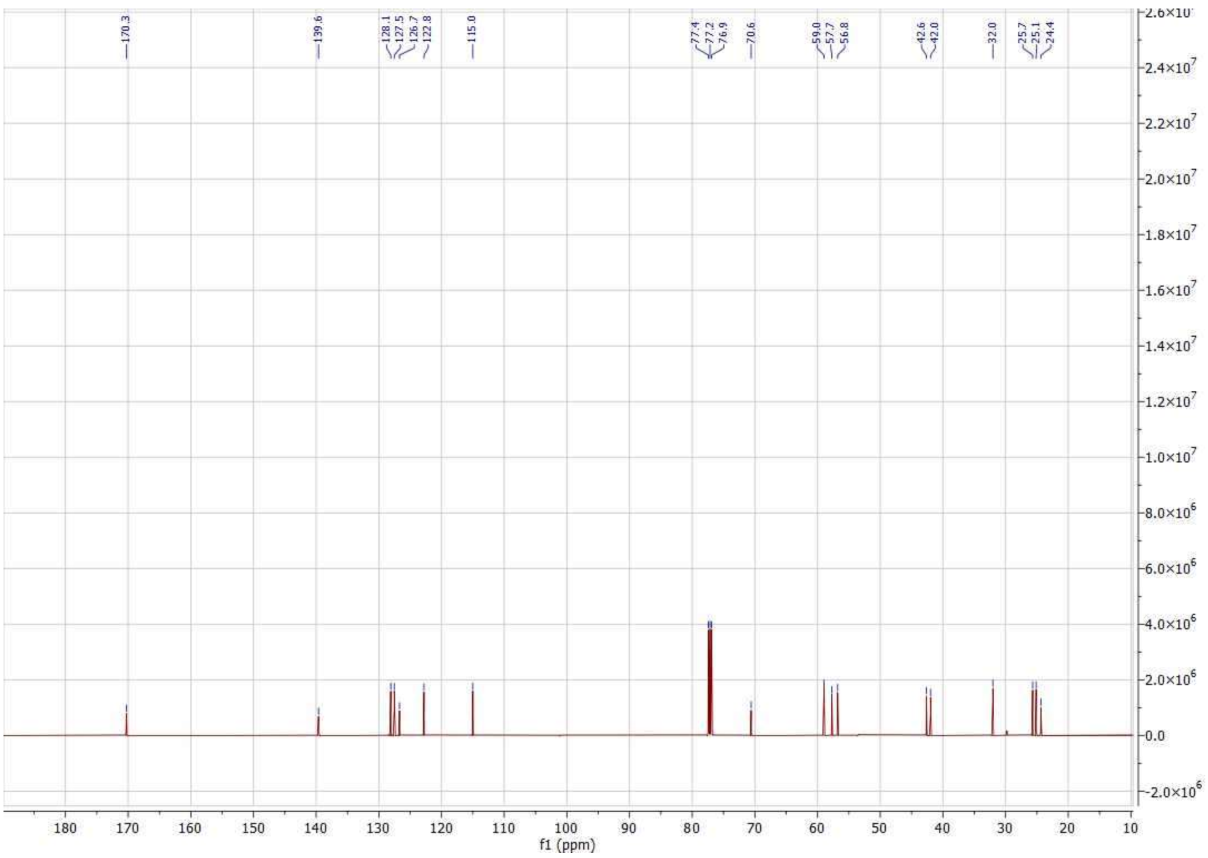

Figure S54 - ^{13}C NMR spectrum for 6g.

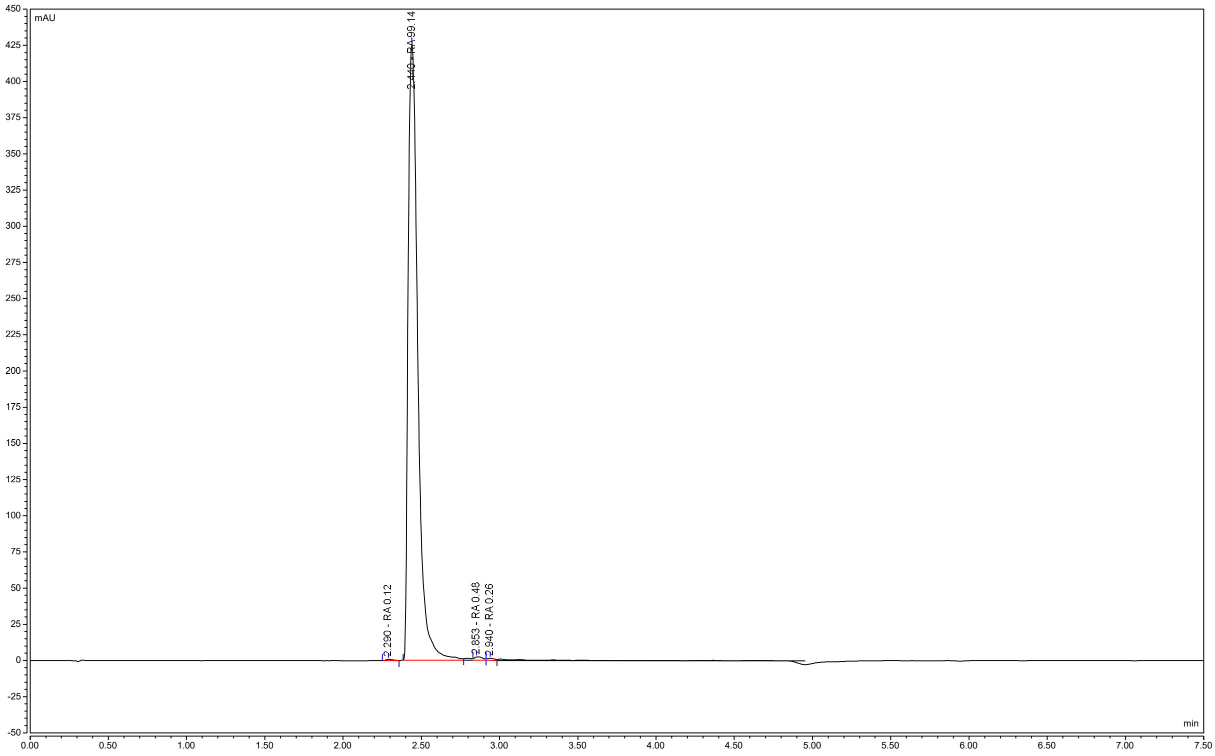

Figure S55 – UV-LC chromatogram for 6g.

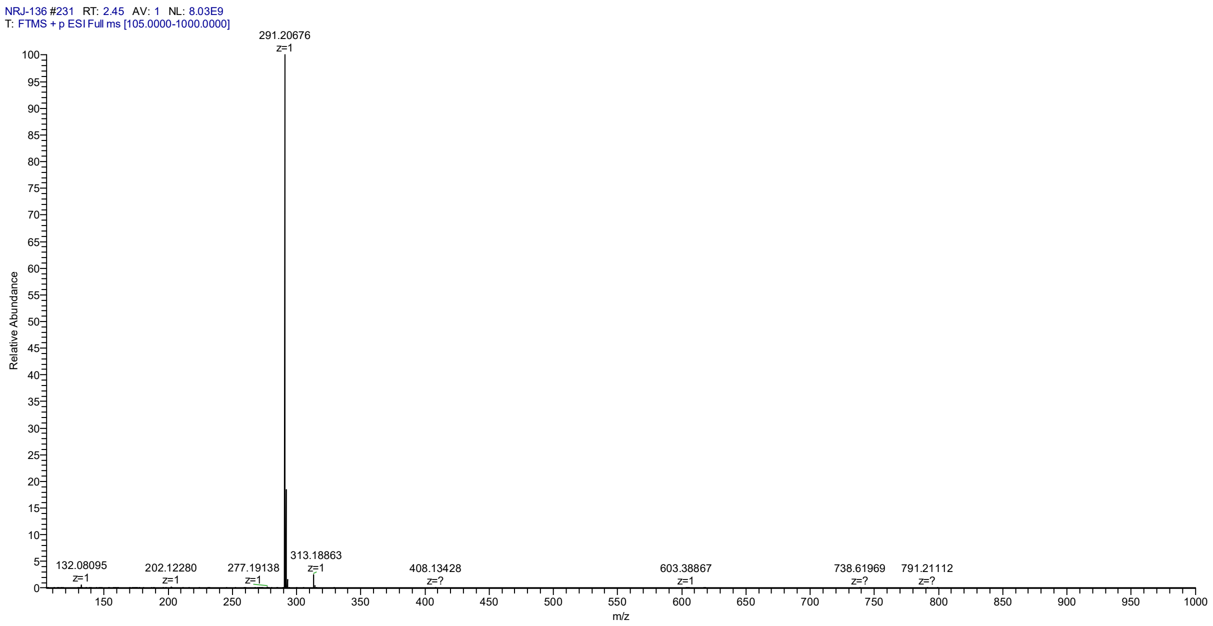

Figure S56 - HRMS spectrum for 6g.

2.Biological testing

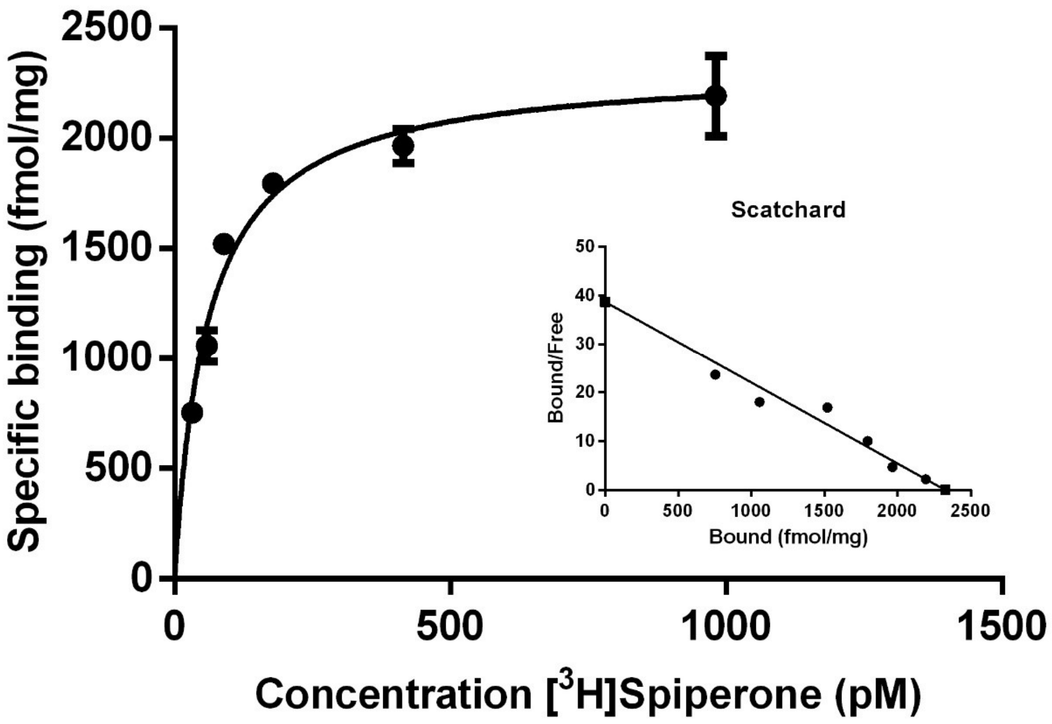

Figure S57 - Saturation binding curve and Scatchard plot of the affinity of [^{3}H]spiperone to D_2Rs.

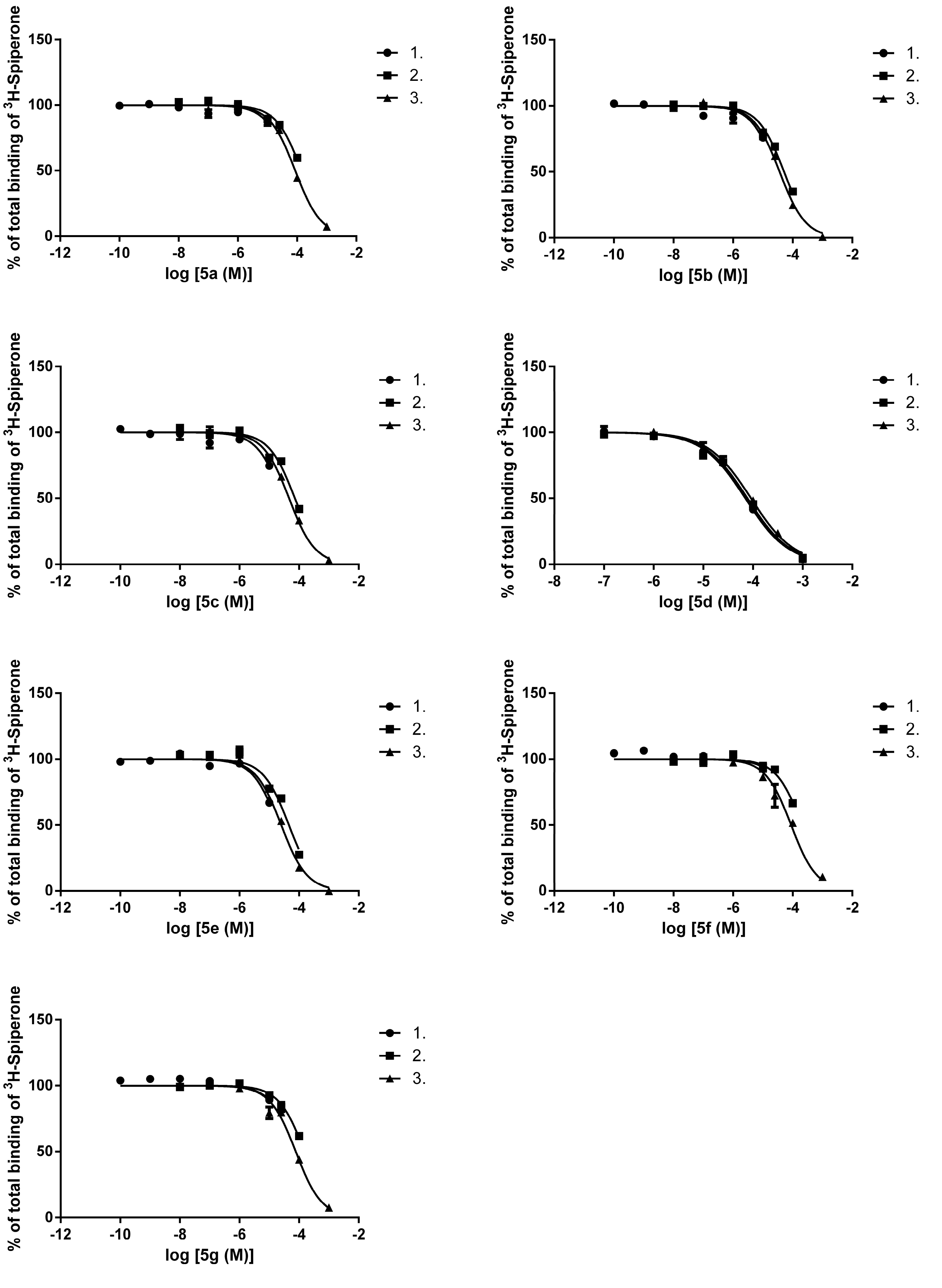

Figure S58 - Inhibition of [^{3}H]spiperone binding to D_2R_S receptors by 3,4-dihydroquinolin-2(1*H*)-one derivatives (5a – 5g). Each point represents mean ± SD, n = 3.

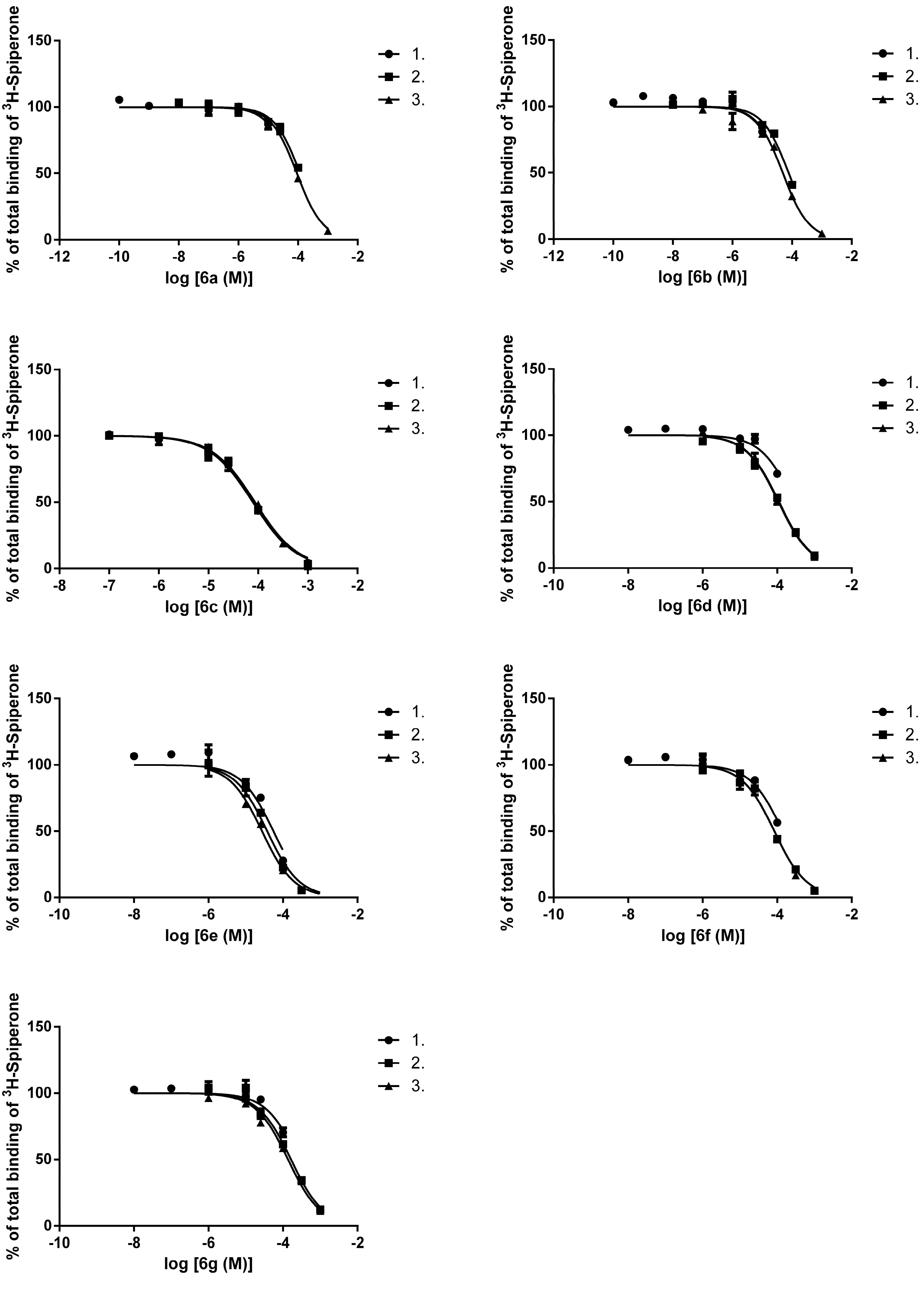

Figure S59 - Inhibition of [^{3}H]spiperone binding to D_2R_S receptors by 3,4-dihydroquinolin-2(1*H*)-one derivatives (6a – 6g). Each point represents mean ± SD, n = 3.

Table S1 - Functional activities of **5a**-**g** and **6a**-**g** at D_2Rs. Values represent mean ± S.E.M. of 3 independent experiments performed in triplicate.

Compound	IC_{50} in µM ± SEM
**5a**	98 ± 23
**5b**	39 ± 6.4
**5c**	49 ± 13
**5d**	80 ± 6.3
**5e**	30 ± 7.7
**5f**	150 ± 34
**5g**	106 ± 24
**6a**	93 ± 14
**6b**	57 ± 10
**6c**	82 ± 2.3
**6d**	166 ± 56
**6e**	40 ± 8.2
**6f**	103 ± 19
**6g**	188 ± 38

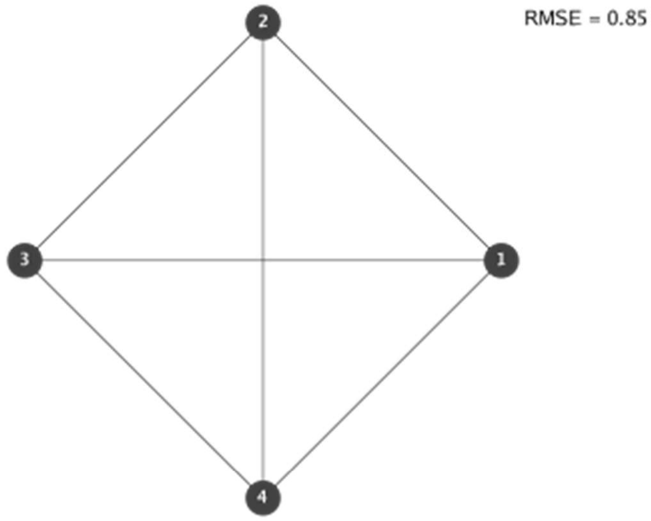

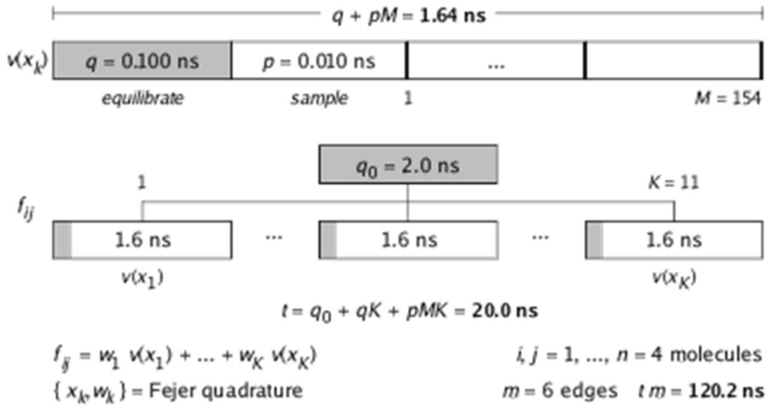

Figure S60: Schematic representation of the settings used in the Thermodynamic Integration Free Energy Calculation. "q0" is the simulation time of the master equilibration, "q" is the simulation time of the λ-window equilibration, "p" is the sample period, "K" is the amount of dynamics simulations, "M" is the total amount of samples. Together, this means the entire ensemble of simulations is in a period of 120.2 ns. Figure was generated using MOE.
